# Erratum to: Rapid transcriptional plasticity of duplicated gene clusters enables a clonally reproducing aphid to colonise diverse plant species

**DOI:** 10.1186/s13059-017-1202-6

**Published:** 2017-04-04

**Authors:** Thomas C. Mathers, Yazhou Chen, Gemy Kaithakottil, Fabrice Legeai, Sam T. Mugford, Patrice Baa-Puyoulet, Anthony Bretaudeau, Bernardo Clavijo, Stefano Colella, Olivier Collin, Tamas Dalmay, Thomas Derrien, Honglin Feng, Toni Gabaldón, Anna Jordan, Irene Julca, Graeme J. Kettles, Krissana Kowitwanich, Dominique Lavenier, Paolo Lenzi, Sara Lopez-Gomollon, Damian Loska, Daniel Mapleson, Florian Maumus, Simon Moxon, Daniel R. G. Price, Akiko Sugio, Manuella van Munster, Marilyne Uzest, Darren Waite, Georg Jander, Denis Tagu, Alex C. C. Wilson, Cock van Oosterhout, David Swarbreck, Saskia A. Hogenhout

**Affiliations:** 1 Earlham Institute, Norwich Research Park, Norwich, NR4 7UZ UK; 2 John Innes Centre, Norwich Research Park, Norwich, NR4 7UH UK; 3 The International Aphid Genomics Consortium, Miami, USA; 4 INRA, UMR 1349 IGEPP (Institute of Genetics Environment and Plant Protection), Domaine de la Motte, 35653 Le Rheu Cedex, France; 5 IRISA/INRIA, GenOuest Core Facility, Campus de Beaulieu, Rennes, 35042 France; 6 Univ Lyon, INSA-Lyon, INRA, BF2I, UMR0203, F-69621 Villeurbanne, France; 7 School of Biological Sciences, University of East Anglia, Norwich Research Park, Norwich, NR4 7TJ UK; 8 CNRS, UMR 6290, Institut de Génétique et Developpement de Rennes, Université de Rennes 1, 2 Avenue du Pr. Léon Bernard, 35000 Rennes, France; 9 Department of Biology, University of Miami, Coral Gables, FL 33146 USA; 10 Centre for Genomic Regulation (CRG), The Barcelona Institute of Science and Technology, Dr. Aiguader 88, Barcelona, 08003 Spain; 11 Universitat Pompeu Fabra (UPF), 08003 Barcelona, Spain; 12 Institució Catalana de Recerca i Estudis Avançats (ICREA), Pg. Lluís Companys 23, 08010 Barcelona, Spain; 13 Unité de Recherche Génomique-Info (URGI), INRA, Université Paris-Saclay, 78026 Versailles, France; 14 INRA, UMR BGPI, CIRAD TA-A54K, Campus International de Baillarguet, 34398 Montpellier Cedex 5, France; 15 Boyce Thompson Institute for Plant Research, Ithaca, NY 14853 USA; 16 School of Environmental Sciences, University of East Anglia, Norwich Research Park, Norwich, NR4 7TJ UK; 17 Present Address: INRA, UMR1342 IRD-CIRAD-INRA-SupAgro-Université de Montpellier, Laboratoire des Symbioses Tropicales et Méditéranéennes, Campus International de Baillarguet, TA-A82/J, F-34398 Montpellier cedex 5, France; 18 Present address: Rothamsted Research, Harpenden, Hertforshire ALF5 2JQ UK; 19 Present address: J. R. Simplot Company, Boise, ID USA; 20 Present address: Alson H. Smith Jr. Agriculture and Extension Center, Virginia Tech, Winchester, 22602 VA USA; 21 Present address: Department of Plant Sciences, University of Cambridge, Downing Street, Cambridge, CB2 3EA UK; 22 Present address: Moredun Research Institute, Pentlands Science Park, Bush Loan, Penicuik, Midlothian EH26 0PZ UK

## Erratum

After publication of this article [1] we noticed that reference 50 was incorrect. The correct reference 50 is as follows:

Santamaría S, Gonzalez-Cabrera J, Martinez M, Grbic V, Castanera P, Diaz L, Ortego F. Digestive proteases in bodies and faeces of the two-spotted spider mite, *Tetranychus urticae*. J Insect Physiol. 2015; 78:69–77 http://www.sciencedirect.com/science/article/pii/S0022191015001018
