# Supplementary material for: Rapid transcriptional plasticity of duplicated gene clusters enables a clonally reproducing aphid to colonise diverse plant species
Source: Genome Biol. 2017 Feb 13;18:27. doi: 10.1186/s13059-016-1145-3 (PMC5304397; doi:10.1186/s13059-016-1145-3)
Supplement: Additional file 2: — Supplementary Text: Genome assembly, annotation and quality control. (DOCX 2215 kb) [file 13059_2016_1145_MOESM2_ESM.docx]

**Additional File 2: Genome assembly, annotation and quality control**

**Genome assembly**

The genomes of *M. persicae* clones G006 and O were independently assembled using a combination of short insert paired-end and mate-pair libraries (Additional File 1: Table S1). For clone G006, each dataset was first error corrected using Quake [1] and assembled using ALLPATHS -LG [2] using the default parameters. For clone O, we trimmed reads from both paired-end libraries (LIB1672 and LIB1673) using sickle (https://github.com/najoshi/sickle) to keep only reads or read fragments that have a quality score consistently over 20. These libraries were then assembled using ABySS [3] with a kmer value of 71. Mate-pair reads (LIB1472) were trimmed and filtered to remove adaptor sequences and then used with the previous ABySS assembly by SSPACE [4] to produce longer scaffolds.

To identify duplications in the assemblies arising from heterozygosity we aligned the set of scaffolds against each other removing putatively duplicated regions if they were larger than 1kb with a percentage identity higher than 90. This resulted in 184 scaffolds being removed from the G006 assembly. The same approach was used to remove heterozygosity from the clone O assembly but an identity threshold of 99 percent identity was used instead, resulting in the removal of 43Mb of sequence. A kmer analysis verified removal of redundant content. Examination of both assemblies showed a fraction of sequences with either higher coverage and/or a higher GC percentage, subsequent BLASTn searches identified these as sequence of bacterial origin including *Buchnera aphidicola* and *Pseudomonas* spp. contamination, identified sequences were removed from the *M. persicae* assemblies.

***Assembly of the clone G006* Buchnera aphidicola *endosymbiont genome***

Examination of initial assemblies identified a fraction of sequences with either higher coverage and / or a higher GC percentage. BLAST searches revealed the majority of sequences to have hits to the primary aphid endosymbiont *B. aphidicola*. In the Allpaths-LG assembly, we found 168 scaffolds that include part of the *B. aphidicola* genome. These sequences were short with the sum of all scaffolds lower than the expected *B. aphidicola* genome size (642 011bp for the *A. pisum* LSRA strain) (NZ_ACFK01000001.1). To improve the *B. aphidicola* genome assembly we proceeded to generate a specific assembly of the *B. aphidicola* genome using Minia [5] with increased min_abundance and Kmer_size thresholds. Although this stringency is too high to build the nuclear genome because its coverage is too low, it is efficient to assemble the *B. aphidicola* genome. Two scaffolds were retrieved with similarity to *B. aphidicola* (264603 and 378928 bp). These scaffolds when aligned correspond to 91% of the *B. aphidicola* str. LSR1 (*A. pisum*) genome. The *M. persicae* clone G006 *B. aphidicola* genome was deposited under BioProject accession PRJNA319804 and is also available from www.aphidbase.com.

**Genome annotation**

***Repetitive elements***

Repetitive elements were annotated with the REPET package (v2.0). The TEdenovo pipeline [6] from REPET was used to build libraries of consensus sequences representative of repetitive elements. Whole genome assemblies were used separately as input for TEdenovo. Consensus sequences were built if at least three similar copies were detected. Each consensus was classified with PASTEC [7] followed by semi-manual curation. The libraries from each assembly were used for genome annotation of the respective assembly with the TEannot pipeline [8] from REPET to select the consensus sequences that are present for at least one full-length copy. The selected consensus were pooled and redundancy was removed with parameters of length >= 98% and identity >= 95%. The non-redundant library was finally used to perform genome annotation of each strain with TEannot using BLASTER sensitivity = 3. In addition, RepeatModeler (v1-0-7) ([http://www.repeatmasker.org/RepeatModele​r.html](http://www.repeatmasker.org/RepeatModeler.html)) was used to generate a species specific repeat library based on the clone O assembly. Interspersed repeats were identified using Repeatmasker (v4.0.3 – rmblast-2.2.27) (<http://www.repeatmasker.org/>) with the *Myzus persicae* repeat library and Repbase Insecta library (repeatmaskerlibraries-20130422). Low complexity repeats were identified with RepeatMasker (v4.0.3 – rmblast-2.2.27). The same repeat libraries were used to identify interspersed repeats from the G006 assembly.

***Protein coding genes***

Protein coding genes were predicted by an evidence based gene annotation approach utilizing the AUGUSTUS [9] gene predictor with protein, cDNA and RNA-Seq alignments. Protein sequences from 6 species *Rhodnius prolixus, Pediculus humanus, Bombix mori, Apis mellifera, Daphnia pulex* and *Acyrthosiphon pisum* were soft masked for low complexity (segmasker) and aligned to the softmasked (interspersed repeats) clone O and G006 assemblies with exonerate protein2genome (v-2.2.0) [10], alignments were filtered at a minimum 60% identity and 50% coverage. Additionally, a total of 27,826 public EST and CDNA sequences (Genbank) were softmasked (dustmasker) and aligned to the softmasked (interspersed repeats) clone O and G006 assemblies with exonerate est2genome (v-2.2.0) [10], alignments were filtered at a minimum 95% identity and 50% coverage. Strand-specific RNA-Seq reads from clone O adults and nymphs (LIB1777), bidirectional RNA-Seq reads of clone O colonies reared on Chinese cabbage (*Brassica rapa* (Br); Brassicaceae) and *N. benthamiana* (Nb, Solanaceae) (LIB949-LIB954) and strand specific reads from whole clone G006 adult insects, isolated guts and bacteriocytes (Additional File 22: Table S7) were aligned with Tophat (v2.0.9) [11] and assembled with Cufflinks (v2.0.2) [12]. Reads were filtered for quality and rRNA (trim_galore-0.3.3: -q 20 --stringency 5 --length 60, sortmerna-1.9: -r 0.25) (<http://www.bioinformatics.babraham.ac.uk/projects/trim_galore/>).

AUGUSTUS [9] is a program that predicts genes in eukaryotic genomic sequence by means of a Generalized Hidden Markov Model (GHMM) that takes both intrinsic and extrinsic information into account. To train the AUGUSTUS *ab initio* model we compiled a set of training and test genes. Strand specific RNA-Seq (LIB1777) from clone O adults and nymphs were assembled relative to the clone O reference with Trinity (v2013_08_14) [13] in genome guided mode. *M. persicae* cDNAs, ESTs and RNA-Seq assemblies were used to construct sub clusters with PASA-v20130907 (Program to Assemble Spliced Alignments) [14] and ORFs > 100 amino acids with both 5’ and 3’ UTR features were extracted. We applied a strict filtering approach to identify a subset of genes with bona fide gene structures. Genes with a genomic overlap (within 1000bp of a second gene) were excluded and ORFs were assessed by alignment to *A. pisum* gene models (requiring >70% identity, alignment length > 80% of query length, query length >80% of target length, less than 5 alignment gaps and identical start positions). A final filtering step removed genes that were greater than 80% identical and a single model was selected for each locus. AUGUSTUS was trained and evaluated versus a test set of 100 genes achieving 0.975 sn, 0.857 sp nucleotide level, 0.907 sn, 0.799 sp exon level and 0.4 sn, 0.323 sp at the gene level.

AUGUSTUS gene models were predicted using the trained *ab initio* model with the 6 sets of cross species protein alignments, RNA-Seq junctions (defining introns), and Cufflinks alignments as evidence hints. RNA-Seq read density was provided as exon hints and repeat information (interspersed repeats) as nonexonpart hints. From manual review we identified occasional over extension of UTRs due to intergenic or intronic reads present in the ribodepleted strand specific library in response we generated two alternative Augustus models by either including or excluding the RNA-Seq read depth information. Additionally we generated models using Maker to integrate evidence set [15], all alignments were provided to Maker via the GFF pass through option. Augustus was selected as the gene predictor using the trained ab initio model.

A set of integrated gene models was derived from the AUGUSTUS (x2) and Maker gene predictions along with the transcriptome and protein alignments using EVidenceModeler:v20120625 (EVM) [16]. Weights of evidence were manually set following an initial testing and review process as: ABINITIO_PREDICTION 1, PROTEIN 4, TRANSCRIPT 7. We identified examples of EVM errors resulting from incomplete genes in the AUGUSTUS / Maker gene predictions or non-canonical splicing, to rectify these problems we substituted the EVM model for the overlapping Augustus model (with RNA-Seq defined exons) and additionally incorporated any Maker models with an annotation edit distance (AED) score between 0 and 0.5.

To add UTR features and alternative splice variants we ran PASA [14] with a subset of the assembled RNA-Seq cufflinks assemblies and available *M. persicae* ESTs/cDNAs using the EVM models as the reference annotation. We excluded cufflinks assemblies that lacked strand orientation i.e. single exon transcripts derived from the non-strand specific libraries and filtered out those that were found to contain multiple ORFs (and therefore may be chimeric) and where the CDS to mRNA length ratio was found to be below 30% or where the UTR length was greater than the mean UTR length + standard deviation.

The PASA updated EVM models were further refined by removing transcripts that showed no expression support (using all available RNA-Seq libraries) or had no support from cross species protein alignments. Transcripts were also excluded if they aligned with ≥ 30% similarity and 40% coverage to a TransposonPSI (v 08222010) library (http://transposonpsi.sourceforge.net/) and also demonstrated ≥ 40% coverage by the RepeatModeler / RepeatMasker derived interspersed repeats. In addition, transcripts that had ≥ 30% similarity and 60% coverage to the TransposonPSI library or had ≥ 60% coverage by the RepeatModeler / RepeatMasker derived interspersed were also excluded. We retained any transcripts that aligned with 60% identity with a set of NCBI *A. pisum* proteins (http://www.ncbi.nlm.nih.gov/genome/annotation_euk/Acyrthosiphon_pisum/101/) filtered to remove TE related genes. Proteins were excluded from NCBI *A. pisum* if they aligned with ≥ 30% similarity and ≥ 60% coverage to TransposonPSI at 1e-10 and if they had description matching to transposon-related/un-characterized protein (6,788 proteins were excluded from 23,090 NCBI *A. pisum* proteins). Additional, manual annotation of genes involved in metabolism pathways and genes implicated in host adjustment was also carried out. Full details are given in Additional File 3.

***Non-coding RNAs***

tRNA genes were predicted by tRNAscanSE-1.3.1 with eukaryote parameters [17] and rRNAs using rnammer-1.2 [18]. miRNA was predicted by BLASTN searches with precursor miRNAs from miRBase 21.0 [Kozomara, 2014] against the *M. persicae* clone G006 and clone 0 genome sequences (BLAST 2.2.30, E-value 10E-6). Putative miRNA precursors were folded using RNAfold [19] and small RNA sequencing data was aligned to precursor hairpins using PatMaN [20]. Both RNA secondary structure and small RNA read alignments were checked manually to ensure they were consistent with precise Drosha and Dicer processing. Reads from the small RNA sequencing were then checked against miRBase using miRProf [21] and any mature miRNA reads present that were not identified through the BLAST search were mapped to the genome sequences directly using PatMaN and a flanking window of 300nt upstream and downstream of the genomic match was extracted. All small RNA reads were mapped to this sequence window and the precursor miRNA was identified manually from the read alignments and secondary structures. Novel miRNAs were identified using processed small RNA reads as input to miRCat [21], which was run using default animal parameters. miRBase homologues identified in the BLAST search were filtered out of the results and the remaining predictions were checked manually for secondary structure and read alignments as described above.

**Assembly and Annotation Quality Control**

The *M. persicae* G006 genome was assembled from a total of 182 million paired end reads, 140 million 5 kb mate pair reads and 152 million 2 kbp mate pair reads (Additional File 1: Table S1). Using a kmer-based approach on the raw sequence reads we estimate the genome size to be 421.6 Mbp, which is smaller than the 533 Mbp *A. pisum* (pea aphid) genome (version 2.1b). The size of the assembled *M. persicae* genome was 347 Mbp including ambiguous bases, representing over 82% of the total genome. The assembly consists of 4,018 contigs >1 kb with an N50 scaffold length of 435 kb and an average coverage of 51x (main text Table 1). A total of 341 Mbp (98% of the assembled genome) was contained in 1844 scaffolds >10 kb. The longest contig assembled was over 2,199 kb. Assembly contiguity is comparable with the scaffold N50 for version 2.1b of the *A. pisum* assembly (519 Kb), although assembly size is significantly smaller (Figure 1). The genome of *M. persicae* clone O was assembled into 13,407 scaffolds (≥1,000 bp) from a total of 163 million paired end reads, 165 million overlapping paired end reads and 220 million 8 kb mate-pair reads (Additional File 1: Table S1). Kmer analysis of the paired end reads generated for the UK clone O (LIB1672) and US clone G006 (S6) shows higher levels of genomic heterogeneity in the UK clone O (Figure 2). In addition, the US clone G006 has a slightly higher coverage and a better separation between k-mers likely to originate from sequencing errors and those from genuine genomic content. These factors contribute to a less contiguous assembly with a lower N50 in *M. persicae* clone O compared to the *M. persicae* clone G006 genome, and more evidence of genes being fragmented across scaffolds (Figure 3).

**
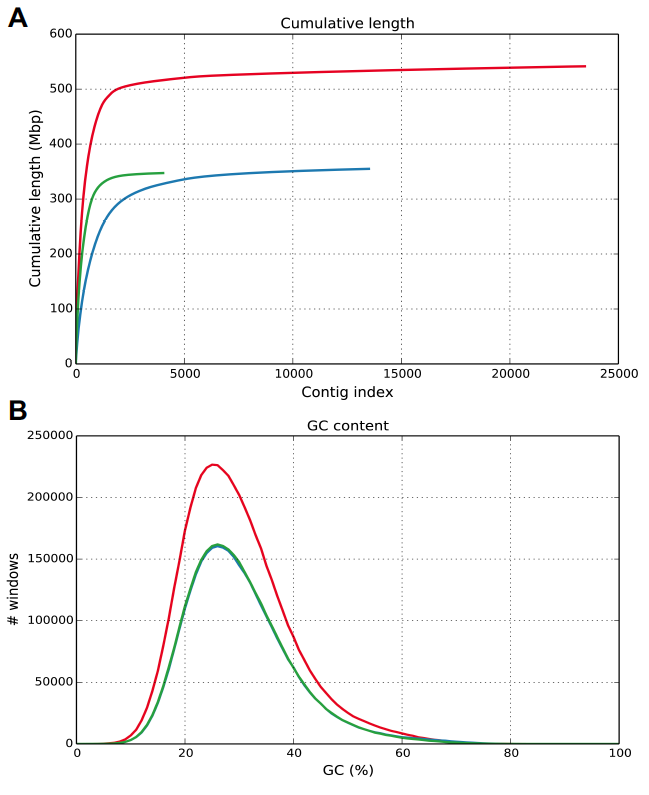
**

**Figure 1: A**. Cumulative length distribution plots for *M. persicae* clones G006 (green line), *M. persicae* clone O (blue line) and *A. pisum* V2.1b (red line). The plot shows the relative contiguity of each size-sorted assembly. Both *M. persicae* assemblies are similar sizes with the G006 assembly being more contiguous. The *A. pisum* assembly is around 54% larger than *M. persicae* and has a higher N50 despite having a large number of very small sequences. **B**. The GC distribution of content within the assemblies of *M. persicae* clone G006 (green line) and *A. pisum* V2.1b (red line). All aphid assemblies have near identical GC distributions, although the larger size of the *A. pisum* separates it from the other assemblies in this plot.


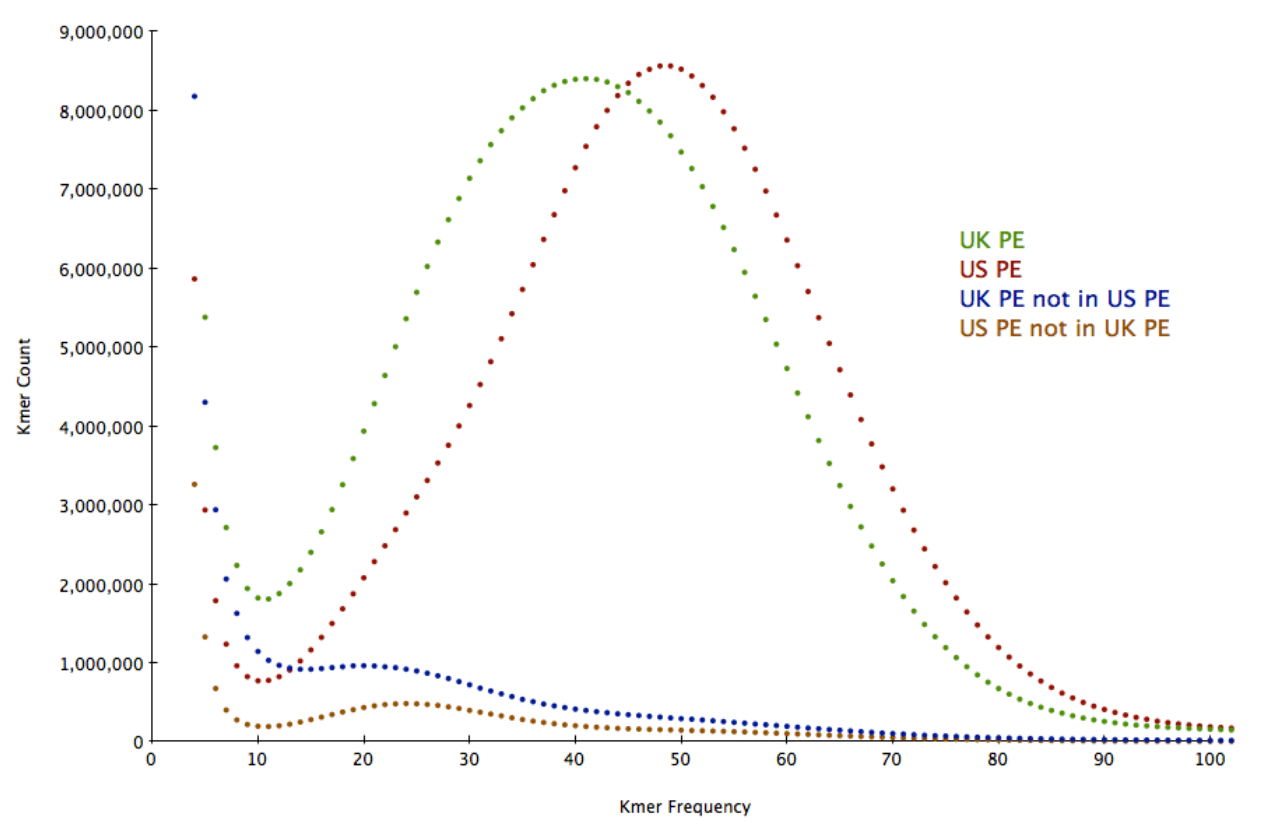


**Figure 2:** Comparison of the kmer content of paired end reads between *Myzus persicae* UK clone O (LIB1672) and US clone G006 (S6). The G006 dataset has a higher coverage and a more defined separation between probable sequencing errors (left hand peak) and probably genome content (centre peak) than clone O. In addition, clone O seems to contain more heterogeneous content than G006, as shown by the small peaks in the content that is distinct to each dataset.

**Figure 3:** G006 gene models were mapped to the Clone 0 assembly using GMAP (v20140220) and alignments filtered at 95% identity. The transcript coverage was analysed at per scaffold level as well as overall transcript coverage across all scaffolds. The same analysis was carried out for Clone 0 annotation versus the Clone G006 assembly. Alignments indicate the Clone O gene space to be slightly more fragmented across scaffolds with 95.19% of Clone O genes aligning with >=80% coverage to the G006 assembly compared to 87.86% of G006 genes to the clone O assembly at per scaffold level.

Completeness of the two assembled *M. persicae* genomes was assessed using CEGMA (Core Eukaryotic Genes Mapping Approach, v2.5) [22] to search for conserved single-copy eukaryotic orthologs. Using this approach, the G006 assembly was determined to be 94.35% and the clone O assembly 94.76% complete with 234 out of 248 and 235 out of 248 test genes recovered as full length, respectively. Including partial genes, this increases to 98.39% complete for both the clone O and G006 assemblies with 244 test genes recovered. As an additional check of assembly completeness we aligned a set of 1,349 *A. pisum* proteins identified as having a single copy ortholog present in >90% of arthropods (OrthoDB) and 79,898 transcripts of ≥1 kb assembled *de novo* from 182,928,264 million *M. persicae* RNA-Seq reads. In excess of 95% of the proteins could be aligned (minimum of 90% coverage and 70% identity) to the G006 and clone O assemblies and greater than 90% of the transcripts (minimum 70% coverage and 95% identity).

***Repeat elements***

Repeat elements constitute approximately 87 Mb (25%) of the G006 assembly, which is in the range of what was previously observed in insect genomes of comparable size [23]. The most abundant repeats correspond to transposable elements (TEs), unclassified repeats, and tandemly repeated sequences (Figure 4). Among TEs, class 2 DNA elements are predominant, which is similar to what was observed in the pea aphid genome assembly (Figure 5). Among class 2 DNA elements the hAT-type superfamily are predominant in both clone O and G006 assemblies with a slightly greater quantity present in the more contiguous G006 assembly (Figure 5).

TEs

TEs

0 20 40 60 80 100 Mb

TEs

**Figure 4:** Contribution of repetitive elements in *M. persicae* clone O and G006 assemblies. A *de novo* whole genome annotation of repetitive elements in the O and G006 assemblies was performed with the REPET package (v2.0).

0 2 4 6 8 Mb

**Figure 5:** Contribution of Class 2 DNA elements in *M. persicae* clone O and G006 assemblies.

***Gene content***

Similar numbers of protein coding genes and transcripts were annotated on the two assemblies (G006 18,529 protein coding genes, 30,127 isoforms; clone O 18,433 protein coding genes, 30,247 isoforms) in line with the ~15-20k genes reported in other insect species but lower than the 36,939 genes annotated in the latest release of the *A. pisum* genome (Table 1 main text). *A. pisum* and the waterflea *Daphnia pulex* both contain a large proportion of genes (36% and 37%, respectively, of over 30,000 genes) with no significant hits to genes identified in other insect species. Unsupported *ab initio* models, partial genes and assembly duplication artifacts contribute to the inflated *A. pisum* gene count [24]. A more restrictive annotation based on strongly supported predicted genes of *A. pisum* produced a catalog of approx. 20,000 RefSeq gene models (NCBI, RefSeq database). Our analysis of these models indicates that the *A. pisum* annotation contains a significant number of transposable element (TE) related genes (5,151 hits from psi-blast searches versus a TE dataset at 30% similarity and 50% coverage) (Figure 6), in line with gene family analysis showing the largest gene family expansions to be in TE related families with 50 - 209 members [25]. To assess if genes were missing from the *M. persicae* annotation we aligned proteins from *A. pisum* (AphidBase 2.1b release) to the *M. persicae* G006 assembly, 73.42% (26,576) of proteins were aligned with high stringency (≥60% identity and ≥50% coverage) of which 82.18% (21,840) overlapped annotated *M. persicae* gene models. Lower stringency alignments (BLAST e-value 1e-5) identified a further 6,348 *A. pisum* proteins with hits to the *M. persicae* reference. Of the 32,924 (91%) *A. pisum* proteins with either a high or low stringency alignment, only 7% (2,452) are located outside of annotated genes. Of these 41% (1,007) show similarity to TEs (≥30% similarity to TransposonPSI TE library or via RepeatMasker). Of the remaining 1,445 potentially missing proteins, 36% (884) align with greater than 70% overall coverage with only 422 showing some evidence of expression (>0 FPKM) and 137 with FPKM ≥1. Manual inspection of these revealed that most of these unannotated *A. pisum* proteins were aligned across several scaffolds of the *M. persicae* G006 assembly. These may represent gene fragments or pseudogenes in the *M. persicae* assembly, or they may be located within the introns of annotated genes and provide an example of the difficulty of gene predictors correctly resolving nested gene structures. We assessed the quality of gene predictions using Full-lengther Next [26] revealing that 74% of G006 and 70% of clone O transcript models were classified as complete (Figure 7). Additionally, a high coherence in gene length was found between *M. persicae* and *A. pisum* proteins (Figure 8). Taken together, these findings indicate a robust set of gene predictions.

**Figure 6:** Coverage of the three gene sets (*M. persicae* clones G006 and O and *A. pisum* version 2.1b) against a library of TE related sequences via TransposonPSI (TransposonPSI hits with >=30% similarity). A higher number of TE related genes are identified in the *A. pisum* (2.1b) gene set (5,151 protein hits with >=30% similarity and >=50% coverage).

**Figure 7:** Assessment of transcript completeness for G006 and clone O gene sets. Transcripts were classified as full-length, 5’-end, 3’-end, internal, coding (ORF predicted but no blast support) and unknown (no blast support) using full_lengther_next (v 0.0.8), 74% of G006 and 70 % of clone O transcript models were identified as complete.

**Figure 8:** Coherence in gene length between *M. persicae* G006/clone O and *A. pisum* proteins. Blast analysis identified 1342 (G006) and 1341 (clone O) proteins that had reciprocal best hits to 1349 *A. pisum* proteins identified as single copy in > 90% of Arthropods (OrthoDB). A high coherence in gene length was found between *M. persicae* G006 and *A. pisum* proteins r > 0.95.

In addition to protein-coding genes, we also identified 125 microRNA (miRNA), 273 tRNA and 69 rRNA genes in the *M. persicae* genome. Of the predicted protein coding genes 82% (15,171) had matches in the non-redundant (NR) databases (excluding hits to *A. pisum* and *M. persicae*), 70% (12,900) generated hits to InterPro signatures, 57% (10,577) were assigned GO terms, 9% (1,616) were mapped to known pathways, 15% (2,699) had signal peptides and 22% (4,056) had transmembrane regions.

**References**

1. Kelley DR, Schatz MC, Salzberg SL. Quake: quality-aware detection and correction of sequencing errors. Genome Biol 2010, 11:R116.

2. Gnerre S, Maccallum I, Przybylski D, Ribeiro FJ, Burton JN, Walker BJ, Sharpe T, Hall G, Shea TP, Sykes S, Berlin AM, Aird D, Costello M, Daza R, Williams L, Nicol R, Gnirke A, Nusbaum C, Lander ES, Jaffe DB. High-quality draft assemblies of mammalian genomes from massively parallel sequence data. Proc Natl Acad Sci U S A 2011, 108:1513-1518.

3. Simpson JT, Wong K, Jackman SD, Schein JE, Jones SJ, Birol I. ABySS: a parallel assembler for short read sequence data. Genome Res 2009, 19:1117-1123.

4. Boetzer M, Henkel CV, Jansen HJ, Butler D, Pirovano W. Scaffolding pre-assembled contigs using SSPACE. Bioinformatics 2011, 27:578-579.

5. Chikhi R, Rizk G. Space-efficient and exact de Bruijn graph representation based on a Bloom filter. Algorithms Mol Biol 2013, 8:22.

6. Flutre T, Duprat E, Feuillet C, Quesneville H. Considering transposable element diversification in de novo annotation approaches. PLoS One 2011, 6:e16526.

7. Hoede C, Arnoux S, Moisset M, Chaumier T, Inizan O, Jamilloux V, Quesneville H. PASTEC: an automatic transposable element classification tool. PLoS One 2014, 9:e91929.

8. Quesneville H, Bergman CM, Andrieu O, Autard D, Nouaud D, Ashburner M, Anxolabehere D. Combined evidence annotation of transposable elements in genome sequences. PLoS Comput Biol 2005, 1:166-175.

9. Stanke M, Waack S. Gene prediction with a hidden Markov model and a new intron submodel. Bioinformatics 2003, 19 Suppl 2:ii215-225.

10.Slater GS, Birney E. Automated generation of heuristics for biological sequence comparison. BMC Bioinformatics 2005, 6:31.

11.Trapnell C, Pachter L, Salzberg SL. TopHat: discovering splice junctions with RNA-Seq. Bioinformatics 2009, 25:1105-1111.

12.Trapnell C, Williams BA, Pertea G, Mortazavi A, Kwan G, van Baren MJ, Salzberg SL, Wold BJ, Pachter L. Transcript assembly and quantification by RNA-Seq reveals unannotated transcripts and isoform switching during cell differentiation. Nat Biotechnol 2010, 28:511-515.

13. Grabherr MG1, Haas BJ, Yassour M, Levin JZ, Thompson DA, Amit I, Adiconis X, Fan L, Raychowdhury R, Zeng Q, Chen Z, Mauceli E, Hacohen N, Gnirke A, Rhind N, di Palma F, Birren BW, Nusbaum C, Lindblad-Toh K, Friedman N, Regev A. Full-length transcriptome assembly from RNA-Seq data without a reference genome. Nat Biotechnol 2011, 29:644-652.

14. Haas BJ, Delcher AL, Mount SM, Wortman JR, Smith RK Jr, Hannick LI, Maiti R, Ronning CM, Rusch DB, Town CD, Salzberg SL, White O. Improving the Arabidopsis genome annotation using maximal transcript alignment assemblies. Nucleic Acids Res 2003, 31:5654-5666.

15.Cantarel BL, Korf I, Robb SM, Parra G, Ross E, Moore B, Holt C, Sanchez Alvarado A, Yandell M. MAKER: an easy-to-use annotation pipeline designed for emerging model organism genomes. Genome Res 2008, 18:188-196.

16.Haas BJ, Salzberg SL, Zhu W, Pertea M, Allen JE, Orvis J, White O, Buell CR, Wortman JR. Automated eukaryotic gene structure annotation using EVidenceModeler and the program to assemble spliced alignments. Genome Biol 2008, 9:R7.

17.Lowe TM, Eddy SR: tRNAscan-SE. a program for improved detection of transfer RNA genes in genomic sequence. Nucleic Acids Res 1997, 25:955-964.

18.Lagesen K, Hallin P, Rodland EA, Staerfeldt HH, Rognes T, Ussery DW. RNAmmer: consistent and rapid annotation of ribosomal RNA genes. Nucleic Acids Res 2007, 35:3100-3108.

19.Lorenz R, Bernhart SH, Honer Zu Siederdissen C, Tafer H, Flamm C, Stadler PF, Hofacker IL. ViennaRNA Package 2.0. Algorithms Mol Biol 2011, 6:26.

20.Prufer K, Stenzel U, Dannemann M, Green RE, Lachmann M, Kelso J. PatMaN: rapid alignment of short sequences to large databases. Bioinformatics 2008, 24:1530-1531.

21.Stocks MB, Moxon S, Mapleson D, Woolfenden HC, Mohorianu I, Folkes L, Schwach F, Dalmay T, Moulton V. The UEA sRNA workbench: a suite of tools for analysing and visualizing next generation sequencing microRNA and small RNA datasets. Bioinformatics 2012, 28:2059-2061.

22.Parra G, Bradnam K, Korf I. CEGMA: a pipeline to accurately annotate core genes in eukaryotic genomes. Bioinformatics 2007, 23:1061-1067.

23.Maumus F, Fiston-Lavier AS, Quesneville H. Impact of transposable elements on insect genomes and biology. Current Opinion in Insect Science 2015, 7.

24.International Aphid Genomics C. Genome sequence of the pea aphid *Acyrthosiphon pisum*. PLoS Biol 2010, 8: e1000313.

25.Huerta-Cepas J, Marcet-Houben M, Pignatelli M, Moya A, Gabaldon T. The pea aphid phylome: a complete catalogue of evolutionary histories and arthropod orthology and paralogy relationships for *Acyrthosiphon pisum* genes. Insect Mol Biol 2010, 19 Suppl 2:13-21.

26.Lara AJ, Perez-Trabado G, Villalobos DP, Diaz-Moreno S, Canton FR, Claros MG. A web tool to discover full-length sequences - Full-Lengther. Innovations in Hybrid Intelligent Systems 2007, 44:361-368.
