# Supplementary material for: Rapid transcriptional plasticity of duplicated gene clusters enables a clonally reproducing aphid to colonise diverse plant species
Source: Genome Biol. 2017 Feb 13;18:27. doi: 10.1186/s13059-016-1145-3 (PMC5304397; doi:10.1186/s13059-016-1145-3)
Supplement: Additional file 3: — Supplementary Text: Annotation of metabolic processes and specific gene families. (DOCX 584 kb) [file 13059_2016_1145_MOESM3_ESM.docx]

**Additional File 3: Annotation of metabolic processes and specific gene families**

***Metabolism***


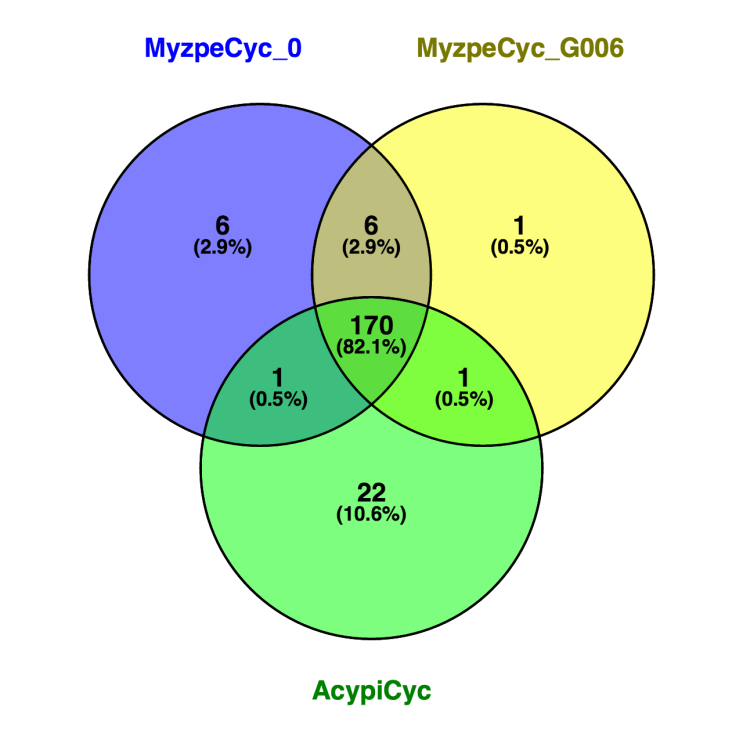
For a global metabolism annotation and a network reconstruction, the genomes of the two sequenced *M. persicae* clones were annotated using the CycADS annotation pipeline [1] followed by a metabolism reconstruction using Pathway Tools [2] generating MyzpeCyc databases for both *M. persicae* clones that were added to the ArthropodaCyc collection (<http://arthropodacyc.cycadsys.org/>) [Baa-Puyoulet et al., in press]. Differences between *M. persicae* clones O and G006 and Acyrthosiphon *pisum* are shown in Figure 1.

**Figure 1:** VENN diagram for amino acid metabolism EC enzymes of the two *M. persicae* clones compared to the pea aphid *A. pisum*.

***Cathepsin B***

Cathepsin B genes have been annotated for *A. pisum* [3] and are clustered in a single MCL gene family (family_110) with 2 additional, previously un-annotated, *A. pisum* genes bringing the total number of *A. pisum* cathepsin B genes to 30 (Additional File 23: Table S8). We identified 27 putative cathepsin B genes in *M. persicae* clone G006 (Additional File 24: Table S9). However, we initially identified 25 cathepsin B genes because pairs of MpCathB4 and MpCathB5 and of MpCathB10 and MpCathB11 were annotated as single genes in version 1.0 of the *M. persicae* clone G006 genome annotation. All 27 cathepsin B genes were also present in the genome of *M. persicae* clone O (Additional File 24: Table S9). We also identified cathepsin B genes in the genome of the plant feeding dipteran *Mayetiola destructor* (hessian fly) and 3 additional hemipteran species; *Diuraphis noxia* (Russian wheat aphid), *Diaphorina citri* (Asian citrus psyllid) and *Nilaparvata lugens* (brown planthopper). Details of the assembly and annotation versions used for these additional species are given in Table 1. Proteomes of these species were searched against a database of *M. persicae* capthepsin B sequences using blastp with an E value threshold of 5 x 10^-5^. Sequences less than 100 amino acids in length were considered incomplete and excluded from downstream analysis. In total we identified 17 putative cathepsin B genes in *D. noxia*, 8 in *D. citri*, 6 in *N. lugens* and 2 in *M. destructor* (Additional File 25: Table S10). The blast search also identified 13 genes annotated as being from cathepsin sub-families other than B in *D. citri*, two of which were partial sequences (<100aa). These were retained for phylogenetic analysis to aide rooting. All annotated cathepsin B sequences greater than 100 amino acids in length were aligned with muscle v. 3.8.31 [4] and their ML phylogeny estimated with FastTree [5] using the JTT model of protein evolution and CAT rate variation, branch support was assessed using Shimodaira-Hasegawa test (main text Figure 4). Domain analysis was conducted by InterPro (version 54) using cathepsin B protein sequences (Additional File 21: Figure S11).

**Table 1:** Additional genomes included for phylogenetic analysis of specific gene families.

| **Species** | **Common name** | **Assembly** | **Annotation** | **Reference** |
| --- | --- | --- | --- | --- |
| *Diaphorina citri* | Asian citrus psyllid | v1.1 (GCA_000475195.1) | NCBI annotation release 100 |  |
| *Diuraphis noxia* | Russian wheat aphid | v1.0 | v1.0 | [6] |
| *Nilaparvata lugens* | Brown planthopper | NilLug1.0 | Nlug_v1.1 | [7] |
| *Mayetiola destructor* | Hessian fly | Mdes_1.0 | OGS1.0 | [8] |

***Cuticular proteins***

Automatic annotation with CutProtFamPred

We conducted a comparative analysis of cuticular proteins found in 5 hemipteran genomes: *M. persicae* (clone G006), *A. pisum,* *D. noxia*, *D. citri*, *N. lugens and R. prolixus.* Cuticular proteins were identified and assigned to known cuticular protein families using CutProtFam-Pred [9], a web-based tool that identifies and classifies insect cuticular proteins based on profile Hidden Markov Models of characteristic conserved regions of each class of insect cuticular protein. Numbers of cuticular proteins identified in each genome are summarized in Additional File 26: Table S11A. In all five proteomes RR-2 cuticular proteins were most abundant with between 52 and 99 genes annotated in each genome. They also contained the highest number of genes differentially regulated in response to host change in *M. persicae* (Additional File 13: Figure S5A; Additional File 12: Table S5; Additional File 26: Table S11). To further investigate RR-2 cuticular protein evolution in Hemiptera we conducted a phylogenetic analysis of the annotated RR-2 genes. Given that RR-2 insect cuticular proteins tend to be highly diverged and difficult to align along their full length we conducted phylogenetic analysis using only the RR-2 domain. The location of the RR-2 domain in each annotated RR-2 protein was identified with a blastp search using an example RR-2 domain sequence (Additional File 26: Table S11C). The RR-2 domain of each sequence was then extracted based on the start and finish positions of the blastp hit and aligned with muscle v. 3.8.31 [4]. The alignment was manually inspected and 8 sequences with spuriously aligned RR-2 domains were removed, 3 from *M. persicae*, 2 from *A. pisum* and 3 from *R. prolixus* (Additional File 26: Table S11B). The curated RR-2 domain protein alignment was used to guide a codon alignment of the RR-2 domain with PAL2NAL [10]. ML phylogeny was estimated based on the codon alignment using FastTree [5] with the Jukes-Cantor nucleotide substitution model and CAT rate variation, branch support was assessed using Shimodaira-Hasegawa test (Additional File 13: Figure S5A). DE genes involved in host adjustment were then mapped onto the RR-2 domain phylogeny revealing that most DE genes belong to an aphid specific clade.

Manual annotation

In addition to the automatic annotation used in the comparative analysis of cuticular proteins across Hemiptera, we also conducted a more detailed manual annotation of cuticular proteins with R&R motif (defined as CPR; [11] for *M. persicae* clone G006, and confirmed with data sequences on clone O. To this aim, tBLASTn [12] searches were performed online against the Aphidbase database (<http://www.aphidbase.com/aphidbase/>), using the full DNA scaffolds set of *M. persicae* clone G006. Genes potentially coding for CPR, were identified using RR sub-groups consensus sequences based on the CuticleDB annotation tools (<http://biophysics.biol.uoa.gr/cuticleDB/>,[13]: GSYSYTxPDGxxYxVxYVAD-ENGFQPxGxHLP, EYDxxPxYxFxYxVxDxxTGDx KSQxExRxGDVVxGxYSLxExDGxxRTVxYTADxxNGFNAVVxxEx, V-xVxTxYH AQDxLGQxSFGHxxxxQxRxExxDAAGNKxGSYxYVDPxGKVxxxxYVA-AxGFR VAxx-NLPVxP corresponding to RR-1, RR-2 and RR-3 sub-groups, respectively. We used very loose criteria for parameters thresholds to detect all potential CPR: E-value threshold = 1, word size = 2 and BLOSUM 45. BlastN and tBLASTn searches using previously annotated CPR from *A. pisum* [14] as query sequences were also performed. Reciprocal BLAST searches were then performed to confirm that the *M. persicae* protein identified as the top hit to an *A. pisum* query identified the same *A. pisum* protein as the top hit when it was used as the query sequence. Manual annotation using *A. pisum* sequences, mRNA sequences (Aphidbase) as well as *M. persicae* ESTs data (C. Rispe, personal communication) was performed for retrieval of the full length coding sequence of each *M. persicae* CPR. Finally, to examine possible chimeras or errors due to a misassembling of *M. persicae* genome, BLAST searches against *M. persicae* clone O (genome sequence also available on Aphidbase) were performed using previously manually extracted CPR ORFs. Classification into the appropriate sub-group (for RR-1 and RR-2 proteins) was confirmed by the use of a profile hidden Markov model that discriminates between these two sub-groups [15]. All protein analysis such as predicted signal peptides or molecular weights estimation were performed using ExPASy tools (<http://www.expasy.org/tools/>) (Supplementary data not included).

As shown in the previous section “Automatic annotation with CutProtFamPred”, using this software on the released OGS v1.0 of *M. persicae* clone O and clone G006 allowed the detection of 13 RR-1 and 70 RR-2 unique genes. Using manual annotation on DNA scaffolds: 13, 63 and 2 unique genes harbouring respectively the RR-1, RR-2 and RR-3 motif were identified in *M. persicae* genome that constitute the final CPR set of this organism (Table 2). It is noteworthy that two genes previously detected as belonging to the RR-2 subfamily using CutProtFamPred software were classified as RR-3 genes when using sequence consensus (CuticleDB software; Table 2). In a similar way, in *A. pisum*, three CPR genes detected as RR-2 genes using CutProtFamPred software were identified as RR-3 by CuticleDB (Table 2). CPR genes subfamilies are located on different scaffolds showing a differentiated localization depending of the CPR nature (Table 3). Moreover, some scaffolds harbour several CPR genes as exemplified by scaffold_387 harbouring 18 RR-2 genes (Table 3). This presence of tandem repeats might reflect duplications events as suggested by phylogenetic analysis on RR2-proteins of *M. persicae* (Figure 2). Details of sequence IDs and phylome relationships between *M. persicae* clone G006 and clone O as well as *A. pisum* orthologs identified using PhylomeDB are available in Additional File 27: Table S12. Generally, protein sequences between automatic annotation of *M. persicae* genome were consistent with what we obtained by manually retrieval of ORFs. However, in some cases, assembling errors in the OGS could be detected and was further corrected. For example, 13 out of 63 RR-2 genes and one out of 12 for RR-1 genes were manually edited (supplementary data not included). This shows the importance of a manual annotation.

**Table 2:** Comparison of the automated and manual annotation of cuticular proteins within *M. persicae* and *A. pisum* genomes. CPFP stands for CutProtFam software detection.

|  | ***Myzus persicae* (Clone G006)** | |  | ***Acyrthosiphon pisum*** |  |  |
| --- | --- | --- | --- | --- | --- | --- |
|  | **consensus (cuticleDB)** | **CPFP** | **Edited** | **consensus (cuticleDB)*** | **CPFP** | **Edited** |
| RR-1 | 10 | 13 | 13 | 11 | 15 | 15 |
| RR-2 | 62 | 70 | 63 | 78 | 94 | 91 |
| RR-3 | 2 | - | 2 | 3 | - | 3 |
| CPAP1 | - | 6 | - | - | 10 |  |
| CPAP3 | - | 5 | - | - | 8 |  |
| CPCFC | - | 1 | - | - | 1 |  |
| CPF | - | 0 | - | - | 2 |  |
| Tweedle | - | 1 | - | - | 3 |  |

* from [14]

**Table 3:** Numbers of RR-1, RR-2, and RR-3 cuticular proteins found on *M. persicae* (clone G006) per scaffold.

| S**caffold name** | **Scaffold size (bp)** | **Family** | **Number** | **Total** |
| --- | --- | --- | --- | --- |
| scaffold_884 | 68365 | RR-1 | 1 |  |
| scaffold_517 | 211185 | RR-1 | 1 |  |
| scaffold_284 | 373139 | RR-1 | 2 |  |
| scaffold_246 | 402644 | RR-1 | 6 |  |
| scaffold_103 | 690745 | RR-1 | 1 |  |
| scaffold_86 | 782398 | RR-1 | 1 |  |
| scaffold_17 | 1364663 | RR-1 | 1 | **13** |
| scaffold_114 | 683074 | RR-2 | 3 |  |
| scaffold_116 | 663088 | RR-2 | 1 |  |
| scaffold_144 | 592465 | RR-2 | 1 |  |
| scaffold_183 | 493887 | RR-2 | 1 |  |
| scaffold_237 | 417588 | RR-2 | 1 |  |
| scaffold_244 | 414771 | RR-2 | 1 |  |
| scaffold_284 | 373139 | RR-2 | 1 |  |
| scaffold_319 | 330313 | RR-2 | 2 |  |
| scaffold_32 | 1063897 | RR-2 | 1 |  |
| scaffold_329 | 3320066 | RR-2 | 1 |  |
| scaffold_387 | 283906 | RR-2 | 18 |  |
| scaffold_397 | 279741 | RR-2 | 1 |  |
| scaffold_42 | 975233 | RR-2 | 2 |  |
| scaffold_511 | 213940 | RR-2 | 1 |  |
| scaffold_571 | 187496 | RR-2 | 1 |  |
| scaffold_58 | 857943 | RR-2 | 3 |  |
| scaffold_6 | 1836577 | RR-2 | 1 |  |
| scaffold_624 | 163939 | RR-2 | 1 |  |
| scaffold_634 | 159743 | RR-2 | 1 |  |
| scaffold_64 | 844835 | RR-2 | 1 |  |
| scaffold_678 | 136477 | RR-2 | 10 |  |
| scaffold_69 | 817146 | RR-2 | 7 |  |
| scaffold_7 | 1808486 | RR-2 | 1 |  |
| scaffold_75 | 804647 | RR-2 | 1 |  |
| scaffold_757 | 110610 | RR-2 | 1 | **63** |
| scaffold_155 | 560901 | RR-3 | 2 | **2** |

Phylogenetic analysis was performed using the corresponding protein sequence of RR-1 and RR-2 genes of *A. pisum* (automatic annotation) and *M. persicae* (manual annotation). RR-1 and RR-2 sub-groups were treated separately; the full RR-1 sequence protein was used in phylogenetic analyses while only the extended domain of 69 aa of each RR-2 protein, after alignment using Clustal Omega [16] and extraction, was used for further phylogenetic analyses. Three *A. pisum* RR-2 genes that did not correctly align where removed from the RR-2 analysis: ACYPI007858, ACYPI009701, and ACYPI086044. Phylogenetic relationships between *A. pisum* and *M.* *persicae* CPR were then assessed using the Phylogeny.fr platform [17]; sequences were aligned with MUSCLE (v. 3.8.31) [4] configured for highest accuracy (MUSCLE with default settings). In the case of RR-1 full protein analyses, after alignment, ambiguous regions (i.e. containing gaps and / or poorly aligned) were removed with Gblocks (v0.91b) using the following parameters: minimum length of a block after gap cleaning: 10, no gap positions were allowed in the final alignment and all segments with contiguous non conserved positions bigger than 8 were rejected, minimum number of sequences for a flank position: 85%. Then, phylogenetic trees were reconstructed using the maximum likelihood method implemented in the PhyML program (v3.1/3.0 aLRT). The WAG substitution model was selected assuming an estimated proportion of invariant sites (of 0.009) and 4 gamma-distributed rate categories to account for rate heterogeneity across sites. The gamma shape parameter was estimated directly from the data (gamma=3.517). Reliability for internal branch was assessed using the aLRT test (SH-Like). Graphical representation and edition of the phylogenetic tree were performed with TREEDYN (v. 198.3; [18] (Figures 2, 3, 4).

**Figure 2:** Phylogenetic relationships of the core RR-2 proteins of *M. persicae.* Phylogenetic reconstruction was performed using the extended domain of 69 aa specific of RR2-proteins on the full set of manually annotated RR-2 proteins of *M. persicae* as described in Supplementary data. Putative RR-2 proteins found on scaffold_387 are highlighted. Numbers at nodes indicates the percentage of 1000 bootstrap replicates that support the node. The scale represents probabilities of change from one amino acid to another in terms of a unit, which is an expected 1% change between two amino acid sequences.

**Figure 3:** Phylogenetic relationships of full RR-1 proteins of *A.* *pisum* and *M. persicae*. Phylogenetic reconstruction was performed using an updated list of RR-1 proteins of *A. pisum* (automatic annotation using CutProtFamPred) and the full set of manually annotated RR-1 proteins of *M. persicae* as described in Supplementary data. Number at nodes indicate the percentage of 1000 bootstrap replicates that support the node. The scale represents probabilities of change from one amino acid to another in terms of a unit, which is an expected 1% change between two amino acid sequences.

**Figure 4:** Phylogenetic relationships of the core RR-2 proteins of *A.* *pisum* and *M. persicae.* Phylogenetic reconstruction was performed using the extended domain of 69 aa specific of RR2-proteins on the updated list of RR-2 proteins of *A. pisum* (automatic annotation using CutProtFamPred) and the full set of manually annotated RR-2 proteins of *M. persicae* as described in Supplementary data. Number at nodes indicate the percentage of 1000 bootstrap replicates that support the node. The scale represents probabilities of change from one amino acid to another in terms of a unit, which is an expected 1% change between two amino acid sequences.

***Cytochrome P450s***

*M. persicae* P450 genes were annotated based on BLASTP similarity to annotated *A. pisum* P450s and presence of the PFAM p450 domain (PF00067). All annotated *A. pisum* cytochrome P450 sequences were downloaded from the P450 website (67 in total) [19] and used as a database against which *M. persicae* proteins were queried with BLASTP. *M. persicae* proteins that matched an *A. pisum* P450 with a minimum E value of 5 x 10^-5^ were considered candidate P450 sequences and classified into P450 clans based on their best BLASTP hit. In total 68 *M. persicae* p450s were identified, all of which contained the PF00067 domain. Protein sequences of *A. pisum* and *M. persicae* P450s were then aligned with muscle v. 3.8.31 [4] and their phylogeny estimated with RAxML v. 8.0.23 [20] using automatic protein model selection and gamma distributed rate variation. Branch support was estimated based on 100 rapid bootstrap replicates drawn onto the best scoring ML tree. 8 *M. persicae* P450 sequences were excluded from the phylogenetic analysis as they either represented gene fragments or had incorrect annotations (Additional File 15: Figure S7; Additional File 28: Table S13).

***Lipases***

MCL family 16 was highlighted in the differential expression analysis of aphids reared on different host plants as having multiple members differentially expressed (Additional File 12: Table S5). Inspection of the *M. persicae* automated blast2GO and interproscan annotation revealed these genes to be lipases. Protein sequences from *M. persicae*, *A. pisum*, *R. prolixus* and *D. melanogaster* were extracted from MCL family 16 for phlyogentic analysis. The sequences were aligned with muscle v. 3.8.31 [4] and their phylogeny estimated with RAxML v. 8.0.23 [20] using automatic protein model selection and gamma distributed rate variation. Branch support was estimated based on 100 rapid bootstrap replicates drawn onto the best scoring ML tree (Additional File 16: Figure S8). Manual inspection of the family 16 alignment revealed all sequences to align well over their full length no evidence of fragmented sequences or misannotation. Lipase sequences included in the phylogenetic analysis are summarised in Additional File 29: Table S14.

***UDP-glucosyltransferase (UGT)***

All UGT transcript IDs for *D. melanogaster* were downloaded from FlyBase and used to search MCL gene families. All *D. melanogaster* UGT genes clustered into a single family which included sequences from other insect species included in the comparative analysis of gene families. Based on the MCL clustering results 57 UGT genes were identified in *M. persicae*, 59 in *A. pisum* and 13 in *R. prolixus*. Identified *M. persicae*, *A. pisum*, *R. prolixus* and *D. melanogaster* UGT protein sequences were extracted for phylogentic analysis. The sequences were aligned with muscle v. 3.8.31 [4] and their phylogeny estimated with RAxML v. 8.0.23 [20] using automatic protein model selection and gamma distributed rate variation. Branch support was estimated based on 100 rapid bootstrap replicates drawn onto the best scoring ML tree (Additional File 14: Figure S6). Sequences included in the phylogenetic analysis are summarized in Additional File 30: Table S15.

Reference

1. Vellozo AF, Véron AS, Baa-Puyoulet P, Huerta-Cepas J, Cottret L, Febvay G, Calevro F, Rahbé Y, Douglas AE, Gabaldón T, Sagot MF, Charles H, Colella S. CycADS: an annotation database system to ease the development and update of BioCyc databases. Database (Oxford) 2011, 2011:bar008.
2. Karp PD, Paley SM, Krummenacker M, Latendresse M, Dale JM, Lee TJ, Kaipa P, Gilham F, Spaulding A, Popescu L, Keseler IM, Caspi R. Pathway Tools version 13.0: integrated software for pathway/genome informatics and systems biology. Brief Bioinform 2010, 11:40-79.
3. Rispe C, Kutsukake M, Doublet V, Hudaverdian S, Legeai F, Simon JC, Tagu D, Fukatsu T. Large gene family expansion and variable selective pressures for cathepsin B in aphids. Mol Biol Evol 2008, 25:5-17.
4. Edgar RC. MUSCLE: multiple sequence alignment with high accuracy and high throughput. Nucleic Acids Res 2004, 32:1792-1797.
5. Price MN, Dehal PS, Arkin AP. FastTree 2-approximately maximum-likelihood trees for large alignments. PLoS One 2010, 5:e9490.
6. Nickerson ML, Dean M, Song Y, Hoyt PR, Rhee H, Kim C, Puterka GJ.The genome of *Diuraphis noxia*, a global aphid pest of small grains. BMC Genomics 2015, 16: 429.
7. Xue J, Zhou X, Zhang CX, Yu LL, Fan HW, Wang Z, Xu HJ, Xi Y, Zhu ZR, Zhou WW, Pan PL, Li BL, Colbourne JK, Noda H, Suetsugu Y, Kobayashi T, Zheng Y, Liu S, Zhang R, Liu Y, Luo YD, Fang DM, Chen Y, Zhan DL, Lv XD, Cai Y, Wang ZB, Huang HJ, Cheng RL, Zhang XC, Lou YH, Yu B, Zhuo JC, Ye YX, Zhang WQ, Shen ZC, Yang HM, Wang J, Wang J, Bao YY, Cheng JA. Genomes of the rice pest brown planthopper and its endosymbionts reveal complex complementary contributions for host adaptation. Genome Biol 2014, 15: 521.
8. Zhao C, Escalante LN, Chen H, Benatti TR, Qu J, Chellapilla S, Waterhouse RM, Wheeler D, Andersson MN, Bao R, Batterton M, Behura SK, Blankenburg KP, Caragea D, Carolan JC, Coyle M, El-Bouhssini M, Francisco L, Friedrich M, Gill N, Grace T, Grimmelikhuijzen CJ, Han Y, Hauser F, Herndon N, Holder M, Ioannidis P, Jackson L, Javaid M, Jhangiani SN, Johnson AJ, Kalra D, Korchina V, Kovar CL, Lara F, Lee SL, Liu X, Löfstedt C, Mata R, Mathew T, Muzny DM, Nagar S, Nazareth LV, Okwuonu G, Ongeri F, Perales L, Peterson BF, Pu LL, Robertson HM, Schemerhorn BJ, Scherer SE, Shreve JT, Simmons D, Subramanyam S, Thornton RL, Xue K, Weissenberger GM, Williams CE, Worley KC, Zhu D, Zhu Y, Harris MO, Shukle RH, Werren JH, Zdobnov EM, Chen MS, Brown SJ, Stuart JJ, Richards S. A massive expansion of effector genes underlies gall-formation in the wheat pest *Mayetiola destructor*. Curr Biol 2015, 25: 613-620.
9. Ioannidou ZS, Theodoropoulou MC, Papandreou NC, Willis JH, Hamodrakas SJ: CutProtFam-Pred: detection and classification of putative structural cuticular proteins from sequence alone, based on profile hidden Markov models. Insect Biochem Mol Biol 2014, 52:51-59.
10. Suyama M, Torrents D, Bork P. PAL2NAL: robust conversion of protein sequence alignments into the corresponding codon alignments. Nucleic Acids Res 2006, 34:W609-W612.
11. Rebers JE, Riddiford LM. Structure and expression of a *Manduca sexta* larval cuticle gene homologous to *Drosophila* cuticle genes. J Mol Biol 1988, 203:411-423.
12. Altschul SF, Madden TL, Schäffer AA, Zhang J, Zhang Z, Miller W, Lipman DJ: Gapped BLAST and PSI-BLAST: a new generation of protein database search programs. Nucleic Acids Res 1997, 25:3389-3402.
13. Willis, J. H., Iconomidou, V. A., Smith R. F., and Hamodrakas S. J. Cuticular proteins. In: Gilbert, L., Iatrou, K., Gill, S.S. (Eds. Elsevier Pergamon, Oxford), Comprehensive Molecular Insect Science 2005,4, 30.
14. Gallot A, Rispe C, Leterme N, Gauthier JP, Jaubert-Possamai S, Tagu D: Cuticular proteins and seasonal photoperiodism in aphids. Insect Biochem Mol Biol 2010, 40:235-240.
15. Karouzou MV, Spyropoulos Y, Iconomidou VA, Cornman RS, Hamodrakas SJ, Willis JH. *Drosophila* cuticular proteins with the R&R Consensus: annotation and classification with a new tool for discriminating RR-1 and RR-2 sequences. Insect Biochem Mol Biol 2007, 37:754-760.
16. Sievers F, Wilm A, Dineen D, Gibson TJ, Karplus K, Li W, Lopez R, McWilliam H, Remmert M, Söding J, Thompson JD, Higgins DG. Fast, scalable generation of high-quality protein multiple sequence alignments using Clustal Omega. Mol Syst Biol 2011, 7:539.
17. Dereeper A, Guignon V, Blanc G, Audic S, Buffet S, Chevenet F, Dufayard JF, Guindon S, Lefort V, Lescot M, Claverie JM, Gascuel O. Phylogeny.fr: robust phylogenetic analysis for the non-specialist. Nucleic Acids Res 2008, 36:W465-469.
18. Chevenet F: TreeDyn: towards dynamic graphics & annotations for trees analyses V194.3. [http://wwwtreedynorg/] 2006.
19. Nelson DR. The cytochrome p450 homepage. Hum Genomics 2009, 4:59-65.
20. Stamatakis A. RAxML version 8: a tool for phylogenetic analysis and post-analysis of large phylogenies. Bioinformatics 2014, 30:1312-1313.
