# Supplementary figures and images for: Rapid transcriptional plasticity of duplicated gene clusters enables a clonally reproducing aphid to colonise diverse plant species
Source: Genome Biol. 2017 Feb 13;18:27. doi: 10.1186/s13059-016-1145-3 (PMC5304397; doi:10.1186/s13059-016-1145-3)

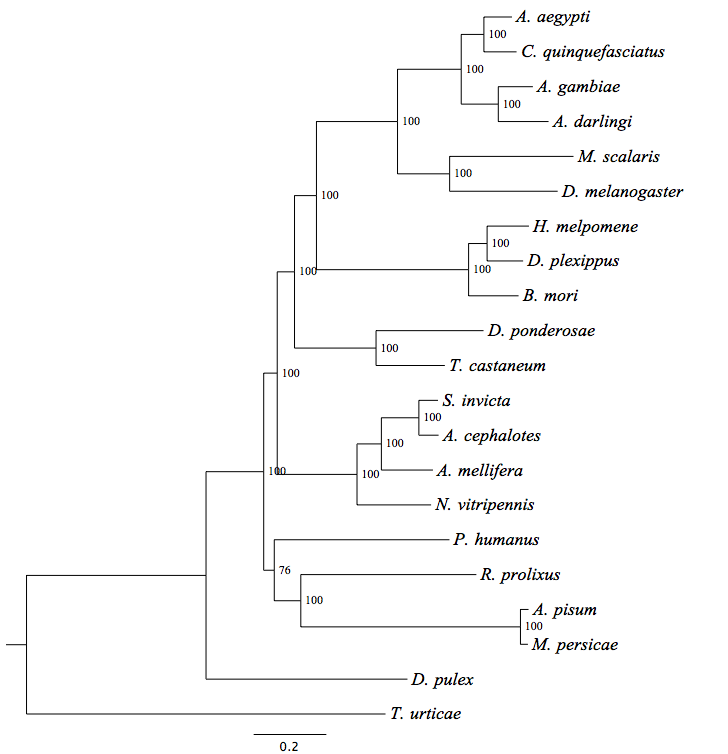

Supplement: Additional file 5: Figure S1. — ML phylogeny of 21 arthropod species with fully sequenced genomes based on 66 strictly conserved single-copy orthologs. Sequences were aligned with MUSCLE [76] and trimmed to remove poorly aligned regions with TrimAl [77]. The phylogeny was estimated using RAxML [30] with each gene treated as a separate partition. Automatic protein model selection was implemented in RAxML with gamma distributed rate variation. Values at nodes show bootstrap support based on 100 rapid bootstrap replicates carried out with RAxML. (PNG 44 kb) [file 13059_2016_1145_MOESM5_ESM.png]

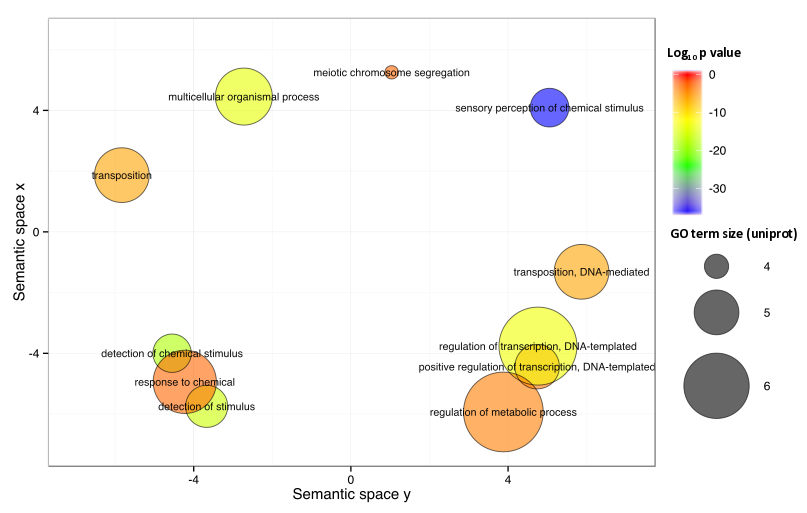

Supplement: Additional file 6: Figure S2. — Enriched GO terms relating to biological processes of aphid-specific M. persicae genes. GO term enrichment analysis was carried out using Fishers’ exact test in BINGO [93] with correction for multiple testing applied by the Benjamini–Hochberg procedure allowing for a 10% FDR. GO terms from aphid-specific genes were compared to GO terms from the complete set of M. persicae genes. Enriched GO terms were reduced and visualised with REVIGO [94]. GO terms are clustered by semantic similarity with the size of each circle relative to the size of the GO term in UniProt (larger circles = more general GO terms) and coloured by their p values according to Fisher’s exact test of enrichment. A complete list of enriched GO terms for aphid-specific genes are given in Additional file 7: Table S3. (PNG 76 kb) [file 13059_2016_1145_MOESM6_ESM.png]

A)

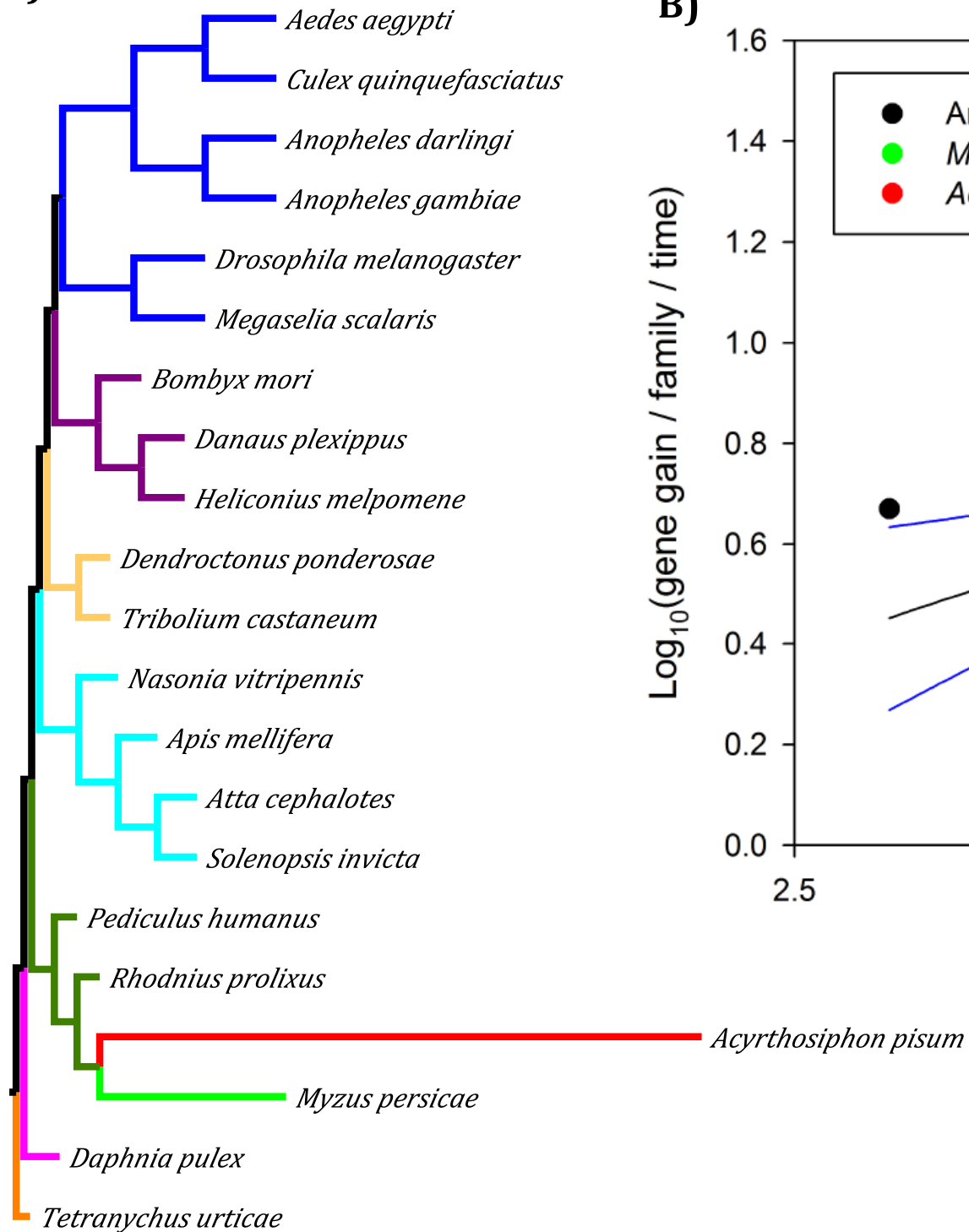

B)

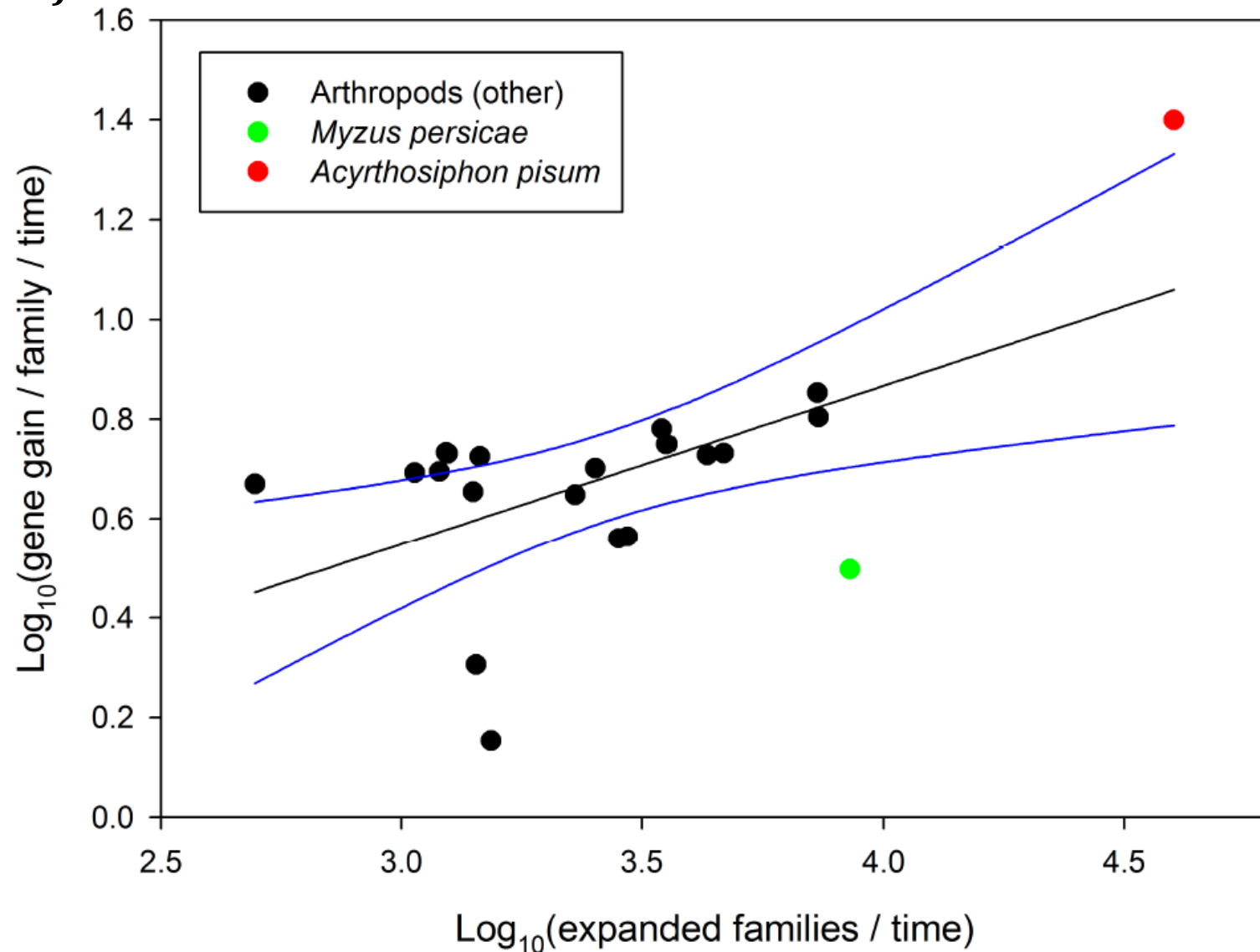

Supplement: Additional file 8: Figure S3. — Model based analysis of gene gain and loss across arthropods. Gene gain and loss (λ) was modelled across the arthropod phylogeny under a birth–death process with CAFE [78] for 4983 widespread gene families inferred to be present in the most recent common ancestor (MRCA) of all included taxa. Nested models with increasing numbers of lambda parameters were compared using likelihood ratio test (Additional file 32 and 33: Tables S17 and S18). Results are shown for the best fitting clade-specific rates model. A Arthropod phylogeny scaled by clade-specific ML values of λ inferred by CAFE. Branch colours indicate where separate λ parameters were specified. A. pisum (red) has undergone a significant increase in the rate of gene gain and loss (λ) compared to other arthropod species. B Linear regression line (mean and 5–95% confidence intervals) of the number of expanded families versus the gain in gene number per family across arthropod taxa. Both the size of the expansion and the number of expanded families were log10 transformed and scaled relative to the divergence time. There is a significant positive relationship across arthropod taxa in the number of families that expand and the mean number of genes gained within the expanded families (Regression: R2 = 29.4%; F1,19 = 9.32, p = 0.007). The specialist aphid A. pisum (red) is an outlier, showing a relative excess in both the number of expanded families and the magnitude of the mean family expansion. In contrast, although the generalist aphid M. persicae (green) has many expanded families, it shows relatively little gene gain per family. (PDF 215 kb) [file 13059_2016_1145_MOESM8_ESM.pdf]

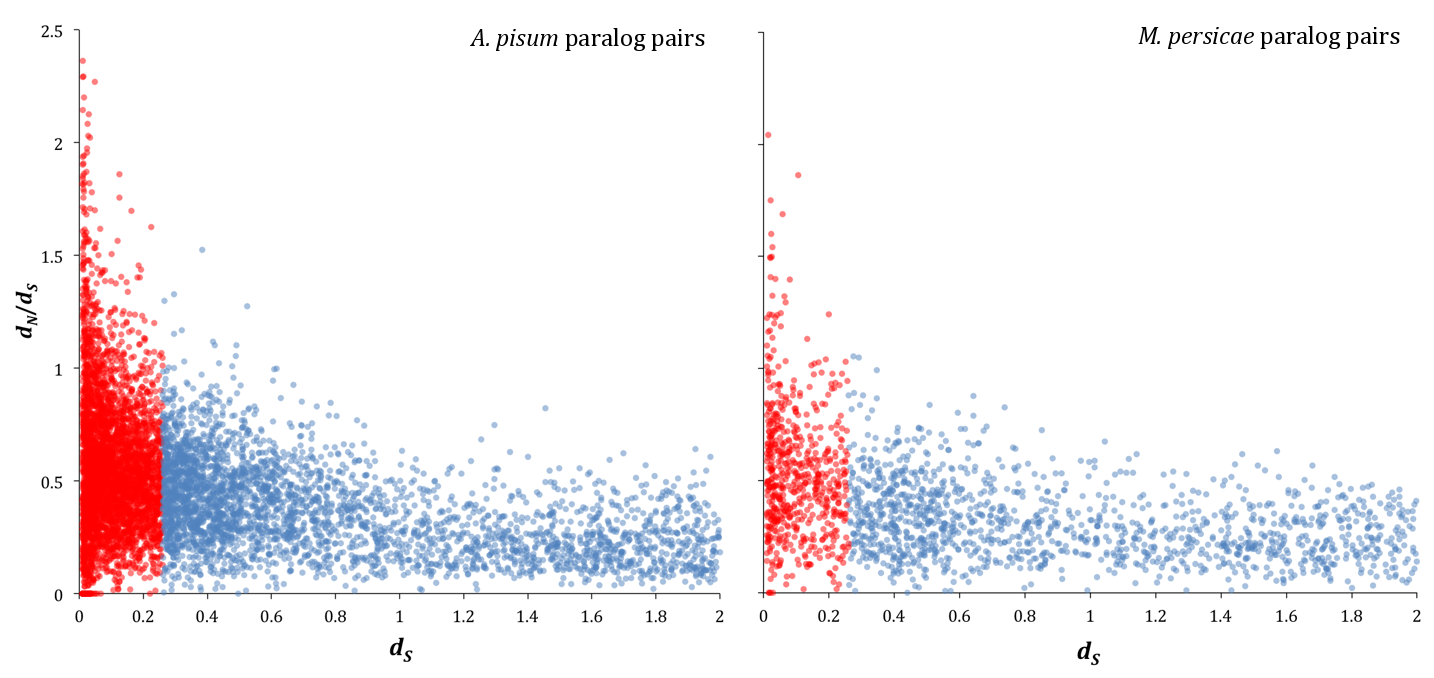

Supplement: Additional file 10: Figure S4. — The rate of evolution (d N /d S) vs. time since duplication (d S) for A. pisum and M. persicae paralog pairs. Paralog pairs that duplicated before the divergence of A. pisum and M. persicae (d S > 0.26) are coloured blue, paralog pairs that duplicated after the divergence of A. pisum and M. persicae (d S < 0.26) are coloured red. (PNG 339 kb) [file 13059_2016_1145_MOESM10_ESM.png]

A

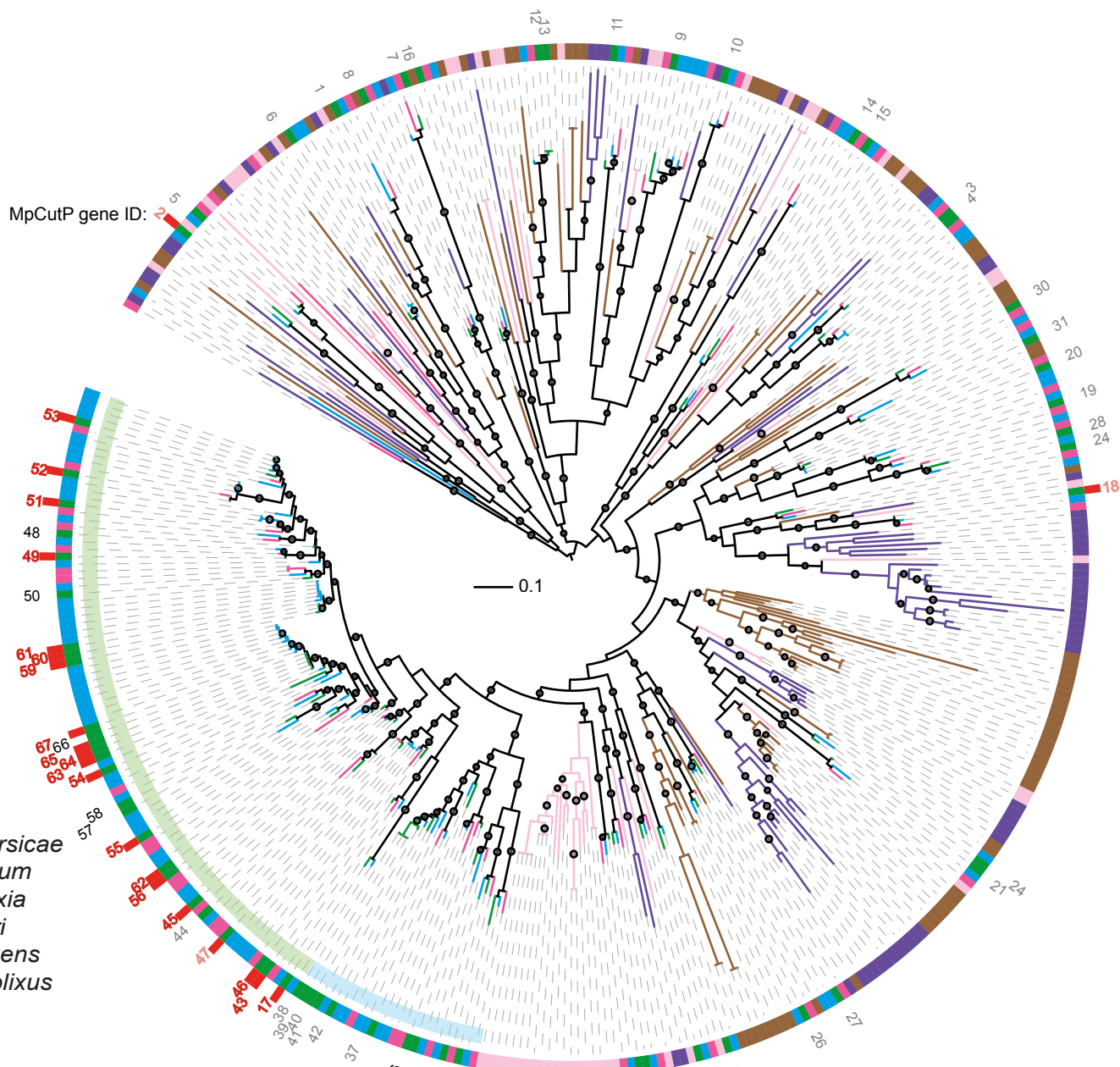

B

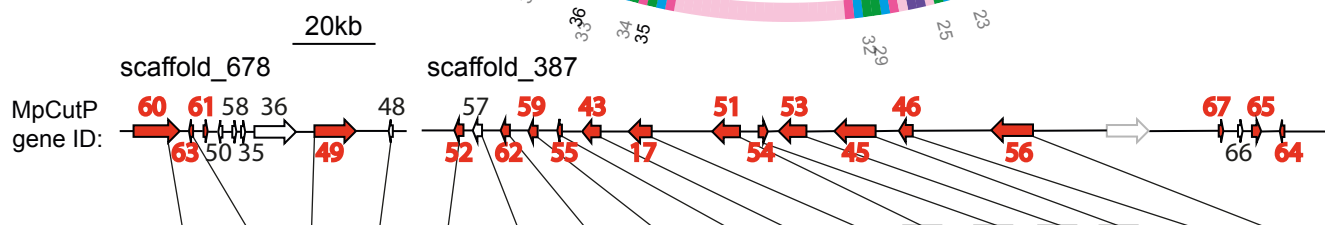

C

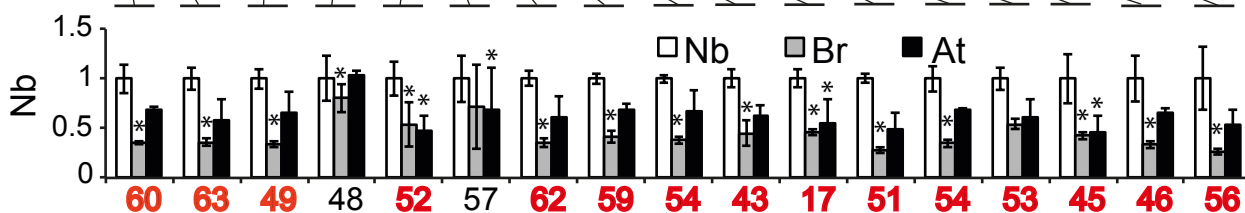

D

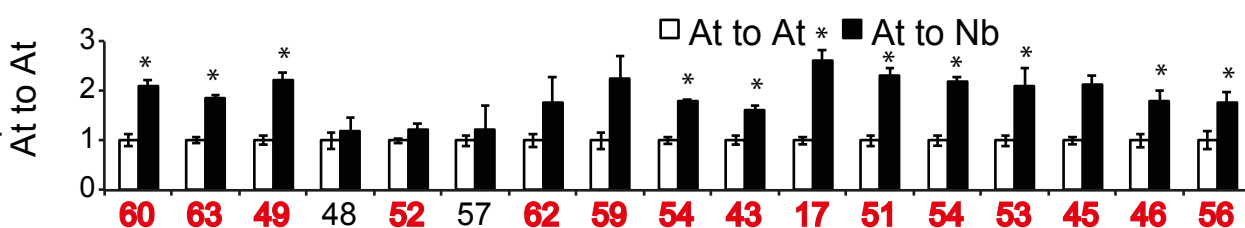

E

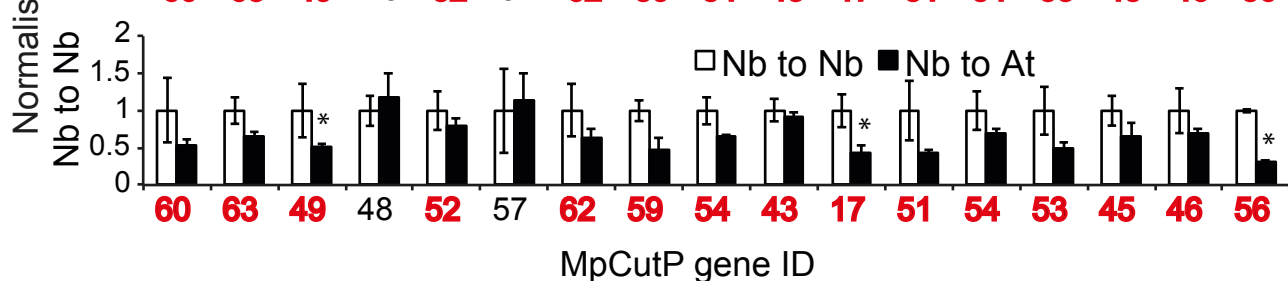

Supplement: Additional file 13: Figure S5. — The Rebers and Riddiford subgroup 2 (RR-2) cuticular protein genes that are differentially expressed upon M. persicae host change belong predominantly to a single aphid-expanded clade and form gene clusters in the M. persicae genome. A ML phylogenic tree of arthropod RR-2 cuticular protein–protein sequences. The sequences were aligned with Muscle [76] and the phylogeny estimated using FastTree [92] (JTT + CAT rate variation). Circles on branches indicate SH-like local support values >80%, scale bar below indicates 0.1 substitutions per site. Rings from outside to inside: ring 1, M. persicae RR-2 cuticular protein (MpCutP) gene identities (IDs) with numbers in red indicating upregulation of these genes in M. persicae reared for one year on N. benthamiana relative to those reared for one year on B. rapa, and bold font indicates location on the RR-2 cuticular protein multigene clusters shown in (B); ring 2, red squares indicate MpCutP genes that are differentially expressed upon M. persicae host change; ring 3, CutP genes from different arthropods following the colour scheme of the legend in the lower left corner and matching the colours of the branches of the phylogenetic tree; ring 4, aphid-expanded (AE) clades with AE_Clade I labelled light green and AE_Clade II light blue. B MpCutP multigene clusters of the M. persicae genome. Lines indicate the genomic scaffolds on which the MpCutP genes are indicated with block arrows. Gene IDs above the genes match those of the phylogenetic tree in A, with block arrows and fonts highlighted in red being DE upon host change. Scale bar on right shows 20 kb. C Relative expression levels of MpCutP genes of M. persicae at seven weeks being reared on N. benthamiana (Nb), B. rapa (Br) and A. thaliana (At). Numbers under the graphs indicate MpCutP gene IDs with those in red font differentially expressed as in (A). Batches of five adult females were harvested for RNA extraction and qRT-PCR assays. Bars represent expression values (mean ± [file 13059_2016_1145_MOESM13_ESM.pdf]

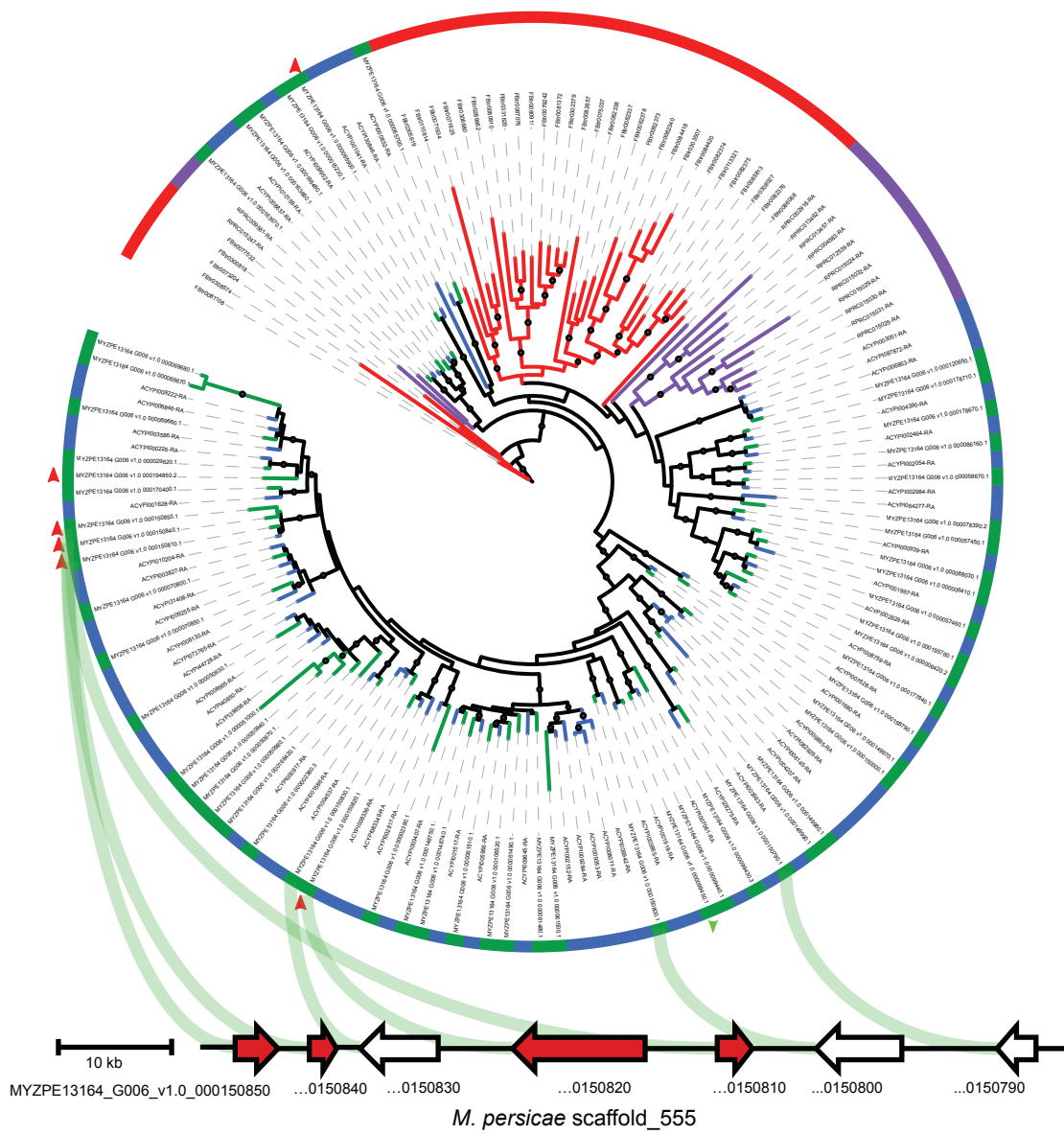

Supplement: Additional file 14: Figure S6. — UDP-glucosyltransferase (UGT) genes that show differential expression on different host plants fall within an aphid-specific clade and some are associated with an array of tandem duplicates. Phylogeny of (UGT) proteins from M. persicae (green), A. pisum (blue), R. prolixus (purple) and D. melanogaster (red). Protein sequences were aligned with Muscle [76] and the phylogeny estimated using RAxML [30] with automatic model selection and gamma distributed rate variation. One hundred rapid bootstrap replicates were carried out with RAxML. Grey circles on branches indicate bootstrap support greater than 80%. Genes showing elevated expression in aphid reared on B. rapa are indicated in red. Bottom: part of scaffold_555 containing seven predicted UGT genes, four of which are more highly expressed on B. rapa host plants. Scale bar at left is 10 kb. (PDF 978 kb) [file 13059_2016_1145_MOESM14_ESM.pdf]

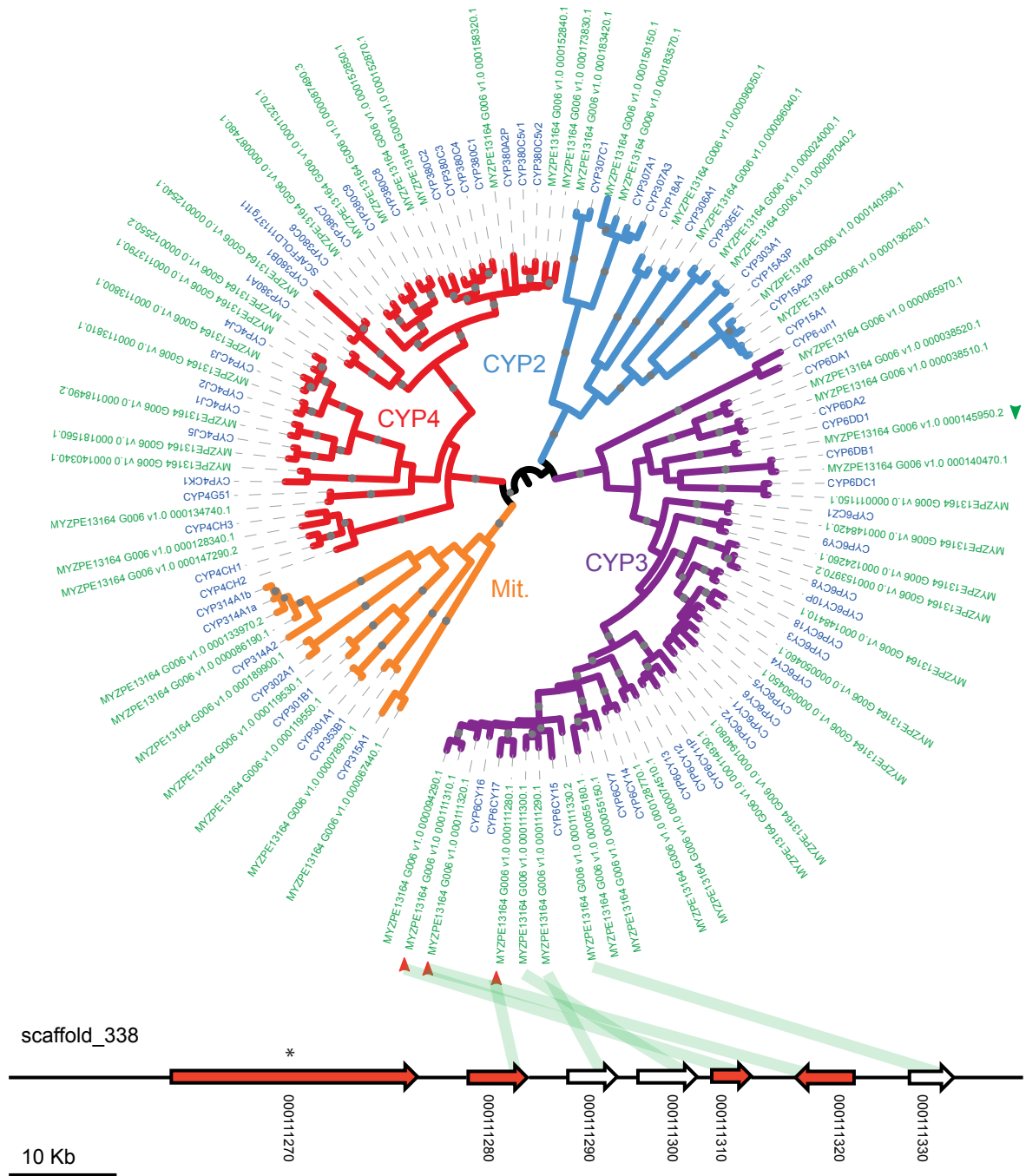

Supplement: Additional file 15: Figure S7. — ML phylogeny of Cytochrome-P450 proteins from M. persicae (green) and A. pisum (blue). A. pisum P450s are named according to their annotation from the Cytochrome-P450 homepage [95]. Protein sequences were aligned with Muscle [76] and the phylogeny estimated using RAxML [30] with automatic model selection and gamma distributed rate variation. One hundred rapid bootstrap replicates were carried out with RAxML. Grey circles on branches indicate bootstrap support greater than 80%. Transcripts that show elevated expression on B. rapa are indicated with red arrowhead and on N. benthamiana with a green arrowhead. Bottom: scaffold 338 containing four differentially expressed cytochrome-P450 genes (red), together with three non-regulated p450s (white) is shown. (*locus 000111270 is one of eight M. persicae P450 genes that were excluded from phylogenetic analysis after manual curation as they were either fragmented or had incorrect annotations. (PDF 885 kb) [file 13059_2016_1145_MOESM15_ESM.pdf]

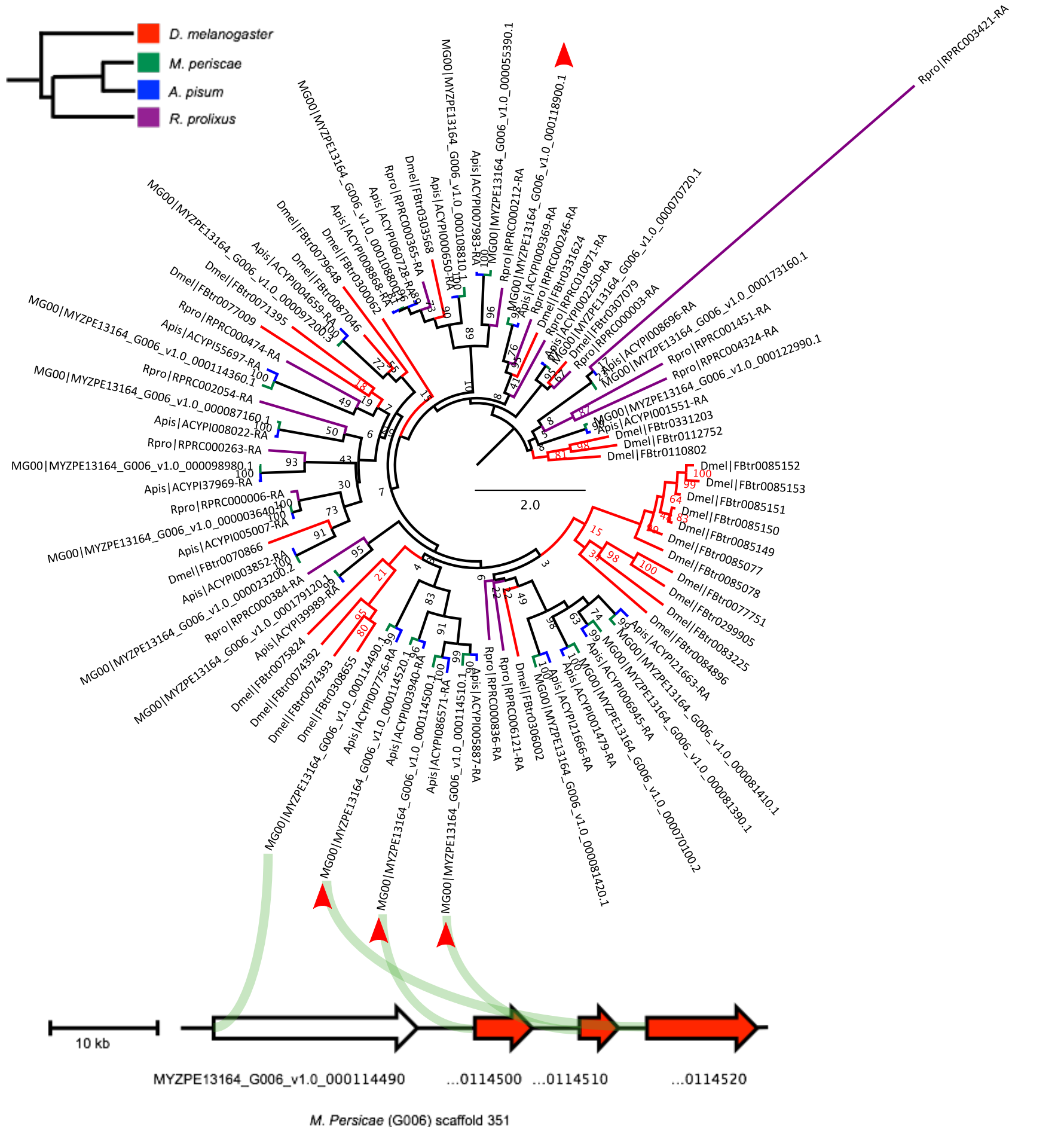

Supplement: Additional file 16: Figure S8. — ML phylogeny of M. persicae, A. pisum and D. melanogaster lipase-like genes (MCL family 16). Protein sequences were aligned with Muscle [76] and the phylogeny estimated using RAxML [30] with automatic model selection and gamma distributed rate variation. Five hundred rapid bootstrap replicates were carried out with RAxML, bootstrap support values are shown at nodes. Genes showing significantly elevated expression on B. rapa are indicated with red arrows. Bottom: part of scaffold 351 which contains four lipase-like genes in a tandem array, three of which are upregulated in aphids reared on B. rapa. (PDF 1296 kb) [file 13059_2016_1145_MOESM16_ESM.pdf]

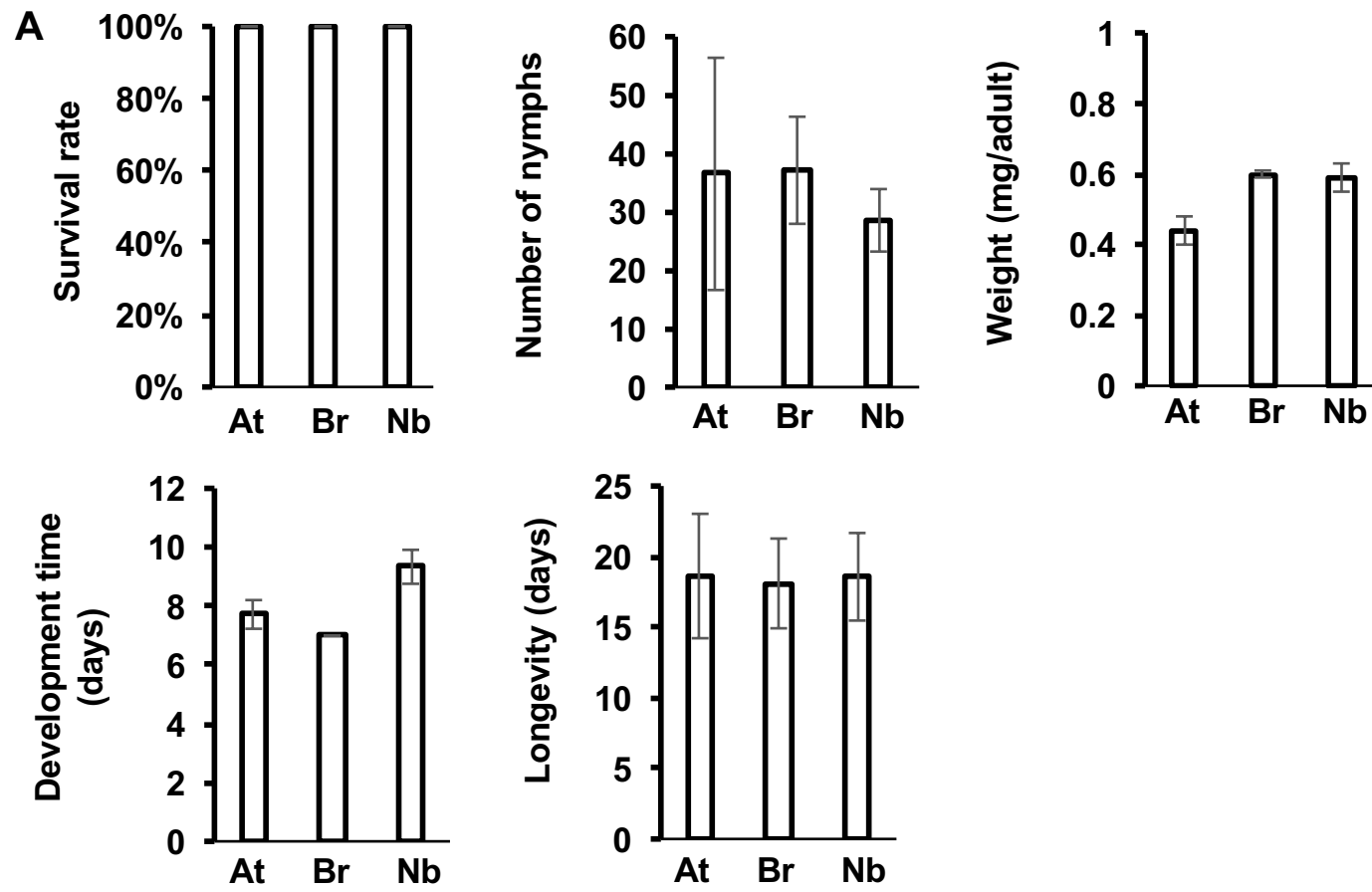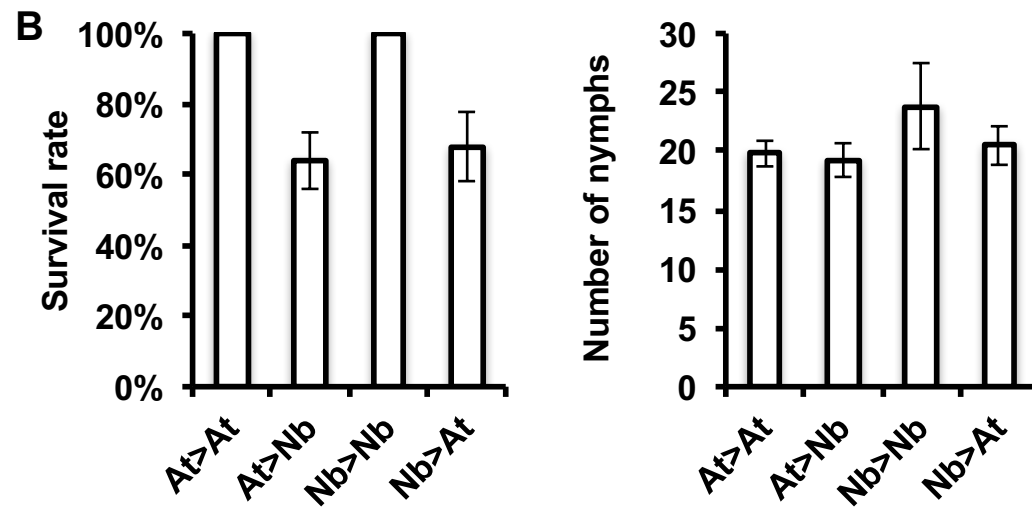

Supplement: Additional file 18: Figure S9. — Performance of M. persicae clone O on three plant species. A M. persicae clone O performance at about seven weeks after the plant host switch from Brassica rapa (Br) to Arabidopsis thaliana (At) or Nicotiana benthamiana (Nb) as indicated on the x-axes. Seven to 20 one-day-old nymphs were transferred to the same plant hosts and survival rates (%) were assessed on the fifth day when these aphids became adults. To measure reproduction rates, the progeny of five aphids at 7, 9 and 11 days old were counted. The graphs show the total number of nymphs counts for the three days. The weights (mg/adult) are the average from 10 one-day-old adults. The development time is the average number of days between births of 10 nymphs and emergence of adults from these nymphs. The longevity is the average number of days between births and deaths of 10 aphids starting from one-day-old nymphs. Columns represent values (mean ± SD) from the 3–5 technical replicates (p >0.05). Experiments were repeated two to three times with similar results. B M. persicae clone O performance within two days upon a host switch. Ten third instar nymphs from a colony reared for more than one year on A. thaliana were transferred from A. thaliana to A. thaliana (At > At) or to N. benthamiana (At > Nb). In addition, 10 third instar nymphs from a colony reared for more than one year on N. benthamiana were transferred from N. benthamiana to N. benthamiana (Nb > Nb) or to A. thaliana (Nb > At). Survival rates (out of 10 nymphs) were assessed two days later, and the reproduction rates of the surviving aphids were assessed as in (A). Columns represent values (mean ± SD) from five technical replicates (p >0.05). Experiments were repeated two times with similar results. (PDF 87 kb) [file 13059_2016_1145_MOESM18_ESM.pdf]

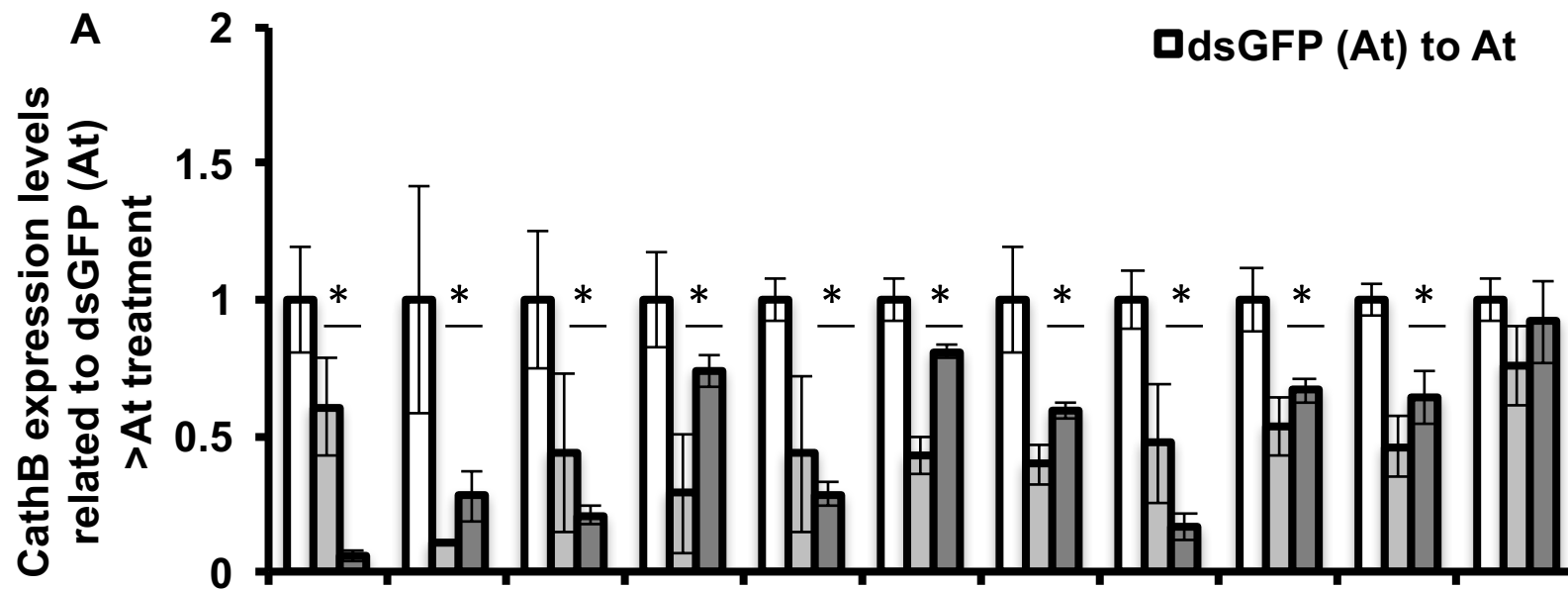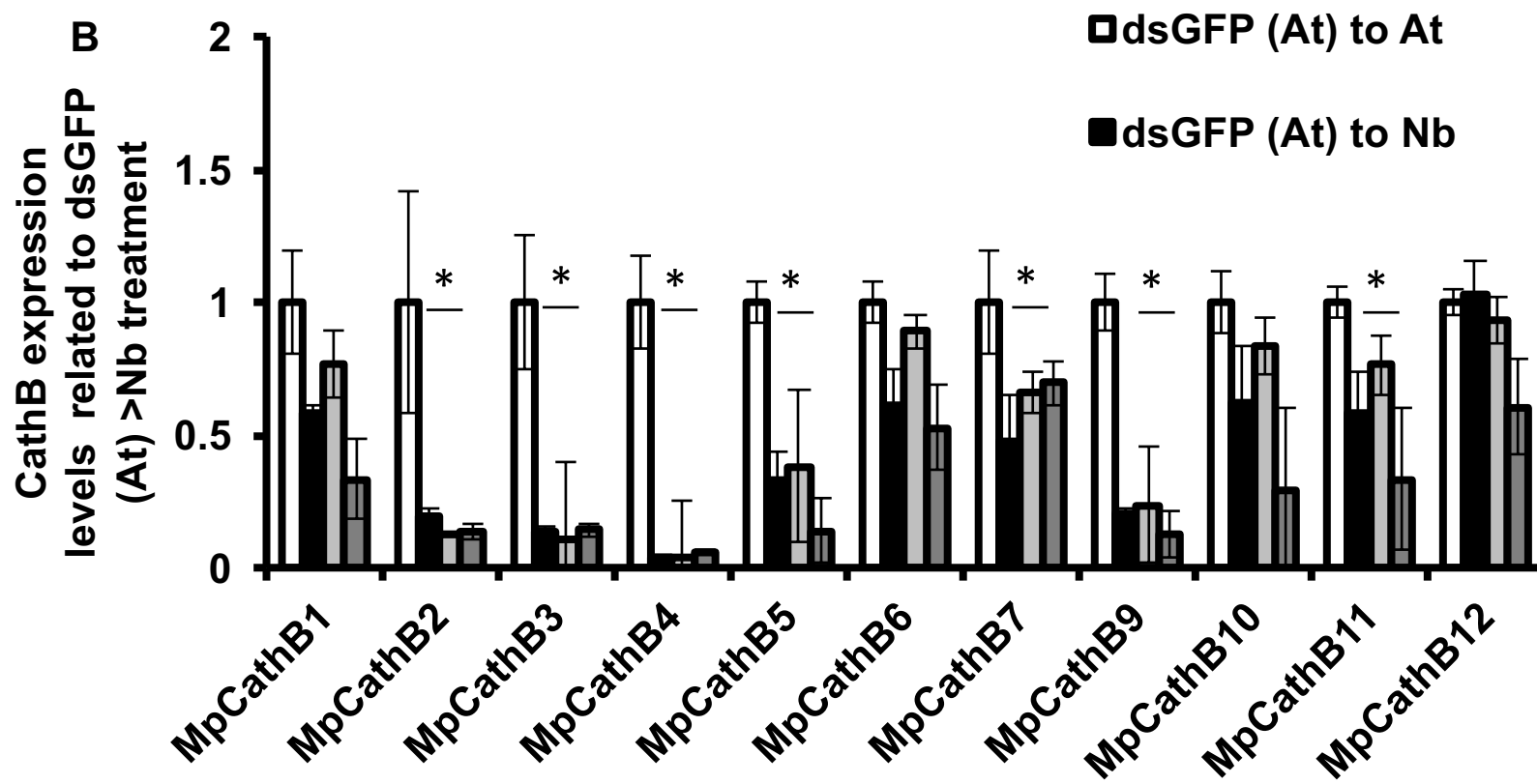

Supplement: Additional file 20: Figure S10. — Cathepsin B expression levels of CathB-RNAi and control (dsGFP-exposed) aphids after two days on non-transgenic A. thaliana and N. benthamiana plants. Ten third instar nymphs on dsCathB (lines 17-5 and 18-2) and dsGFP transgenic A. thaliana lines were transferred to non-transgenic A. thaliana (At) (A) and non-transgenic N. benthamiana (Nb) (B) plants. Aphids were harvested two days later for RNA extraction and qRT-PCR analyses. Bars represent mean ± SD of the relative M. persicae CathB expression levels (compared to aphids on dsGFP (control) plants) of three independent biological replicates with five adult females each. *p <0.05. (PDF 170 kb) [file 13059_2016_1145_MOESM20_ESM.pdf]
