# Supplementary material for: Rapid transcriptional plasticity of duplicated gene clusters enables a clonally reproducing aphid to colonise diverse plant species
Source: Genome Biol. 2017 Feb 13;18:27. doi: 10.1186/s13059-016-1145-3 (PMC5304397; doi:10.1186/s13059-016-1145-3)
Supplement: Additional file 11: — Supplementary text: M. persicae phylome report. (DOCX 349 kb) [file 13059_2016_1145_MOESM11_ESM.docx]

**Additional File 11: *M. persicae* phylome report**

***Myzus persicae* phylome reconstruction and analysis**

Damian Loska^1,2^, Irene Julca^1,2^ , Toni Gabaldón^1,2,3^

1) Bioinformatics and Genomics Programme. Centre for Genomic Regulation (CRG). Barcelona Institute of Science and Technology. Dr. Aiguader, 88. 08003 Barcelona, Spain

2) Universitat Pompeu Fabra (UPF). 08003 Barcelona, Spain.

3) Institució Catalana de Recerca i Estudis Avançats (ICREA), Pg. Lluís Companys 23, 08010 Barcelona, Spain.

To gain insight into the evolution of *Myzus persicae* (G006) genome in the context of twenty other sequenced metazoan species (**Table 1**), we generated the complete collection of evolutionary histories of *M. persicae* genes (i.e. the phylome, see Materials and Methods). These gene trees were scanned to infer duplications and speciation events and derive orthology and paralogy relationships among homologous genes [1]. All resulting gene trees, alignments and orthology and paralogy predictions are available for download or browse at PhylomeDB [2]. To reconstruct the evolutionary relationships among the considered species, we concatenated the protein alignments of 234 genes, which had single-copy orthologs in at least 20 out of the 21 species considered (see Materials and Methods). The resulting highly supported topology (**Figure 1**) was congruent with current views on metazoan phylogeny [3] with the exception of the placement of *Pediculus humanus* which has been recovered as a sister to holometabolous insects in a recent phylogenomic study [4]. We used a phylostratigraphic approach [5] to provide a relative dating of detected duplications and computed the duplication densities per branch in the lineages leading to *M. persicae* (**Table 2**). We filtered out putative transposase families by considering families presenting large expansions and containing domains associated with known viruses or transposable elements activities (see Materials and Methods). The two largest duplication densities were found in the lineages preceding the diversification of the two sequenced aphids and the diversification of insects, respectively. We subsequently performed functional enrichment of families duplicated at each evolutionary period and found that transporters, endopeptidases, and hydrolytic enzymes, among other activities, were enriched in families duplicated before the divergence of *Myzus* and *Acyrthosiphon* (**Table 3**), consistent with a sap-feeding lifestyle. No functional enrichment was found in families expanded specifically in *M. persicae*.

Then we focused on *M. persicae*-specific duplications, including large expansions. A total of 3,173 (19,35%) *M. persicae* proteins have an in-paralog (a recent paralog resulting from a duplication that specifically occurred in the *M. persicae* lineage since its split from *Acyrthosiphon*). These paralogs could be assigned to 1,243 *M. persicae-*specific gene expansions events, of which most (1202, 96,70%) have a moderate size (2-5 in-paralogs, **Figure 2**). A few (4, 0,32%) large expansions (>13 in-paralogs) comprise (60 genes, 0,37%). Interestingly 2,013 *M. persicae*-specific in-paralogs (63%), were only present in G006 strain, as shown by a phylome reconstruction including the two strains (phylome 198 at phylomeDB.org). This result may point to differences in gene annotation between the two strains or to differential gene duplication and gene retention since the two strains separated.

### **Methods**

### ***M. persicae* phylome reconstruction**

The M. persicae phylome, comprising all evolutionary histories of *M. persicae* protein-coding genes was reconstructed using the PhylomeDB pipeline [6]. In brief, for each protein-coding gene in the *Myzus* genome we searched for homologs (Smith-Waterman Blast search, using an e-value cutoff <1e-05, minimum contiguous overlap over the query sequence cutoff 50%) in a protein database containing the proteomes of the 21 species considered (**Table 1**). The most similar 150 homologues were aligned using three different programs (MUSCLE [7], MAFFT [8] and KALIGN [9] in forward and reverse direction. These six alignments were combined using M-COFFEE [10], and trimmed with trimAl v.1.3 [11] using a consistency cut-off of 0.16667 and a gap threshold of 0.1). Phylogenetic trees were built using Maximum Likelihood approach as implemented in PhyML v3.0 [12] using the best fitting model among seven different ones (JTT, LG, WAG, Blosum62, MtREV, VT and Dayhoff) was computed. The two models best fitting the data were determined based on likelihoods of an initial Neighbor Joining tree topology and using the AIC criterion. We used four rate categories and inferred fraction of invariant positions and rate parameters from the data. All alignments and trees are available for browsing or download at PhylomeDB with the PhylomeID 353 [2].

**Table 1**: List of species used in the phylome reconstruction. First column indicates taxa id, second column contains the species name and the third shows the source for the protein and the coding DNA sequences.

| **TaxID** | **Species name** | **Source of protein coding sequences** |
| --- | --- | --- |
| 13164 | *Myzus persicae G006* | *Myzus persicae* genome sequencing project |
| 121224 | *Pediculus humanus corporis* | Ensembl Metazoa release 25 |
| 12957 | *Atta cephalotes* | Ensembl Metazoa release 25 |
| 13037 | *Danaus plexippus* | Ensembl Metazoa release 25 |
| 13249 | *Rhodnius prolixus* | Ensembl Genomes - 23 |
| 13686 | *Solenopsis invicta* | Ensembl Metazoa release 25 |
| 248314 | *Heliconius melpomene plesseni* | Ensembl Metazoa release 25 |
| 32264 | *Tetranychus urticae* | Ensembl Metazoa release 25 |
| 36166 | *Megaselia scalaris* | Ensembl Metazoa release 25 |
| 43151 | *Anopheles darlingi* | Ensembl Metazoa release 25 |
| 6669 | *Daphnia pulex* | Ensembl Metazoa release 25 |
| 7029 | *Acyrthosiphon pisum* | Ensembl Metazoa release 25 |
| 7070 | *Tribolium castaneum* | Ensembl Metazoa release 25 |
| 7091 | *Bombyx mori* | Ensembl Metazoa release 25 |
| 7159 | *Aedes aegypti* | Ensembl Metazoa release 25 |
| 7165 | *Anopheles gambiae* | Ensembl Metazoa release 25 |
| 7176 | *Culex quinquefasciatus* | Ensembl Metazoa release 25 |
| 7227 | *Drosophila melanogaster* | Ensembl Metazoa release 25 |
| 7425 | *Nasonia vitripennis* | Ensembl Metazoa release 25 |
| 7460 | *Apis mellifera* | Baylor College of Medicine |
| 77166 | *Dendroctonus ponderosae* | Ensembl Genomes - 23 |

### **Prediction of gene duplications, and orthology and paralogy relationships**

Orthology and paralogy relationships were predicted based on phylogenetic evidence from the *Myzus* phylome. We used ETE v2 [13] to infer duplication and speciation relationships using a species overlap approach and a species overlap score of 0. The relative age of detected duplications was estimated using a phylostratigraphic approach that uses the information on which species diverged prior and after the duplication node [5]. We performed Gene Ontology (GO) terms enrichment analysis with FatiGO [14] by comparing annotations of the proteins involved in a duplication at a given age against the remaining myzus genes. All orthology and paralogy relationships are available through PhylomeDB [2]. HMMER v3.1b2 [15] was used to find domains that contain homology with viral and transposable elements (based on Pfam-A.hmm domains collection). Additional filtration was performed based on GO functional annotation. 1988 proteins have been found that have a homology to TE.

###

### **Species tree reconstruction**

We selected 234 genes that had one-to-one orthologs in at least 20 of the 21 species considered and concatenated their trimmed alignments, as reconstructed in the phylome. The final alignment containing 138,334 amino acid positions was used to reconstruct the maximum likelihood species tree with PhyML v3.0 [12, 16] using LG as a model, and estimating four rate categories and fraction of invariant sites from the alignments. Supports for the topology were computed using approximate Likelihood Ratio Tests and 100 bootstrap alignment replicates.


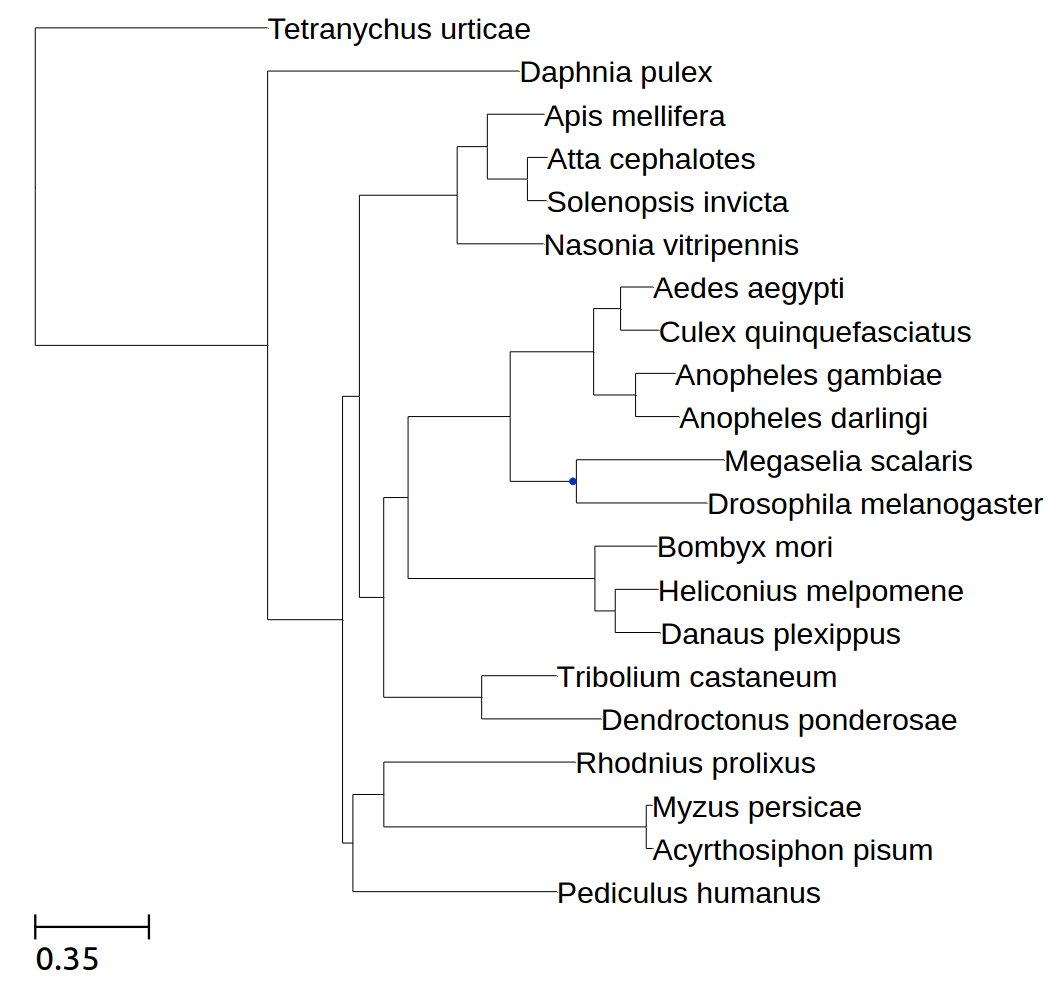


**Figure 1**: Species tree obtained from the concatenation of 234 widespread single-gene families. Species names in bold indicate genomes that have been sequenced in this study. All branches received maximal support (aLRT=1.0, bootstrap 100%).


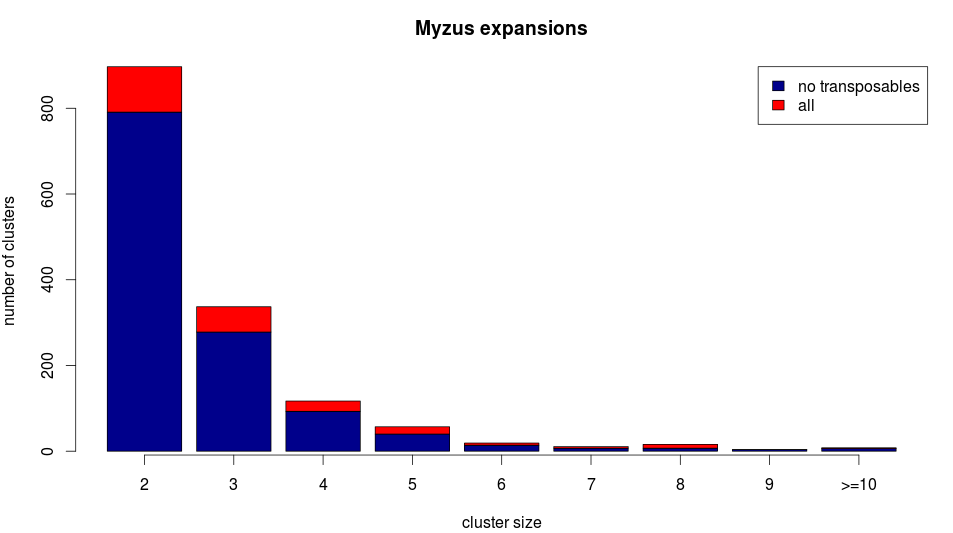


**Figure 2.** Distribution of size of in-paralog groups resulting from *M. persicae*-specific duplications. Red fraction of the bar indicates gene family expansions that were eliminated because the presence of TE-associated domains was detected.

**Table 2**: Computed duplication densities (average inferred duplications per gene lineage at that branch), after removing expansions (events involving more than five duplications in the same gene at the same branch).

| ***M. persicae* specific** | 0.15 | None |
| --- | --- | --- |
| **Aphids** | 1.78 | Transmembrane transport, Proteolysis,  Cellular alcohol metabolic process |
| **Hemiptera** | 0.04 | Transmembrane transport |
| **Paraneoptera** | 1.28 | Transmembrane transport, lipid metabolism, oxidative stress response, trehalose metabolism, cellular alcohol metabolic process |
| **Hexapoda** | 0.55 | Response to oxidative stress, Microtubule-based movement, proteolysis |
| **Mandibulata** | 2.09 | Transmembrane transport, signal transduction, response to oxidative stress |

**References**

1. Gabaldón T. Large-scale assignment of orthology: back to phylogenetics? Genome Biol 2008, 9:235.

2. Huerta-Cepas J, Capella-Gutiérrez S, Pryszcz LP, Marcet-Houben M, Gabaldón T. PhylomeDB v4: zooming into the plurality of evolutionary histories of a genome. Nucleic Acids Res 2014, 42:D897-902.

3. Srivastava M, Simakov O, Chapman J, Fahey B, Gauthier ME, Mitros T, Richards GS, Conaco C, Dacre M, Hellsten U, Larroux C, Putnam NH, Stanke M, Adamska M, Darling A, Degnan SM, Oakley TH, Plachetzki DC, Zhai Y, Adamski M, Calcino A, Cummins SF, Goodstein DM, Harris C, Jackson DJ, Leys SP, Shu S, Woodcroft BJ, Vervoort M, Kosik KS, Manning G, Degnan BM, Rokhsar DS. The Amphimedon queenslandica genome and the evolution of animal complexity. Nature 2010, 466:720-726.

4. Misof B, Liu S, Meusemann K, Peters RS, Donath A, Mayer C, Frandsen PB, Ware J, Flouri T, Beutel RG, Niehuis O, Petersen M, Izquierdo-Carrasco F, Wappler T, Rust J, Aberer AJ, Aspöck U, Aspöck H, Bartel D, Blanke A, Berger S, Böhm A, Buckley TR, Calcott B, Chen J, Friedrich F, Fukui M, Fujita M, Greve C, Grobe P, Gu S, Huang Y, Jermiin LS, Kawahara AY, Krogmann L, Kubiak M, Lanfear R, Letsch H, Li Y, Li Z, Li J, Lu H, Machida R, Mashimo Y, Kapli P, McKenna DD, Meng G, Nakagaki Y, Navarrete-Heredia JL, Ott M, Ou Y, Pass G, Podsiadlowski L, Pohl H, von Reumont BM, Schütte K, Sekiya K, Shimizu S, Slipinski A, Stamatakis A, Song W, Su X, Szucsich NU, Tan M, Tan X, Tang M, Tang J, Timelthaler G, Tomizuka S, Trautwein M, Tong X, Uchifune T, Walzl MG, Wiegmann BM, Wilbrandt J, Wipfler B, Wong TK, Wu Q, Wu G, Xie Y, Yang S, Yang Q, Yeates DK, Yoshizawa K, Zhang Q, Zhang R, Zhang W, Zhang Y, Zhao J, Zhou C, Zhou L, Ziesmann T, Zou S, Li Y, Xu X, Zhang Y, Yang H, Wang J, Wang J, Kjer KM, Zhou X. Phylogenomics resolves the timing and pattern of insect evolution. Science 2014, 346**:**763-767.

5. Huerta-Cepas J, Gabaldón T. Assigning duplication events to relative temporal scales in genome-wide studies. Bioinformatics 2011, 27:38-45.

6. Huerta-Cepas J, Capella-Gutierrez S, Pryszcz LP, Denisov I, Kormes D, Marcet-Houben M, Gabaldón T. PhylomeDB v3.0: an expanding repository of genome-wide collections of trees, alignments and phylogeny-based orthology and paralogy predictions. Nucleic Acids Res 2011, 39:D556-560.

7. Edgar RC. MUSCLE: multiple sequence alignment with high accuracy and high throughput. Nucleic Acids Res 2004, 32:1792-1797.

8. Katoh K, Kuma K, Toh H, Miyata T. MAFFT version 5: improvement in accuracy of multiple sequence alignment. Nucleic Acids Res 2005, 33:511-518.

9. Lassmann T, Sonnhammer EL. Kalign-an accurate and fast multiple sequence alignment algorithm. BMC Bioinformatics 2005, 6:298.

10. Wallace IM, O'Sullivan O, Higgins DG, Notredame C. M-Coffee: combining multiple sequence alignment methods with T-Coffee. Nucleic Acids Res 2006, 34:1692-1699.

11. Capella-Gutiérrez S, Silla-Martínez JM, Gabaldón T.trimAl: a tool for automated alignment trimming in large-scale phylogenetic analyses. Bioinformatics 2009, 25:1972-1973.

12. Guindon S, Dufayard JF, Lefort V, Anisimova M, Hordijk W, Gascuel O. New algorithms and methods to estimate maximum-likelihood phylogenies: assessing the performance of PhyML 3.0. Syst Biol 2010, 59:307-321.

13. Huerta-Cepas J, Marcet-Houben M, Pignatelli M, Moya A, Gabaldón T. The pea aphid phylome: a complete catalogue of evolutionary histories and arthropod orthology and paralogy relationships for Acyrthosiphon pisum genes. Insect Mol Biol 2010, 19 Suppl 2:13-21.

14. Al-Shahrour F, Minguez P, Tárraga J, Medina I, Alloza E, Montaner D, Dopazo J. FatiGO +: a functional profiling tool for genomic data. Integration of functional annotation, regulatory motifs and interaction data with microarray experiments. Nucleic Acids Res 2007, 35:W91-96.

15. Finn RD, Clements J, Eddy SR. HMMER web server: interactive sequence similarity searching. Nucleic Acids Res 2011, 39:W29-37.

16. Guindon S, Gascuel O: A simple, fast, and accurate algorithm to estimate large phylogenies by maximum likelihood. Syst Biol 2003, 52:696-704.
