# Supplementary material for: Rapid transcriptional plasticity of duplicated gene clusters enables a clonally reproducing aphid to colonise diverse plant species
Source: Genome Biol. 2017 Feb 13;18:27. doi: 10.1186/s13059-016-1145-3 (PMC5304397; doi:10.1186/s13059-016-1145-3)
Supplement: Additional file 12: Table S5. — M. persicae genes that are differentially expressed in aphids reared on different host plants. Genes that show differential expression (>1.5-fold with 10% FDR) on Nicotiana benthamiana vs. Brassica rapa (Nb/Br) are listed and grouped by KEGG functional classification. Top: genes more highly expressed on B. rapa; bottom: more highly expressed on N. benthamiana. Fold-change is the average over three biological replicates on each host plant, p value and FDR-adjusted p value (padj) based on DE-seq analysis. Annotations were conducted by NCBI blastX using cDNA sequences. FC is fold-change of Nb expression vs. Br. The presence of a predicted secretory signal peptide is indicated with ‘*’ in the SP column. Tissue-specific expression is indicated with ‘+’ in the ‘SG’ (salivary gland), ‘Gut’ and ‘Head’ columns, based on detection of the sequence in tissue-specific EST data [23]. (DOCX 157 kb) [file 13059_2016_1145_MOESM12_ESM.docx]

**Table S5:** *M. persicae* genes that are differentially expressed in aphids reared on different host plants. Genes that show differential expression (>1.5-fold with 10% FDR) on *Nicotiana benthamiana* vs. *Brassica rapa* (Nb/Br) are listed and grouped by KEGG functional classification. Top: genes more highly expressed on *B. rapa,* and bottom: more highly expressed on *N. benthamiana.* Fold-change is the average over 3 biological replicates on each host plant, *p-*value and FDR-adjusted-*p-*value (padj) based on DE-seq analysis. Annotations were conducted by NCBI blastX using cDNA sequences. FC is fold-change of Nb expression vs. Br. The presence of a predicted secretory signal peptide is indicated with “*” in the SP column. Tissue-specific expression is indicated with “+” in the “SG” (salivary gland), “Gut” and “Head” columns, based on detection of the sequence in tissue-specific EST data [23].

* Gene from MCL family significantly expanded in both aphid species vs. other insects (binomial test, Additional File 9: Table S4A)

** Gene from MCL family significantly expanded in *M. persicae* but not expanded in *A. pisum* (binomial test, Additional File 9: Table S4A)

^ Gene from aphid specific MCL family (found in *M. persicae* and *A. pisum*)

^^ Gene from *M. persicae* specific MCL family

Genes showing elevated expression in aphids reared on *B. rapa* vs. *N. benthamiana*

| **ID** | **Scaffold** | | **FC** | **logFC** | **p-val** | | **padj** | **MCL family** | **Annotation** | **SP** | **SG** | **Gut** | **Head** |
| --- | --- | --- | --- | --- | --- | --- | --- | --- | --- | --- | --- | --- | --- |
| **Metabolism (38)** |  | |  |  |  | |  |  |  |  |  |  |  |
| MYZPE13164_G006_v1.0_000091180 | 261 | | 0.58 | -0.79 | 1.94E-15 | | 2.69E-12 | family_95 | 1,5-anhydro-D-fructose reductase |  |  | + |  |
| MYZPE13164_G006_v1.0_000086200^ | 246 | | 0.65 | -0.63 | 1.63E-04 | | 1.05E-02 | family_16545 | Acid trehalase |  | + |  | + |
| MYZPE13164_G006_v1.0_000101050 | 295 | | 0.43 | -1.23 | 7.30E-08 | | 1.31E-05 | family_104 | Acyl-CoA Delta(11) desaturase-like |  |  |  |  |
| MYZPE13164_G006_v1.0_000015120 | 113 | | 0.23 | -2.13 | 4.09E-33 | | 4.54E-29 | family_179 | Alkaline phosphatase |  |  |  |  |
| MYZPE13164_G006_v1.0_000015140 | 113 | | 0.42 | -1.24 | 4.97E-17 | | 9.20E-14 | family_179 | Alkaline phosphatase | * |  | + |  |
| MYZPE13164_G006_v1.0_000202930 | 98 | | 0.55 | -0.87 | 2.13E-08 | | 4.30E-06 | family_208 | Alkaline-phosphatase-like |  |  |  |  |
| MYZPE13164_G006_v1.0_000029680 | 13 | | 0.36 | -1.46 | 1.81E-03 | | 7.47E-02 | family_1791 | Alpha-1,3-mannosyl-glycoprotein 4-beta-N-acetylglucosaminyltransferase B-like |  |  |  |  |
| MYZPE13164_G006_v1.0_000198050 | 93 | | 0.63 | -0.67 | 4.36E-04 | | 2.43E-02 | family_312 | Arylalkylamine N-acetyltransferase 4 isoform A |  |  |  |  |
| MYZPE13164_G006_v1.0_000111320 | 338 | | 0.43 | -1.21 | 1.76E-06 | | 2.42E-04 | family_6 | Cytochrome P450, E-class, group I |  |  |  |  |
| MYZPE13164_G006_v1.0_000111280 | 338 | | 0.48 | -1.06 | 2.47E-11 | | 1.19E-08 | family_6 | Cytochrome P450, E-class, group I |  |  |  |  |
| MYZPE13164_G006_v1.0_000111270 | 338 | | 0.53 | -0.91 | 1.90E-07 | | 3.24E-05 | family_1688 | Cytochrome P450, E-class, group I |  |  | + |  |
| MYZPE13164_G006_v1.0_000111310 | 338 | | 0.6 | -0.74 | 1.37E-09 | | 4.02E-07 | family_6 | Cytochrome P450, E-class, group I |  |  | + | + |
| MYZPE13164_G006_v1.0_000149510** | 548 | | 0.38 | -1.41 | 2.07E-08 | | 4.30E-06 | family_70 | Dehydrogenase/reductase SDR family member 11-like isoform X1 |  |  |  |  |
| MYZPE13164_G006_v1.0_000165290 | 659 | | 0.5 | -0.99 | 5.95E-08 | | 1.10E-05 | family_48 | Elongation of very long chain fatty acids protein 4-like |  |  |  | + |
| MYZPE13164_G006_v1.0_000137950 | 47 | | 0.42 | -1.26 | 8.21E-06 | | 8.83E-04 | family_234 | Glutathione S-transferase |  | + | + |  |
| MYZPE13164_G006_v1.0_000137960 | 47 | | 0.6 | -0.74 | 1.99E-08 | | 4.24E-06 | family_234 | Glutathione S-transferase |  | + | + | + |
| MYZPE13164_G006_v1.0_000015000 | 113 | | 0.61 | -0.71 | 1.24E-08 | | 2.82E-06 | family_234 | Glutathione S-transferase |  | + | + | + |
| MYZPE13164_G006_v1.0_000159190* | 615 | | 0.37 | -1.44 | 1.34E-08 | | 2.97E-06 | family_336 | Juvenile hormone-inducible protein |  |  | + |  |
| MYZPE13164_G006_v1.0_000159120* | 615 | | 0.4 | -1.32 | 8.08E-06 | | 8.80E-04 | family_336 | Juvenile hormone-inducible protein |  |  |  |  |
| MYZPE13164_G006_v1.0_000159090* | 615 | | 0.5 | -0.99 | 2.45E-07 | | 3.95E-05 | family_336 | Juvenile hormone-inducible protein |  |  |  |  |
| MYZPE13164_G006_v1.0_000114500 | 351 | | 0.38 | -1.39 | 1.22E-06 | | 1.70E-04 | family_16 | Lipoprotein lipase-like |  |  |  |  |
| MYZPE13164_G006_v1.0_000012210* | 11 | | 0.24 | -2.08 | 3.65E-10 | | 1.35E-07 | family_140 | Maltase 2-like | * |  | + |  |
| MYZPE13164_G006_v1.0_000148570* | 54 | | 0.44 | -1.18 | 1.94E-05 | | 1.84E-03 | family_140 | Maltase 2-like | * |  |  |  |
| MYZPE13164_G006_v1.0_000118900 | 37 | | 0.32 | -1.63 | 3.20E-20 | | 1.19E-16 | family_16 | Pancreatic lipase-related protein 2-like | * | + | + |  |
| MYZPE13164_G006_v1.0_000114510 | 351 | | 0.2 | -2.31 | 1.09E-06 | | 1.53E-04 | family_16 | Pancreatic lipase-related protein 2-like | * | + | + | + |
| MYZPE13164_G006_v1.0_000114520 | 351 | | 0.64 | -0.64 | 1.03E-07 | | 1.81E-05 | family_16 | Pancreatic lipase-related protein 2-like | * |  | + |  |
| MYZPE13164_G006_v1.0_000090160 | 258 | | 0.38 | -1.41 | 7.23E-09 | | 1.78E-06 | family_207 | Probable phospholipid-transporting ATPase IM |  | + |  |  |
| MYZPE13164_G006_v1.0_000146250 | 522 | | 0.54 | -0.9 | 9.18E-05 | | 6.46E-03 | family_207 | Probable phospholipid-transporting ATPase IM |  |  |  |  |
| MYZPE13164_G006_v1.0_000152530^^ | 57 | | 0.57 | -0.81 | 8.91E-12 | | 5.21E-09 | family_65328 | Prostatic spermine-binding protein-like |  | + |  |  |
| MYZPE13164_G006_v1.0_000075890** | 213 | | 0.48 | -1.04 | 2.85E-04 | | 1.70E-02 | family_70 | Short-chain dehydrogenase/reductase |  |  |  |  |
| MYZPE13164_G006_v1.0_000171550 | 706 | | 0.53 | -0.93 | 2.12E-08 | | 4.30E-06 | family_590 | Short-chain dehydrogenase/reductase |  |  | + |  |
| MYZPE13164_G006_v1.0_000194850* | 9 | | 0.42 | -1.26 | 5.08E-05 | | 4.09E-03 | family_12 | UDP-glucosyltransferase | * |  | + |  |
| MYZPE13164_G006_v1.0_000065900* | 19 | | 0.59 | -0.76 | 6.39E-05 | | 5.03E-03 | family_12 | UDP-glucosyltransferase | * | + |  |  |
| MYZPE13164_G006_v1.0_000148750* | 540 | | 0.59 | -0.76 | 7.97E-11 | | 3.28E-08 | family_12 | UDP-glucosyltransferase | * |  |  |  |
| MYZPE13164_G006_v1.0_000150840* | 555 | | 0.28 | -1.81 | 1.63E-08 | | 3.55E-06 | family_12 | UDP-glucosyltransferase | * | + | + |  |
| MYZPE13164_G006_v1.0_000150850* | 555 | | 0.32 | -1.65 | 6.98E-07 | | 1.03E-04 | family_12 | UDP-glucosyltransferase |  |  | + |  |
| MYZPE13164_G006_v1.0_000150810* | 555 | | 0.48 | -1.06 | 1.67E-03 | | 7.07E-02 | family_12 | UDP-glucosyltransferase | * |  |  |  |
| MYZPE13164_G006_v1.0_000150820* | 615 | | 0.38 | -1.41 | 9.30E-09 | | 2.20E-06 | family_12 | UDP-glucosyltransferase | * | + | + |  |
| **Transcription-related (7)** |  | |  |  |  | |  |  |  |  |  |  |  |
| MYZPE13164_G006_v1.0_000037740 | 140 | | 0.67 | -0.58 | 8.62E-06 | | 9.03E-04 | family_8389 | Homeobox protein vnd-like |  |  |  |  |
| MYZPE13164_G006_v1.0_000171540 | 706 | | 0.43 | -1.21 | 2.78E-04 | | 1.67E-02 | family_349 | Mediator of RNA polymerase II transcription subunit 12 |  |  |  |  |
| MYZPE13164_G006_v1.0_000030960 | 131 | | 0.51 | -0.97 | 8.10E-10 | | 2.65E-07 | family_343 | Natterin-3 |  |  | + |  |
| MYZPE13164_G006_v1.0_000180720^ | 78 | | 0.42 | -1.26 | 1.33E-16 | | 2.11E-13 | family_6028 | Stress response protein NST1-like |  | + |  |  |
| MYZPE13164_G006_v1.0_000072770 | 205 | | 0.65 | -0.62 | 1.58E-03 | | 6.77E-02 | family_4711 | U6 snRNA-associated Sm-like protein |  |  |  |  |
| MYZPE13164_G006_v1.0_000062520* | 183 | | 0.5 | -1.01 | 4.11E-04 | | 2.32E-02 | family_3 | Zinc finger MYM-type protein 1-like |  |  |  |  |
| MYZPE13164_G006_v1.0_000006110^ | 1037 | | 0.6 | -0.74 | 7.24E-08 | | 1.31E-05 | family_17122 | Zinc finger protein 853-like | * | + | + | + |
| **Peptidase (16)** |  | |  |  |  | |  |  |  |  |  |  |  |
| MYZPE13164_G006_v1.0_000125260 | 40 | | 0.27 | -1.9 | 1.81E-05 | | 1.75E-03 | family_37 | Aminopeptidase N precursor | * |  |  |  |
| MYZPE13164_G006_v1.0_000125270 | 40 | | 0.36 | -1.48 | 3.73E-09 | | 1.03E-06 | family_37 | Aminopeptidase N-like | * |  |  |  |
| MYZPE13164_G006_v1.0_000144060 | 508 | | 0.62 | -0.68 | 1.18E-11 | | 6.55E-09 | family_37 | Aminopeptidase N-like | * |  | + |  |
| MYZPE13164_G006_v1.0_000195260* | 90 | | 0.58 | -0.8 | 2.82E-05 | | 2.49E-03 | family_110 | Cathepsin B | * |  |  |  |
| MYZPE13164_G006_v1.0_000049160* | 158 | | 0.16 | -2.6 | 4.37E-05 | | 3.60E-03 | family_110 | Cathepsin B | * |  |  |  |
| MYZPE13164_G006_v1.0_000090040* | 258 | | 0.35 | -1.52 | 5.45E-10 | | 1.92E-07 | family_110 | Cathepsin B |  |  |  |  |
| MYZPE13164_G006_v1.0_000104270* | 304 | | 0.25 | -2.02 | 1.83E-05 | | 1.75E-03 | family_110 | Cathepsin B | * |  |  |  |
| MYZPE13164_G006_v1.0_000104300* | 304 | | 0.27 | -1.9 | 1.34E-09 | | 4.01E-07 | family_110 | Cathepsin B | * | + | + |  |
| MYZPE13164_G006_v1.0_000104310* | 304 | | 0.31 | -1.7 | 1.52E-10 | | 5.83E-08 | family_110 | Cathepsin B | * |  | + |  |
| MYZPE13164_G006_v1.0_000141000* | 491 | | 0.16 | -2.63 | 7.83E-05 | | 5.83E-03 | family_110 | Cathepsin B | * |  |  |  |
| MYZPE13164_G006_v1.0_000141010* | 491 | | 0.2 | -2.34 | 6.13E-05 | | 4.87E-03 | family_110 | Cathepsin B | * | + |  |  |
| MYZPE13164_G006_v1.0_000150170* | 550 | | 0.51 | -0.96 | 2.71E-05 | | 2.43E-03 | family_110 | cathepsin B | * | + | + |  |
| MYZPE13164_G006_v1.0_000151060* | 558 | | 0.36 | -1.47 | 5.29E-04 | | 2.89E-02 | family_110 | cathepsin B |  |  |  |  |
| MYZPE13164_G006_v1.0_000088150 | 251 | | 0.54 | -0.88 | 2.98E-05 | | 2.57E-03 | family_855 | Legumain-like |  |  | + |  |
| MYZPE13164_G006_v1.0_000088160 | 251 | | 0.66 | -0.6 | 3.14E-04 | | 1.84E-02 | family_855 | Legumain-like | * |  |  |  |
| MYZPE13164_G006_v1.0_000117340^ | 365 | | 0.41 | -1.29 | 2.47E-04 | | 1.49E-02 | family_7661 | Peptidase | * |  | + |  |
| **Transport (15)** |  | |  |  |  | |  |  |  |  |  |  |  |
| MYZPE13164_G006_v1.0_000061310 | 181 | | 0.61 | -0.72 | 7.06E-09 | | 1.78E-06 | family_166 | Amino acid/polyamine transporter I |  |  | + |  |
| MYZPE13164_G006_v1.0_000186250 | 82 | | 0.6 | -0.73 | 3.50E-06 | | 4.57E-04 | family_162 | ATP-binding cassette sub-family A member 3 |  |  |  |  |
| MYZPE13164_G006_v1.0_000116830* | 361 | | 0.64 | -0.63 | 6.84E-05 | | 5.22E-03 | family_7 | Facilitated trehalose transporter Tret1-like |  |  |  |  |
| MYZPE13164_G006_v1.0_000072950* | 2058 | | 0.6 | -0.73 | 2.07E-07 | | 3.48E-05 | family_7 | Facilitated trehalose transporter Tret1-like |  |  |  |  |
| MYZPE13164_G006_v1.0_000012150 | 11 | | 0.4 | -1.34 | 2.31E-15 | | 2.85E-12 | family_6232 | G-protein coupled receptor | * |  |  |  |
| MYZPE13164_G006_v1.0_000181350 | 787 | | 0.62 | -0.69 | 2.38E-03 | | 9.06E-02 | family_966 | Major facilitator superfamily domain-containing protein 12-like isoform X1 |  |  |  |  |
| MYZPE13164_G006_v1.0_000197280* | 92 | | 0.4 | -1.32 | 2.45E-07 | | 3.95E-05 | family_253 | Major facilitator superfamily domain-containing protein 6-like |  |  |  |  |
| MYZPE13164_G006_v1.0_000166060* | 666 | | 0.26 | -1.94 | 3.29E-08 | | 6.30E-06 | family_253 | Major facilitator superfamily domain-containing protein 6-like |  |  |  |  |
| MYZPE13164_G006_v1.0_000201330^ | 966 | | 0.56 | -0.84 | 1.52E-17 | | 3.38E-14 | family_14070 | MD-2-related lipid-recognition protein-like |  |  |  |  |
| MYZPE13164_G006_v1.0_000010570 | 1080 | | 0.62 | -0.69 | 7.20E-07 | | 1.05E-04 | family_32 | Multidrug resistance-associated protein 1-like isoform X1 | * |  |  |  |
| MYZPE13164_G006_v1.0_000086620 | 248 | | 0.52 | -0.95 | 3.77E-11 | | 1.75E-08 | family_32 | Multidrug resistance-associated protein lethal(2)03659 |  |  | + |  |
| MYZPE13164_G006_v1.0_000086580 | 248 | | 0.6 | -0.74 | 6.91E-10 | | 2.33E-07 | family_32 | Multidrug resistance-associated protein lethal(2)03659 isoform X1 |  | + | + | + |
| MYZPE13164_G006_v1.0_000168390* | 687 | | 0.38 | -1.41 | 8.90E-09 | | 2.15E-06 | family_120 | Proton-coupled folate transporter-like |  |  | + | + |
| MYZPE13164_G006_v1.0_000168370* | 687 | | 0.29 | -1.76 | 3.02E-09 | | 8.59E-07 | family_120 | Proton-coupled folate transporter-like |  |  | + |  |
| MYZPE13164_G006_v1.0_000033980* | 135 | | 0.47 | -1.08 | 7.31E-07 | | 1.05E-04 | family_120 | Solute carrier family 46 member 3-like |  |  |  |  |
| MYZPE13164_G006_v1.0_000033970 | 135 | | 0.56 | -0.84 | 1.47E-03 | | 6.46E-02 | family_20295 | Solute carrier family 46 member 3-like |  |  |  |  |
| **Protein modification (5)** |  | |  |  |  | |  |  |  |  |  |  |  |
| MYZPE13164_G006_v1.0_000121890 | 383 | | 0.38 | -1.38 | 3.37E-04 | | 1.94E-02 | family_1315 | Endoplasmic reticulum resident protein 44-like isoform X3 |  | + |  |  |
| MYZPE13164_G006_v1.0_000176290^ | 742 | | 0.48 | -1.07 | 2.43E-05 | | 2.21E-03 | family_4458 | Myotubularin-related protein 10-B |  |  |  |  |
| MYZPE13164_G006_v1.0_000201080^^ | 926 | | 0.18 | -2.46 | 5.56E-12 | | 3.43E-09 | family_27968 | Small ubiquitin-related modifier-like |  |  |  |  |
| MYZPE13164_G006_v1.0_000069110 | 199 | | 0.62 | -0.68 | 9.57E-05 | | 6.69E-03 | family_1693 | Tetraspanin-6 |  |  | + |  |
| **Others (29)** |  | |  |  |  | |  |  |  |  |  |  |  |
| MYZPE13164_G006_v1.0_000121420 | 381 | | 0.04 | -4.61 | 2.35E-05 | | 2.16E-03 | family_6610 | A-agglutinin anchorage subunit-like isoform X1 | * | + | + |  |
| MYZPE13164_G006_v1.0_000162270 | 639 | | 0.49 | -1.02 | 2.19E-07 | | 3.64E-05 | family_26 | Alpha-tocopherol transfer protein-like |  |  | + | + |
| MYZPE13164_G006_v1.0_000027490 | 128 | | 0.62 | -0.7 | 2.80E-07 | | 4.45E-05 | family_168 | Arrestin-like |  |  |  |  |
| MYZPE13164_G006_v1.0_000183390 | 8 | | 0.62 | -0.69 | 4.60E-06 | | 5.61E-04 | family_5536 | inhibin beta chain-like | * |  |  |  |
| MYZPE13164_G006_v1.0_000144040^^ | 508 | | 0.34 | -1.58 | 8.01E-14 | | 6.36E-11 | family_65302 | integumentary mucin A.1-like |  |  |  | + |
| MYZPE13164_G006_v1.0_000163150 | 641 | | 0.64 | -0.64 | 1.98E-06 | | 2.68E-04 | family_7832 | La protein homolog |  |  |  |  |
| MYZPE13164_G006_v1.0_000132670* | 436 | | 0.24 | -2.06 | 1.70E-03 | | 7.15E-02 | family_92 | Nucleic-acid-binding protein from mobile element jockey-like |  |  |  |  |
| MYZPE13164_G006_v1.0_000124180** | 395 | | 0.54 | -0.89 | 1.48E-03 | | 6.46E-02 | family_1032 | PDGF- and VEGF-related factor 1-like precursor | * |  | + |  |
| MYZPE13164_G006_v1.0_000017390^ | 116 | | 0.61 | -0.71 | 2.82E-05 | | 2.49E-03 | family_15160 | Protocadherin Fat 4-like |  |  | + | + |
| MYZPE13164_G006_v1.0_000186930^ | 826 | | 0.47 | -1.08 | 4.62E-04 | | 2.55E-02 | family_16565 | Titin-like isoform X3 | * | + |  | + |
| MYZPE13164_G006_v1.0_000200770* | 96 | | 0.28 | -1.84 | 8.99E-05 | | 6.36E-03 | family_333 | Transposase, ISXO2-like |  |  |  |  |
| MYZPE13164_G006_v1.0_000012450^ | 11 | | 0.57 | -0.82 | 6.73E-04 | | 3.54E-02 | family_16486 | Uncharacterized protein |  |  |  |  |
| MYZPE13164_G006_v1.0_000116630^ | 36 | | 0.53 | -0.92 | 3.37E-13 | | 2.50E-10 | family_16164 | Uncharacterized protein | * | + |  | + |
| MYZPE13164_G006_v1.0_000164330* | 65 | | 0.32 | -1.64 | 7.28E-06 | | 8.17E-04 | family_52 | Uncharacterized protein |  |  |  |  |
| MYZPE13164_G006_v1.0_000014130^ | 112 | | 0.27 | -1.88 | 3.87E-08 | | 7.30E-06 | family_22316 | Uncharacterized protein | * |  | + |  |
| MYZPE13164_G006_v1.0_000045040 | 150 | | 0.66 | -0.59 | 6.40E-06 | | 7.40E-04 | family_5440 | Uncharacterized protein | * |  |  |  |
| MYZPE13164_G006_v1.0_000062140^ | 182 | | 0.66 | -0.6 | 1.05E-10 | | 4.17E-08 | family_16393 | Uncharacterized protein | * |  | + |  |
| MYZPE13164_G006_v1.0_000087830^ | 250 | | 0.4 | -1.3 | 2.96E-05 | | 2.57E-03 | family_17160 | Uncharacterized protein |  |  |  |  |
| MYZPE13164_G006_v1.0_000095050 | 276 | | 0.65 | -0.61 | 3.68E-06 | | 4.76E-04 | family_6265 | Uncharacterized protein |  |  |  |  |
| MYZPE13164_G006_v1.0_000100340^ | 293 | | 0.59 | -0.76 | 1.56E-11 | | 7.90E-09 | family_17365 | Uncharacterized protein |  |  |  |  |
| MYZPE13164_G006_v1.0_000117350^ | 365 | | 0.44 | -1.2 | 2.67E-03 | | 9.85E-02 | family_7661 | Uncharacterized protein | * |  |  |  |
| MYZPE13164_G006_v1.0_000135370^ | 454 | | 0.5 | -1.01 | 5.50E-13 | | 3.82E-10 | family_16524 | Uncharacterized protein |  |  |  |  |
| MYZPE13164_G006_v1.0_000137430* | 467 | | 0.32 | -1.64 | 7.28E-06 | | 8.17E-04 | family_52 | Uncharacterized protein |  |  |  |  |
| MYZPE13164_G006_v1.0_000143710^ | 505 | | 0.51 | -0.96 | 1.42E-03 | | 6.35E-02 | family_17195 | Uncharacterized protein |  |  |  |  |
| MYZPE13164_G006_v1.0_000149220* | 545 | | 0.32 | -1.64 | 7.28E-06 | | 8.17E-04 | family_52 | Uncharacterized protein |  |  |  |  |
| MYZPE13164_G006_v1.0_000151150^ | 559 | | 0.37 | -1.43 | 4.09E-06 | | 5.05E-04 | family_22203 | Uncharacterized protein |  |  | + |  |
| MYZPE13164_G006_v1.0_000168200^ | 683 | | 0.61 | -0.71 | 2.19E-05 | | 2.03E-03 | family_3600 | Uncharacterized protein |  |  |  |  |
| MYZPE13164_G006_v1.0_000198520^ | 936 | | 0.6 | -0.75 | 9.89E-10 | | 3.14E-07 | family_16406 | Uncharacterized protein | * | + |  | + |
| MYZPE13164_G006_v1.0_000048940 | 1572 | | 0.58 | -0.79 | 1.54E-03 | | 6.65E-02 | family_445 | Uncharacterized protein |  |  |  |  |
|  |  | |  |  |  | |  |  |  |  |  |  |  |
| Transcripts showing elevated expression in aphids reared on *N. benthamiana* vs. *B. rapa* | | | | | | | | | |  |  |  |  |
| **ID** | **Scaffold** | | **FC** | **logFC** | **p-val** | | **padj** | **MCL family** | **Annotation** | **SP** | **SG** | **Gut** | **Head** |
| **Metabolism (13)** |  |  | |  | |  |  |  |  |  |  |  |  |
| MYZPE13164_G006_v1.0_000183870 | 80 | 1.62 | | 0.69 | | 1.29E-03 | 5.87E-02 | family_202 | Aromatic-L-amino-acid decarboxylase |  |  |  |  |
| MYZPE13164_G006_v1.0_000078630 | 221 | 1.52 | | 0.6 | | 1.76E-04 | 1.11E-02 | family_5736 | Cyclase-like precursor | * |  |  |  |
| MYZPE13164_G006_v1.0_000145950 | 52 | 1.53 | | 0.61 | | 1.02E-04 | 6.98E-03 | family_6 | Cytochrome P450 6a14 | * |  |  |  |
| MYZPE13164_G006_v1.0_000159700 | 62 | 1.8 | | 0.85 | | 1.08E-08 | 2.51E-06 | family_28 | Fatty acyl-CoA reductase 1-like isoform X1 |  |  |  | + |
| MYZPE13164_G006_v1.0_000194120** | 9 | 1.59 | | 0.67 | | 3.10E-05 | 2.65E-03 | family_877 | Gamma-glutamyl hydrolase A-like | * |  |  |  |
| MYZPE13164_G006_v1.0_000044350^ | 15 | 1.81 | | 0.85 | | 5.15E-06 | 6.15E-04 | family_10732 | Macrophage migration inhibitory factor |  |  | + |  |
| MYZPE13164_G006_v1.0_000044340 | 15 | 1.57 | | 0.65 | | 7.00E-04 | 3.66E-02 | family_3021 | Macrophage migration inhibitory factor |  |  |  | + |
| MYZPE13164_G006_v1.0_000040710 | 144 | 1.7 | | 0.77 | | 3.83E-06 | 4.84E-04 | family_3021 | Macrophage migration inhibitory factor |  |  |  |  |
| MYZPE13164_G006_v1.0_000068520* | 197 | 1.75 | | 0.8 | | 4.46E-05 | 3.64E-03 | family_140 | Maltase 1-like | * |  |  |  |
| MYZPE13164_G006_v1.0_000104580* | 305 | 1.56 | | 0.64 | | 2.46E-03 | 9.31E-02 | family_41 | Peroxidase | * |  |  |  |
| MYZPE13164_G006_v1.0_000159210 | 615 | 1.72 | | 0.79 | | 3.72E-05 | 3.13E-03 | family_121 | Short-chain dehydrogenase/reductase family 16C member 6-like isoform X1 |  |  |  |  |
| MYZPE13164_G006_v1.0_000070810^ | 20 | 2.46 | | 1.3 | | 1.91E-03 | 7.77E-02 | family_11941 | UDP-glucuronosyltransferase 2B2-like |  |  |  |  |
| MYZPE13164_G006_v1.0_000099450* | 290 | 40.06 | | 5.32 | | 2.20E-08 | 4.30E-06 | family_12 | UDP-glucuronosyltransferase 2C1-like | * |  |  |  |
| **Cuticular protein (23)** |  |  | |  | |  |  |  |  |  |  |  |  |
| MYZPE13164_G006_v1.0_000122290* | 387 | 1.64 | | 0.72 | | 2.42E-14 | 2.44E-11 | family_27 | Cuticular protein, RR2 motif | * |  |  |  |
| MYZPE13164_G006_v1.0_000122370* | 387 | 1.9 | | 0.93 | | 2.46E-03 | 9.31E-02 | family_27 | Cuticular protein 19 precursor, RR2 motif | * |  |  |  |
| MYZPE13164_G006_v1.0_000122360^^ | 387 | 1.7 | | 0.76 | | 1.27E-03 | 5.79E-02 | family_24280 | Cuticular protein 20 precursor, RR2 motif | * |  |  |  |
| MYZPE13164_G006_v1.0_000122350 | 387 | 1.66 | | 0.73 | | 8.00E-05 | 5.89E-03 | family_6741 | Cuticular protein 21, RR2 motif | * |  |  | + |
| MYZPE13164_G006_v1.0_000122340* | 387 | 1.57 | | 0.65 | | 5.34E-11 | 2.28E-08 | family_27 | Cuticular protein 22 precursor, RR2 motif | * |  |  |  |
| MYZPE13164_G006_v1.0_000122320* | 387 | 1.63 | | 0.71 | | 3.37E-06 | 4.51E-04 | family_27 | Cuticular protein 25, RR2 motif | * | + |  |  |
| MYZPE13164_G006_v1.0_000122310* | 387 | 1.63 | | 0.7 | | 5.84E-09 | 1.51E-06 | family_27 | Cuticular protein 28 precursor, RR2 motif | * | + |  |  |
| MYZPE13164_G006_v1.0_000122300^^ | 387 | 1.51 | | 0.6 | | 1.22E-09 | 3.77E-07 | family_27324 | Cuticular protein 30 precursor, RR2 motif | * | + |  |  |
| MYZPE13164_G006_v1.0_000122270* | 387 | 1.66 | | 0.73 | | 1.33E-11 | 7.06E-09 | family_27 | Cuticular protein 35 precursor, RR2 motif | * |  |  |  |
| MYZPE13164_G006_v1.0_000122250* | 387 | 2.21 | | 1.14 | | 6.52E-05 | 5.10E-03 | family_27 | Cuticular protein 36, RR2 motif | * |  |  |  |
| MYZPE13164_G006_v1.0_000122240* | 387 | 1.67 | | 0.74 | | 3.99E-06 | 4.99E-04 | family_27 | Cuticular protein 36, RR2 motif | * |  |  |  |
| MYZPE13164_G006_v1.0_000167270* | 678 | 1.79 | | 0.84 | | 2.05E-05 | 1.93E-03 | family_27 | Cuticular protein 36, RR2 motif | * | + |  |  |
| MYZPE13164_G006_v1.0_000122330* | 387 | 1.86 | | 0.89 | | 4.29E-05 | 3.56E-03 | family_27 | Cuticular protein 38, RR2 motif | * |  |  |  |
| MYZPE13164_G006_v1.0_000167330* | 387 | 1.84 | | 0.88 | | 3.86E-09 | 1.03E-06 | family_27 | Cuticular protein 41 precursor, RR2 motif | * |  |  |  |
| MYZPE13164_G006_v1.0_000160430* | 624 | 1.67 | | 0.74 | | 2.78E-12 | 1.82E-09 | family_27 | Cuticular protein 43 precursor, RR2 motif | * |  |  | + |
| MYZPE13164_G006_v1.0_000122390* | 387 | 1.76 | | 0.82 | | 4.23E-04 | 2.38E-02 | family_27 | Cuticular protein 5 precursor, RR2 motif | * | + |  | + |
| MYZPE13164_G006_v1.0_000076360^^ | 214 | 1.75 | | 0.8 | | 1.07E-14 | 1.19E-11 | family_31267 | Cuticular protein 7 , RR2 motif |  | + | + | + |
| MYZPE13164_G006_v1.0_000122410* | 387 | 1.64 | | 0.71 | | 4.61E-14 | 4.27E-11 | family_27 | Cuticular protein 7 , RR2 motif |  |  |  |  |
| MYZPE13164_G006_v1.0_000167260* | 678 | 1.77 | | 0.83 | | 3.48E-04 | 1.99E-02 | family_27 | Cuticular protein 7-like , RR2 motif | * | + |  |  |
| MYZPE13164_G006_v1.0_000167280* | 678 | 1.71 | | 0.78 | | 1.54E-05 | 1.51E-03 | family_27 | Cuticular protein 7-like , RR2 motif | * |  |  |  |
| MYZPE13164_G006_v1.0_000122380* | 387 | 1.7 | | 0.77 | | 1.44E-05 | 1.44E-03 | family_27 | Cuticular protein 7-like precursor, RR2 motif | * | + |  |  |
| MYZPE13164_G006_v1.0_000008720 | 106 | 1.59 | | 0.67 | | 2.56E-05 | 2.31E-03 | family_169 | Cuticular protein analogous to peritrophins 3-D1 precursor, RR2 motif | * |  |  |  |
| MYZPE13164_G006_v1.0_000103390 | 300 | 1.88 | | 0.91 | | 4.62E-07 | 7.13E-05 | family_12083 | Cuticular protein RR-1 motif 47 precursor | * |  |  |  |
| **Transcription-related (8)** |  |  | |  | |  |  |  |  |  |  |  |  |
| MYZPE13164_G006_v1.0_000017400 | 116 | 1.53 | | 0.61 | | 8.53E-05 | 6.19E-03 | family_10026 | DNA-directed RNA polymerase II subunit RPB1-like | * | + |  | + |
| MYZPE13164_G006_v1.0_000111880^ | 34 | 1.8 | | 0.85 | | 1.37E-07 | 2.38E-05 | family_10956 | Enhancer of polycomb | * |  |  |  |
| MYZPE13164_G006_v1.0_000201620^ | 97 | 1.6 | | 0.68 | | 5.09E-06 | 6.15E-04 | family_16361 | Mediator of RNA polymerase II transcription subunit 12 | * | + |  |  |
| MYZPE13164_G006_v1.0_000144610^ | 511 | 1.55 | | 0.63 | | 2.42E-04 | 1.47E-02 | family_10967 | Mediator of RNA polymerase II transcription subunit 15-like/Chitin_bind_4_Insect cuticle protein |  |  |  |  |
| MYZPE13164_G006_v1.0_000115980* | 358 | 61.68 | | 5.95 | | 2.17E-08 | 4.30E-06 | family_102 | RNA-directed DNA polymerase from mobile element jockey |  |  |  |  |
| MYZPE13164_G006_v1.0_000094380* | 273 | ∞ | | ∞ | | 1.38E-18 | 3.85E-15 | family_102 | RNA-directed DNA polymerase from mobile element jockey-like |  |  |  |  |
| MYZPE13164_G006_v1.0_000000470^ | 0 | ∞ | | ∞ | | 4.15E-11 | 1.84E-08 | family_267 | SCAN domain-containing protein 3-like |  |  |  |  |
| MYZPE13164_G006_v1.0_000067270 | 194 | 1.9 | | 0.93 | | 1.70E-04 | 1.08E-02 | family_22834 | Zinc finger protein 852 |  |  |  |  |
| **Transport (5)** |  |  | |  | |  |  |  |  |  |  |  |  |
| MYZPE13164_G006_v1.0_000108020* | 322 | 4.23 | | 2.08 | | 1.61E-03 | 6.83E-02 | family_7 | Facilitated trehalose transporter Tret1-like |  |  |  |  |
| MYZPE13164_G006_v1.0_000061320* | 181 | 4.44 | | 2.15 | | 8.44E-29 | 4.69E-25 | family_166 | Low affinity cationic amino acid transporter 2-like |  |  |  |  |
| MYZPE13164_G006_v1.0_000170750 | 7 | 1.87 | | 0.9 | | 7.55E-14 | 6.36E-11 | family_109 | Sodium-independent sulfate anion transporter-like |  |  |  |  |
| MYZPE13164_G006_v1.0_000089360* | 256 | 2.4 | | 1.26 | | 5.52E-10 | 1.92E-07 | family_305 | Solute carrier family 15 member 2-like |  |  |  |  |
| MYZPE13164_G006_v1.0_000044810 | 15 | 1.94 | | 0.96 | | 2.35E-03 | 9.05E-02 | family_3770 | Vacuolar protein sorting- protein 62 | * |  |  |  |
| **Protein modification (3)** |  |  | |  | |  |  |  |  |  |  |  |  |
| MYZPE13164_G006_v1.0_000106370 | 313 | 1.53 | | 0.61 | | 1.94E-03 | 7.86E-02 | family_8238 | Death-associated protein kinase related-like |  |  |  |  |
| MYZPE13164_G006_v1.0_000074360* | 21 | 21.2 | | 4.41 | | 6.29E-07 | 9.44E-05 | family_39 | MULE and FLYWCH domain containing protein |  |  |  |  |
| MYZPE13164_G006_v1.0_000172320^ | 71 | 1.52 | | 0.6 | | 1.91E-04 | 1.20E-02 | family_22301 | Thioredoxin-like protein 1 |  |  |  |  |
| **Others (8)** |  |  | |  | |  |  |  |  |  |  |  |  |
| MYZPE13164_G006_v1.0_000074350 | 21 | 1.67 | | 0.74 | | 1.14E-04 | 7.69E-03 | family_30 | Juvenile hormone binding protein | * |  |  |  |
| MYZPE13164_G006_v1.0_000076370^^ | 214 | 1.55 | | 0.63 | | 1.38E-03 | 6.20E-02 | family_31268 | keratin, type I cytoskeletal 9-like | * |  |  |  |
| MYZPE13164_G006_v1.0_000143340* | 501 | 1.98 | | 0.99 | | 1.91E-03 | 7.77E-02 | family_79 | Nose resistant-to-fluoxetine protein | * |  |  |  |
| MYZPE13164_G006_v1.0_000157470* | 601 | 3.26 | | 1.7 | | 1.83E-05 | 1.75E-03 | family_333 | Transposase, ISXO2-like |  |  |  |  |
| MYZPE13164_G006_v1.0_000139270 | 480 | 112.78 | | 6.82 | | 1.80E-03 | 7.46E-02 | family_55 | Troponin C, isoform 1-like |  |  |  |  |
| MYZPE13164_G006_v1.0_000197380^^ | 92 | 1.63 | | 0.7 | | 5.55E-04 | 3.01E-02 | family_65628 | Uncharacterized protein |  | + |  |  |
| MYZPE13164_G006_v1.0_000008790 | 106 | 1.78 | | 0.83 | | 3.88E-09 | 1.03E-06 | family_1205 | Uncharacterized protein |  |  | + | + |
| MYZPE13164_G006_v1.0_000092160^ | 2657 | 1.54 | | 0.62 | | 2.70E-03 | 9.90E-02 | family_13150 | Uncharacterized protein |  |  |  |  |
