# Supplementary material for: Rapid transcriptional plasticity of duplicated gene clusters enables a clonally reproducing aphid to colonise diverse plant species
Source: Genome Biol. 2017 Feb 13;18:27. doi: 10.1186/s13059-016-1145-3 (PMC5304397; doi:10.1186/s13059-016-1145-3)

**Additional File 19: Cathepsin B dsRNA alignment**

| **Gene name** | **Gene ID** | **Score (bits)** | **E value** | **Reduced expression by *dsCathB* lines** | **Identities** | **Gaps** |
| --- | --- | --- | --- | --- | --- | --- |
| MpCathB4 | MYZPE13164_G006_v1.0_000104270 | 335 | 1.00E-91 | Yes | 241/242(99%)* | 0/242 (0%)* |
| MpCathB5 | MYZPE13164_G006_v1.0_000104270 | 335 | 1.00E-91 | Yes | 212/242(88%)* | 10/242 (4%)* |
| MpCathB3 | MYZPE13164_G006_v1.0_000141010 | 286 | 7.00E-77 | Yes | 208/242(86%) | 0/242 (0%) |
| MpCathB1 | MYZPE13164_G006_v1.0_000141000 | 286 | 7.00E-77 | Yes | 208/242(86%) | 0/242 (0%) |
| MpCathB2 | MYZPE13164_G006_v1.0_000049160 | 281 | 3.00E-75 | Yes | 207/242(86%) | 0/242 (0%) |
| MpCathB6 | MYZPE13164_G006_v1.0_000104300 | 250 | 6.00E-66 | Yes | 194/232(84%) | 0/232 (0%) |
| MpCathB7 | MYZPE13164_G006_v1.0_000104310 | 244 | 3.00E-64 | Yes | 193/232(83%) | 0/232 (0%) |
| MpCathB9 | MYZPE13164_G006_v1.0_000104280 | 177 | 5.00E-44 | Yes | 169/217(78%) | 0/217 (0%) |
| MpCathB10 | MYZPE13164_G006_v1.0_000195260 | 157 | 5.00E-38 | Yes | 134/174(77%)* | 0/174 (0%)* |
| MpCathB11 | MYZPE13164_G006_v1.0_000195260 | 157 | 5.00E-38 | Yes | 158/206(77%)* | 0/206 (0%)* |
| MpCathB8 | MYZPE13164_G006_v1.0_000104290 | 153 | 6.00E-37 | Yes | 143/182(79%) | 0/182 (0%) |
| MpCathB12 | MYZPE13164_G006_v1.0_000104320 | 137 | 5.00E-32 | No | 176/241(73%) | 3/241 (1%) |
| MpCathB13 | MYZPE13164_G006_v1.0_000104330 | 88.5 | 3.00E-17 | No | 108/148(73%) | 0/148 (0%) |
| MpCathB17 | MYZPE13164_G006_v1.0_000151060 | 44.9 | 4.00E-04 | No | 74/106 (69%) | 0/107 (0%) |

Alignment of 242bp with target geens


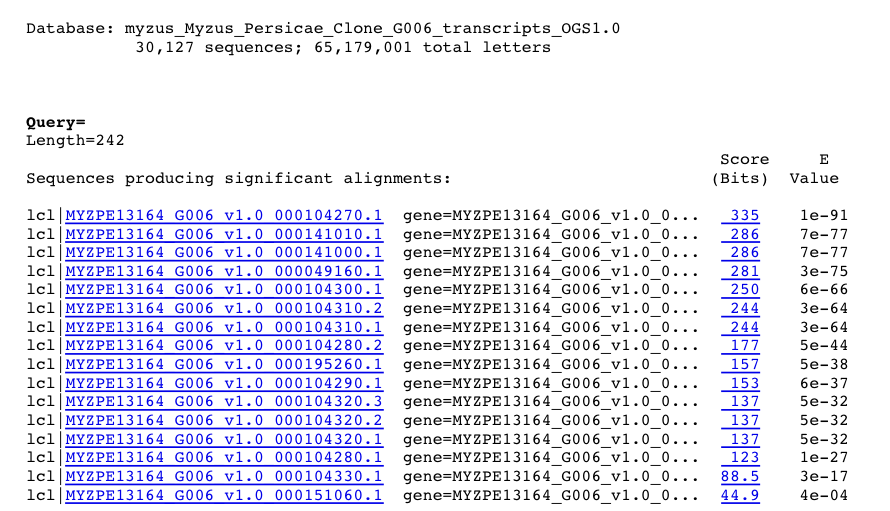


**MpCathB4/MpCathB5 MYZPE13164_G006_v1.0_000104270**


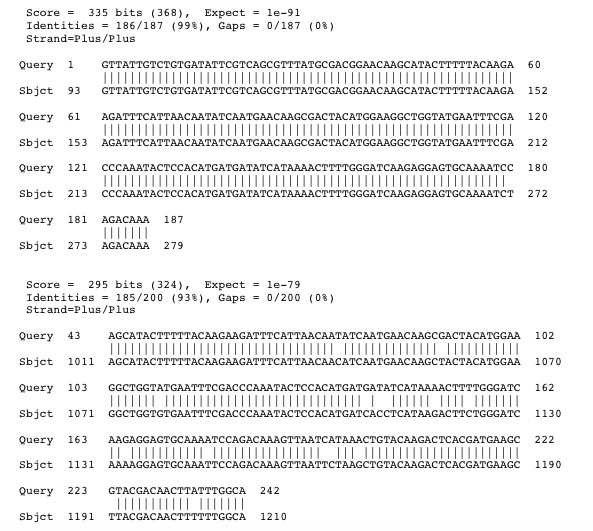


**MpCathB4 MYZPE13164_G006_v1.0_000104270**

Generated by NCBI blastn suite-2sequences


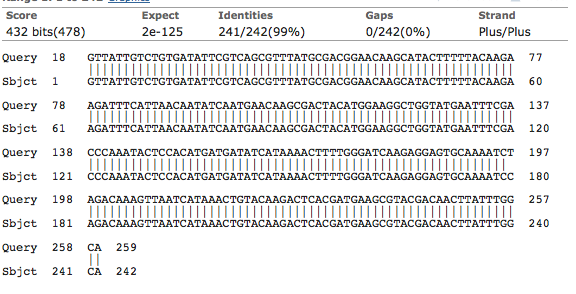


**MpCathB5 MYZPE13164_G006_v1.0_000104270**

Generated by NCBI blastn suite-2sequences


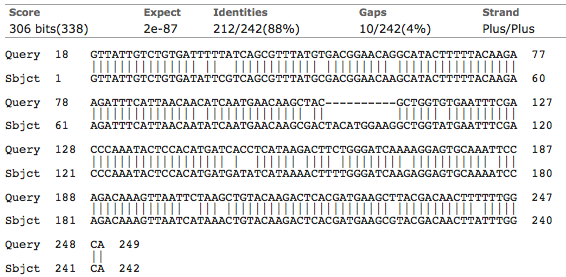


**MpCathB3 MYZPE13164_G006_v1.0_000141010**


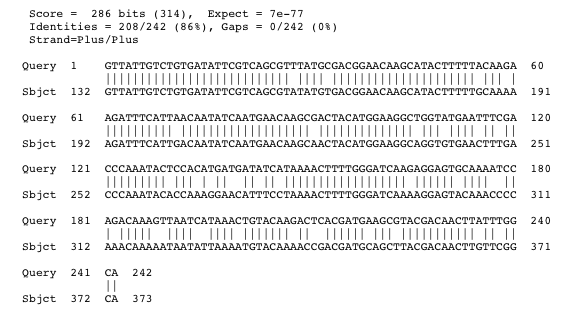


**MpCathB1 MYZPE13164_G006_v1.0_000141000**


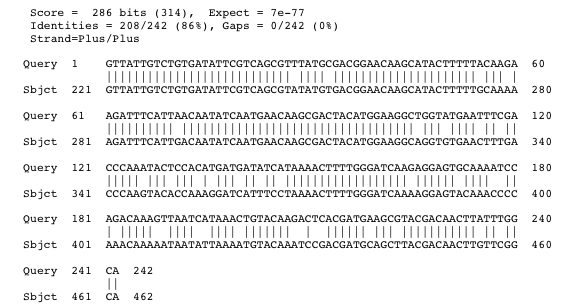


**MpCathB2 MYZPE13164_G006_v1.0_000049160**


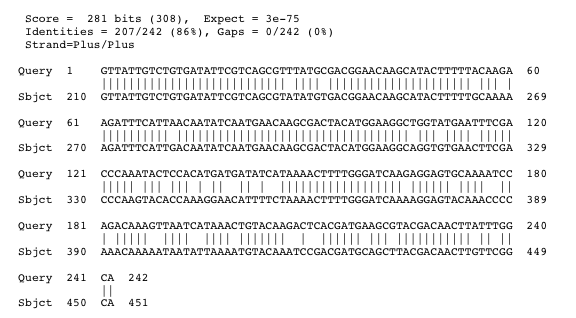


**MpCathB6 MYZPE13164_G006_v1.0_000104300**


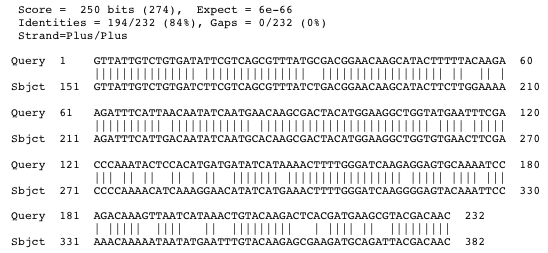


**MpCathB7 MYZPE13164_G006_v1.0_000104310**


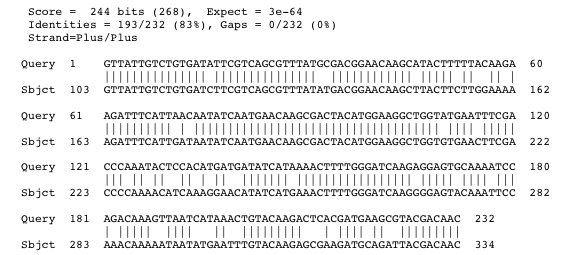


**MpCathB9 MYZPE13164_G006_v1.0_000104280**


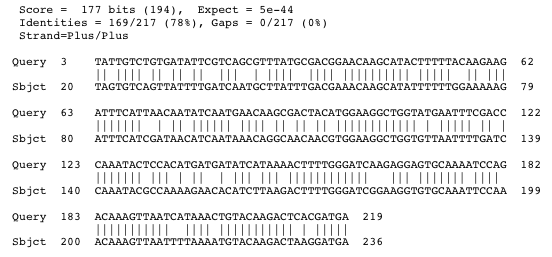


**MpCathB10/MpCathB111 MYZPE13164_G006_v1.0_000195260**


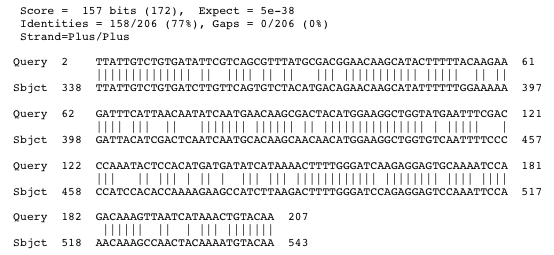


**MpCathB10 MYZPE13164_G006_v1.0_000195260**

Generated by NCBI blastn suite-2sequences


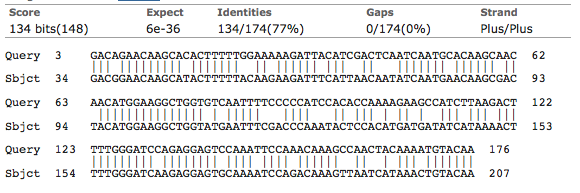


**MpCathB11 MYZPE13164_G006_v1.0_000195260**

Generated by NCBI blastn suite-2sequences


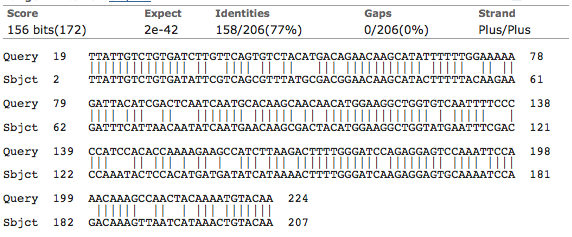


**MpCathB8 MYZPE13164_G006_v1.0_000104290**


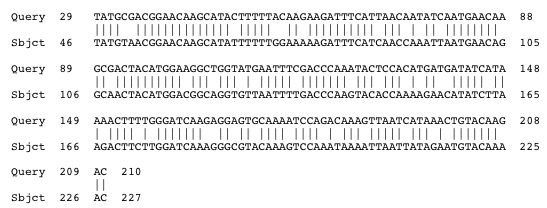


**MpCathB12 MYZPE13164_G006_v1.0_000104320**


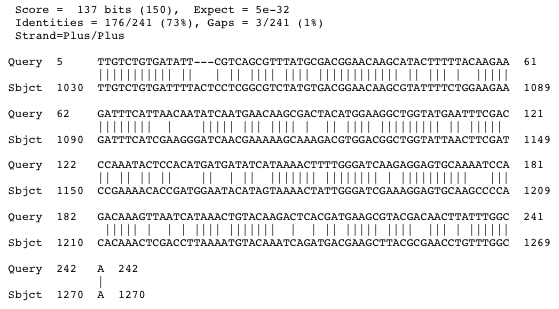


**MpCathB13 MYZPE13164_G006_v1.0_000104330**


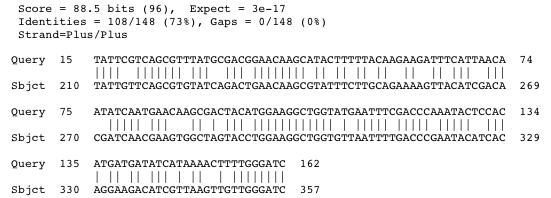


**MpCathB17 MYZPE13164_G006_v1.0_000151060**


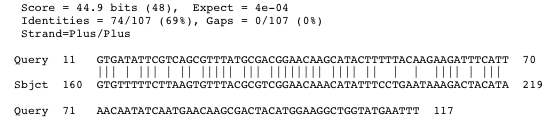

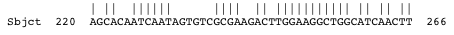

Supplement: Additional file 19: — Cathepsin B dsRNA alignment. Blast search results of the cathepsin B dsRNA sequence used to generate transgenic lines for plant-mediated RNAi of GPA. The 242-bp fragment of MpCathB4 (Clone O) was blastn-searched against the annotated genome of M. persicae clones G006. Identities and gaps of 242 bp with MpCathB4, MpCathB5, MpCath10 and MPCathB11 (indicated with *) were generated by NCBI blastn suite-2 sequences because these were misannotated in MyzsDB (G006). (DOCX 1108 kb) [file 13059_2016_1145_MOESM19_ESM.docx]
