# Supplementary material for: Rapid transcriptional plasticity of duplicated gene clusters enables a clonally reproducing aphid to colonise diverse plant species
Source: Genome Biol. 2017 Feb 13;18:27. doi: 10.1186/s13059-016-1145-3 (PMC5304397; doi:10.1186/s13059-016-1145-3)
Supplement: Additional file 21: Figure S11. — Domain analysis of aphid-specific cathepsin B genes. Protein sequences were used for analysis in InterPro. Clade highlighted in light green is aphid-specific clade I and the blue is aphid-specific clade II. Asterisks (*) indicate cathepsin B with complete domains, green asterisks are M. persicae cathepsins B, blue asterisks are the A. pisum ones and red asterisks are the D. noxia ones. (PDF 420 kb) [file 13059_2016_1145_MOESM21_ESM.pdf]

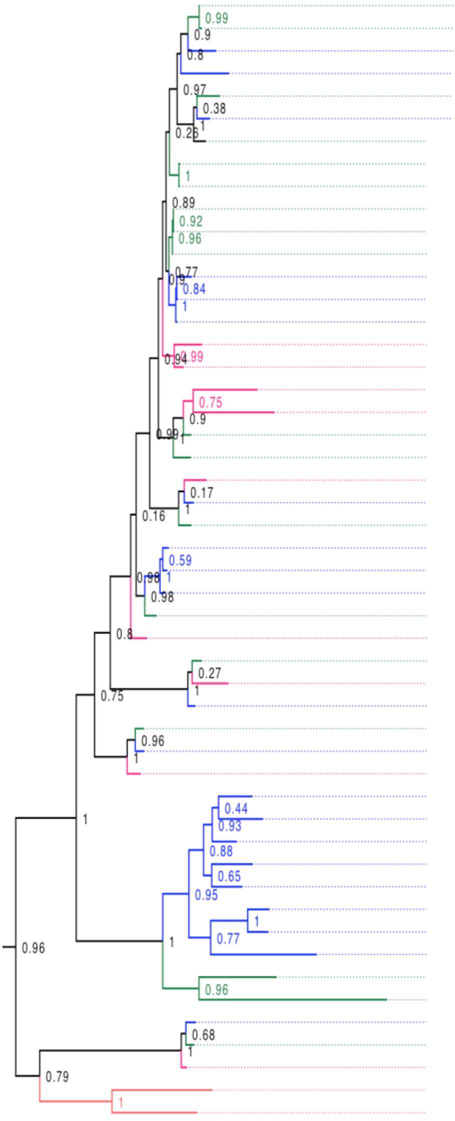

	F	D	D	S	S	
MpCathB11	+	+	+	+	+	*
MpCathB10	+	+	+	+	+	*
ACYPI063487	+	+	+		+	
ACYPI083038	+	+			+	
MpCathB9	+	+	+		+	
ACYPI000017	+	+	+		+	
MpCathB8	+	+	+		+	
MpCathB6	+	+	+	+	+	*
MpCathB7	+	+	+	+	+	*
MpCathB2	+	+	+	+	+	*
MpCathB1	+	+	+	+	+	*
MpCathB3	+	+	+	+	+	*
ACYPI010081	+	+	+	+	+	*
ACYPI001128	+	+	+	+	+	*
ACYPI000018	+	+	+	+	+	*
Dn_409_1.1	+	+	+	+		
Dn_409_1.2	+	+		+	+	
Dn_1855_0.0	+	+		+		
Dn_2923_0.0	+	+		+		
MpCathB4	+	+	+	+	+	*
MpCathB5	+	+	+	+	+	*
Dn_409_1.10	+	+	+	+	+	*
ACYPI000008	+	+	+	+	+	*
MpCathB12	+	+	+	+	+	*
ACYPI000013	+	+		+	+	
ACYPI52702	+	+	+	+	+	*
ACYPI005957	+	+	+	+	+	*
MpCathB13	+	+	+	+	+	*
Dn_409_1.5	+	+	+	+	+	*
MpCathB17	+	+	+		+	
Dn_1043_0.2	+	+			+	
ACYPI000006	+	+	+			
MpCathB16	+	+	+	+	+	*
ACYPI000014	+	+	+	+	+	*
DN_44_7.4	+	+	+	+	+	*
ACYPI002016	+	+				
ACYPI005139	+	+				
ACYPI004097	+	+				
ACYPI000012	+	+				
ACYPI001175	+	+				
ACYPI000497	+	+				
ACYPI002387	+	+				
ACYPI001191	+	+				
MpCathB14	+	+				
MpCathB15	+	+				
ACYPI000009	+	+				
MpCathB18	+	+				
Dn_789_0.0	+	+				
Dcitri_XP_008471034.1	+	+	+			
Dcitri_XP_008467581.1	+	+	+			

		InterPro	Discription
F	Family	IPR013128	Peptidase C1A
D	Domain	IPR000668	Peptidase C1A, papain C-terminal
D	Domain	IPR012599	Peptidase C1A, propeptide
S	Sites	IPR000169	Cysteine peptidase, cysteine active site
S	Sites	IPR025660	Cysteine peptidase, histidine active site
