# Supplementary material for: Rapid transcriptional plasticity of duplicated gene clusters enables a clonally reproducing aphid to colonise diverse plant species
Source: Genome Biol. 2017 Feb 13;18:27. doi: 10.1186/s13059-016-1145-3 (PMC5304397; doi:10.1186/s13059-016-1145-3)
Supplement: Additional file 23: Table S8. — List of cathepsin B genes annotated in the genome of the pea aphid A. pisum. (DOCX 125 kb) [file 13059_2016_1145_MOESM23_ESM.docx]

**Table S8**: List of cathepsin B genes annotated in the genome of the pea aphid *Acyrthosiphon pisum*

| ACYPI Number | Protein Name | Scaffold | position | NCBI RefeSeq | Abbreviation |
| --- | --- | --- | --- | --- | --- |
| ACYPI000003 | cathepsin B 348 | GL349839 | 648587-668442 | NP_001119608.1 | ApCathB_348 |
| ACYPI000013 | cathepsin B 16A | GL349633 | 913407-915326 | NP_001119617.1 | ApCathB_16A |
| ACYPI52702 | cathepsin B 16C | GL349633 | 930233-957254 | ABV03122.1 | ApCathB_16C |
| ACYPI001128 | cathepsin B 16D1 | GL350248 | 112726-115208 | ABV03123.1 | ApCathB_16D1 |
| ACYPI000009 | cathepsin B 2744 | GL350544 | 12746-16000 | DAA06100.1 | ApCathB_2744 |
| ACYPI000017 | cathepsin B 3098 | GL350248 | 94366-105369 | ABV03132.1 | ApCathB_3098 |
| ACYPI000014 | cathepsin B 84 | GL349877 | 52636-55859 | ABV03134.1 | ApCathB_84 |
| ACYPI000018 | cathepsin B 16D2 | GL350248 | 107545-111310 | DAA06103.1 | ApCathB_16D2 |
| ACYPI000021 | Cathepsin B 10270 | GL349938 | 114397-120690 | DAA06104.1 | ApCathB_10270 |
| ACYPI000012 | Cathepsin B 1674 | GL349899 | 841179-871477 |  | ApCathB_1674 |
| ACYPI000006 | Cathepsin B 1852 | GL349699 | 52873-60682 | DAA06106.1 | ApCathB_1852 |
| ACYPI000019 | Cathepsin B 1874 | GL349938 | 120738-125015 | DAA06107.1 | ApCathB_1874 |
| ACYPI000008 | Cathepsin B 912 | GL349633 | 872302-880805 | DAA06108.1 | ApCathB_912 |
| ACYPI080877 | Cathepsin B 3483 | GL349938 | 89728-97517 | DAA06109.1 | ApCathB_3483_2 |
| ACYPI005957 | cathepsin B 16A | GL349633 | 894389-902051 | NP_001119617.1 | ApCathB_16A |
| ACYPI000010 | cathepsin B 1418 | GL349709 | 803644-811984 | NP_001119614.1 | ApCathB_1418 |
| ACYPI010081 | cathepsin B-like cysteine proteinase 4 | GL350248 | 51706-53751 | XP_001950562.1 | ApCathB_L4_1 |
| ACYPI000015 | Cathepsin B 5880 | GL349938 | 100204-107589 | NP_001119619.1 | ApCathB_5880 |
| ACYPI001281 | cathepsin B-like cysteine proteinase 4 | GL350037 | 118905-122392 | XP_001943652.1 | ApCathB_L4_2 |
| ACYPI000020 | cathepsin B 3483 | GL360242 | 393-3821 | NP_001128426.1 | ApCath_3483_1 |
| ACYPI063487 | cathepsin B N | GL349737 | 587623-598161 | AAU84926.1 | ApCathB_N |
| ACYPI002387 | cathepsin B-like cysteine proteinase 3 | GL349627 | 156766-161936 | XP_001945855.1 | ApCathB_L3_1 |
| ACYPI Number | Protein Name | Scaffold | position | NCBI RefeSeq | Abbreviation |
| ACYPI000497 | cathepsin B-like cysteine proteinase 3 | GL349627 | 137056-152179 | XP_001945899.1 | ApCathB_L3_2 |
| ACYPI004097 | cathepsin B-like cysteine proteinase 5 | GL349672 | 186682-194830 | XP_008178596.1 | ApCathB_L5 |
| ACYPI005139 | cathepsin B-like cysteine proteinase 3 | GL350513 | 148769-153114 | XP_001948185.1 | ApCathB_L3_3 |
| ACYPI002016 | cathepsin B-like | GL350633 | 119845-126358 | XP_001944624.1,XP_008187496.1,XP_008187497.1 | ApCathB_L_1 |
| ACYPI001175 | cathepsin B | GL349623 | 1863715-1873259 |  | ApCathB_1 |
| ACYPI001191 | Cathepsin B-like | GL349689 | 173926-176565 | XP_001943280.2 | ApCathB_L_2 |
| ACYPI000485 | Cathepsin B | GL349963 | 305126-327473 |  | ApCathB_2 |
| ACYPI083038 | Cathepsin B-S | GL350248 | 46996-48285 |  | ApCathB_S |
