# Supplementary material for: Rapid transcriptional plasticity of duplicated gene clusters enables a clonally reproducing aphid to colonise diverse plant species
Source: Genome Biol. 2017 Feb 13;18:27. doi: 10.1186/s13059-016-1145-3 (PMC5304397; doi:10.1186/s13059-016-1145-3)
Supplement: Additional file 24: Table S9. — List of cathepsin B genes annotated in the genomes of Myzus persicae clones G006 and 0. Four fragments were annotated as MpCathB1 and two as MpCathB3 in Clone O. Alignment of fragments to corresponding genes indicated that fragments were part of the gene. The sequences of MpCathB1 and MpCathB3 in clone O were confirmed by PCR and sequencing. MpCathB2, MpCathB7 and MpCathB12 were missing in the genome annotation of M. persicae clone O; their sequences were confirmed by PCR and sequencing. (DOCX 112 kb) [file 13059_2016_1145_MOESM24_ESM.docx]

**Table S9**: List of cathepsin B genes annotated in the genomes of the green peach aphid *Myzus persicae* G006 and clone 0. Four fragments were annotated as MpCathB1 and two as MpCathB3 in Clone O. Alignment of fragments to corresponding genes indicated that fragments were part of the gene. The sequences of MpCathB1 and MpCathB3 in Clone O were confirmed by PCR and sequencing. MpCathB2, MpCathB7, and MpCathB12 were missing in the genome annotation of *M. persicae* clone O; their sequences were confirmed by PCR and sequencing.

| **Name** | **G006 ID** | **Scaffold** | **Start** | **End** | **Protein Length** | **Clon 0 ID** | **Scaffold** | **Start** | **End** | **Protein Length** | |
| --- | --- | --- | --- | --- | --- | --- | --- | --- | --- | --- | --- |
| MpCathB1 | MYZPE13164_G006_v1.0_000141000 | 491 | 198547 | 211898 | 340 | MYZPE13164_0_v1.0_000104620 | 3659 | 1 | 302 | 27 | Sequence identical with G006 |
|  |  |  |  |  |  | MYZPE13164_0_v1.0_000119280 | 4319 | 392 | 795 | 27 |  |
|  |  |  |  |  |  | MYZPE13164_0_v1.0_000082490 | 2776 | 9570 | 10029 | 27 |  |
|  |  |  |  |  |  | MYZPE13164_0_v1.0_000201750 | 9956 | 1 | 82 | 27 |  |
| MpCathB2 | MYZPE13164_G006_v1.0_000049160 | 158 | 51892 | 54045 | 340 | The gene in Clone O was confirmed by PCR and sequencing | | | | | |
| MpCathB3 | MYZPE13164_G006_v1.0_000141010 | 491 | 231009 | 237758 | 340 | MYZPE13164_0_v1.0_000177440 | 7921 | 1 | 2179 | 27 | Sequence identical with G006 |
|  |  |  |  |  |  | MYZPE13164_0_v1.0_000196430 | 9491 | 56 | 1649 | 27 |  |
|  |  |  |  |  |  | MYZPE13164_0_v1.0_000201750 | 9956 | 1 | 82 | 27 |  |
| MpCathB4 | MYZPE13164_G006_v1.0_000104270.b | 304 | 36621 | 46475 | 335 | MYZPE13164_0_v1.0_000047190 | 1682 | 95901 | 98256 | 339 |  |
| MpCathB5 | MYZPE13164_G006_v1.0_000104270.a | 304 | 36621 | 46475 | 333 | MYZPE13164_0_v1.0_000047180 | 1682 | 88216 | 91489 | 337 |  |
| **Name** | **G006 ID** | **Scaffold** | **Start** | **End** | **Protein Length** | **Clon 0 ID** | **Scaffold** | **Start** | **End** | **Protein Length** | |
| MpCathB6 | MYZPE13164_G006_v1.0_000104300 | 304 | 72054 | 75345 | 338 | MYZPE13164_0_v1.0_000047150 | 1682 | 45682 | 59982 |  |  |
| MpCathB7 | MYZPE13164_G006_v1.0_000104310 | 304 | 77413 | 80171 | 338 | The gene in Clone O was confirmed by PCR and sequencing | | | | | |
| MpCathB8 | MYZPE13164_G006_v1.0_000104290 | 304 | 59524 | 65112 | 337 | MYZPE13164_0_v1.0_000047170 | 1682 | 66826 | 74807 | 383 |  |
| MpCathB9 | MYZPE13164_G006_v1.0_000104280 | 304 | 48726 | 52971 | 298 | MYZPE13164_0_v1.0_000047160 | 1682 | 54598 | 85665 | 298 |  |
| MpCathB10 | MYZPE13164_G006_v1.0_000195260B | 90 |  |  | 315 | MYZPE13164_0_v1.0_000044010 | 1591 | 16608 | 19250 | 316 |  |
| MpCathB11 | MYZPE13164_G006_v1.0_000195260A | 90 | 54115 | 66515 | 340 | MYZPE13164_0_v1.0_000043990 | 1591 | 6976 | 9897 | 330 |  |
| MpCathB12 | MYZPE13164_G006_v1.0_000104320 | 304 | 81041 | 89994 | 341 | The gene in Clone O was confirmed by PCR and sequencing | | | | | |
| MpCathB13 | MYZPE13164_G006_v1.0_000104330 | 304 | 100116 | 111526 | 340 | MYZPE13164_0_v1.0_000047140 | 1682 | 22506 | 34555 | 340 |  |
| MpCathB14 | MYZPE13164_G006_v1.0_000192170 | 88 | 691057 | 696237 | 351 | MYZPE13164_0_v1.0_000070570 | 2306 | 40931 | 45865 | 356 |  |
| MpCathB15 | MYZPE13164_G006_v1.0_000192180 | 88 | 704572 | 715651 | 418 | MYZPE13164_0_v1.0_000070580 | 2306 | 53966 | 55056 | 247 |  |
| MpCathB16 | MYZPE13164_G006_v1.0_000150170 | 550 | 46351 | 49337 | 335 | MYZPE13164_0_v1.0_000091070 | 313 | 88219 | 90926 | 335 |  |
| MpCathB17 | MYZPE13164_G006_v1.0_000151060 | 558 | 4932 | 22707 | 343 | MYZPE13164_0_v1.0_000184140 | 846 | 213734 | 230703 | 343 |  |
|  |  |  |  |  |  |  |  |  |  |  | |
| **Name** | **G006 ID** | **Scaffold** | **Start** | **End** | **Protein Length** | **Clon 0 ID** | **Scaffold** | **Start** | **End** | **Protein Length** | |
| MpCathB18 | MYZPE13164_G006_v1.0_000144080 | 508 | 88586 | 91921 | 323 | MYZPE13164_0_v1.0_000041870 | 154 | 70692 | 74027 | 323 |  |
| MpCathB19 | MYZPE13164_G006_v1.0_000090040 | 258 | 164496 | 171961 | 346 | MYZPE13164_0_v1.0_000108240 | 384 | 33157 | 40355 | 337 |  |
| MpCathB20 | MYZPE13164_G006_v1.0_000007710 | 105 | 546584 | 553874 | 344 | MYZPE13164_0_v1.0_000060360 | 200 | 34580 | 39426 | 284 |  |
| MpCathB21 | MYZPE13164_G006_v1.0_000076080 | 213 | 395886 | 399706 | 340 | MYZPE13164_0_v1.0_000138900 | 534 | 50405 | 54218 | 340 |  |
| MpCathB22 | MYZPE13164_G006_v1.0_000142750 | 5 | 1637298 | 1643007 | 351 | MYZPE13164_0_v1.0_000118340 | 427 | 66485 | 73341 | 351 |  |
| MpCathB23 | MYZPE13164_G006_v1.0_000142710 | 5 | 1596561 | 1601534 | 341 | MYZPE13164_0_v1.0_000118300 | 427 | 29156 | 32428 | 341 |  |
| MpCathB24 | MYZPE13164_G006_v1.0_000069730 | 2 | 1363328 | 1371731 | 489 | MYZPE13164_0_v1.0_000137620 | 526 | 144347 | 151271 | 489 |  |
| MpCathB25 | MYZPE13164_G006_v1.0_000069720 | 2 | 1279805 | 1318050 | 461 | MYZPE13164_0_v1.0_000137610 | 526 | 61710 | 100005 | 461 |  |
| MpCathB26 | MYZPE13164_G006_v1.0_000142700 | 5 | 1591761 | 1595503 | 317 | MYZPE13164_0_v1.0_000118290 | 427 | 22556 | 26341 | 317 |  |
| MpCathB27 | MYZPE13164_G006_v1.0_000142740 | 5 | 1631517 | 1635902 | 302 | MYZPE13164_0_v1.0_000118330 | 427 | 60743 | 65128 | 302 |  |
