# Supplementary material for: Rapid transcriptional plasticity of duplicated gene clusters enables a clonally reproducing aphid to colonise diverse plant species
Source: Genome Biol. 2017 Feb 13;18:27. doi: 10.1186/s13059-016-1145-3 (PMC5304397; doi:10.1186/s13059-016-1145-3)
Supplement: Additional file 27: Table S12. — Summary of the manual annotation and gene edition of M. persicae (clone G006) CPR as described in Additional file 3. (DOCX 129 kb) [file 13059_2016_1145_MOESM27_ESM.docx]

**Table S12**: Summary of the manual annotation and gene edition of *M. persicae* (clone G006) CPR as described in Additional File 3.

| **Family Type Description (CPFP)** | **Official Gene set 1.0 (clone G006)** | **Manual annotation** | **Name** | **Edited gene name (clone G006)** | **A. pisum ortholog** | **A. pisum best hit** | **E-value (blast)** |
| --- | --- | --- | --- | --- | --- | --- | --- |
| CPR_RR-1 | MYZPE13164_G006_v1.0_000086100.1 | NO | G006_v1.0_000086100 |  | ACYPI066082-PA | ACYPI066082-PA | 3.00E-106 |
| CPR_RR-1 | MYZPE13164_G006_v1.0_000086090.1 | NO | G006_v1.0_000086090 |  | ACYPI001610-PA | ACYPI001610-PA | 1.00E-84 |
| CPR_RR-1 | MYZPE13164_G006_v1.0_000145040.1 | NO | G006_v1.0_000145040 |  | ACYPI006250-PA | ACYPI006250-PA | 4.00E-150 |
| CPR_RR-1 | MYZPE13164_G006_v1.0_000097300.1 | NO | G006_v1.0_000097300 |  | ACYPI37842-PA | ACYPI37842-PA | 7.00E-79 |
| CPR_RR-1 | MYZPE13164_G006_v1.0_000097270.1 | NO | G006_v1.0_000097270 |  | ACYPI002781-PA | ACYPI002781-PA | 4.00E-96 |
| CPR_RR-1 | MYZPE13164_G006_v1.0_000189920.1 | NO | G006_v1.0_000189920 |  | ACYPI007152-PA | ACYPI007152-PA | 1.00E-60 |
| CPR_RR-1 | MYZPE13164_G006_v1.0_000192600.1 | NO | G006_v1.0_000192600 |  | ACYPI002877-PA, ACYPI000308-PA | ACYPI000308-PA | 4.00E-107 |
| CPR_RR-1 | MYZPE13164_G006_v1.0_000005270.1 | YES |  | G006_v1.0_000005270_MANUAL |  | ACYPI006276-PA | 4.00E-96 |
| CPR_RR-1 | MYZPE13164_G006_v1.0_000055990.1 | NO | G006_v1.0_000055990 |  | ACYPI009006-PA | ACYPI009006-PA | 7.00E-79 |
| CPR_RR-1 | MYZPE13164_G006_v1.0_000086040.1 | NO | G006_v1.0_000086040 |  | ACYPI005591-PA | ACYPI066082-PA | 5.00E-15 |
| CPR_RR-1 | MYZPE13164_G006_v1.0_000086060.1 | NO | G006_v1.0_000086060 |  | ACYPI003649-PA | ACYPI008113-PA | 1.00E-60 |
| CPR_RR-1 | MYZPE13164_G006_v1.0_000086070.1 | NO | G006_v1.0_000086070 |  |  | ACYPI003649-PA | 3.00E-72 |
| CPR_RR-1 | MYZPE13164_G006_v1_0_000086050.1 | NO | G006_v1_0_000086050 |  |  | ACYPI008113-PA | 3.00E-167 |
| CPR_RR-2 | MYZPE13164_G006_v1.0_000085620.1 | NO | G006_v1.0_000085620 |  |  | ACYPI29043-PA | 3.00E-72 |
| CPR_RR-2 | MYZPE13164_G006_v1.0_000109560.1 | YES |  | G006_v1.0_000109560_MANUAL |  | ACYPI072943-PA | 3.00E-58 |
| CPR_RR-2 | MYZPE13164_G006_v1.0_000122290.1 | YES |  | G006_v1.0_000122290_MANUAL_1 |  | ACYPI008534-PA | 4.00E-78 |
| CPR_RR-2 | MYZPE13164_G006_v1.0_000122290.1 | YES |  | G006_v1.0_000122290_MANUAL_2 |  | ACYPI006670-PA | 8.00E-69 |
| CPR_RR-2 | MYZPE13164_G006_v1.0_000122340.1 | YES |  | G006_v1.0_000122340_MANUAL |  | ACYPI004810-PA | 1.00E-118 |
| CPR_RR-2 | MYZPE13164_G006_v1.0_000122410.1 | YES |  | G006_v1.0_000122410_MANUAL |  | ACYPI56614-PA | 4.00E-73 |
| CPR_RR-2 | MYZPE13164_G006_v1.0_000154160.1 | YES |  | G006_v1.0_000154160_MANUAL | ACYPI56307-PA | ACYPI56307-PA | 1.00E-42 |
| CPR_RR-2 | MYZPE13164_G006_v1.0_000160430.1 | YES |  | G006_v1.0_000160430_MANUAL |  | ACYPI004893-PA | 1.00E-51 |
| CPR_RR-2 | MYZPE13164_G006_v1.0_000167270.1 | NO | G006_v1.0_000167270 |  |  | ACYPI081329-PA | 2.00E-75 |
| CPR_RR-2 | MYZPE13164_G006_v1.0_000167340.1 | YES |  | G006_v1.0_000167340_MANUAL |  | ACYPI26371-PA | 8.00E-96 |
| CPR_RR-2 | MYZPE13164_G006_v1.0_000170330.1 | YES |  | G006_v1.0_000170330_MANUAL |  | ACYPI006099-PA | 3.00E-48 |
| CPR_RR-2 | MYZPE13164_G006_v1.0_000015900.1 | NO | G006_v1.0_000015900 |  | ACYPI54769-PA | ACYPI084042-PA | 2.00E-23 |
| CPR_RR-2 | MYZPE13164_G006_v1.0_000015910.1 | NO | G006_v1.0_000015910 |  |  | ACYPI002106-PA | 1.00E-115 |
| CPR_RR-2 | MYZPE13164_G006_v1.0_000015920.2 | NO | G006_v1.0_000015920 |  |  | ACYPI084042-PA | 5.00E-23 |
| CPR_RR-2 | MYZPE13164_G006_v1.0_000015930.1 | NO | G006_v1.0_000015930 |  | ACYPI38155-PA | ACYPI000300-PA | 1.00E-16 |
| CPR_RR-2 | MYZPE13164_G006_v1.0_000017400.1 | NO | G006_v1.0_000017400 |  | ACYPI009152-PA | ACYPI009152-PA | 4.00E-42 |
| CPR_RR-2 | MYZPE13164_G006_v1.0_000040500.1 | NO | G006_v1.0_000040500 |  |  | ACYPI062520-PA | 5.00E-89 |
| CPR_RR-2 | MYZPE13164_G006_v1.0_000062500.1 | NO | G006_v1.0_000062500 |  | ACYPI52218-PA | ACYPI52218-PA | 1.00E-74 |
| CPR_RR-2 | MYZPE13164_G006_v1.0_000083210.1 | NO | G006_v1.0_000083210 |  | ACYPI001908-PA | ACYPI001908-PA | 9.00E-94 |
| CPR_RR-2 | MYZPE13164_G006_v1.0_000097280.1 | NO | G006_v1.0_000097280 |  |  | ACYPI007858-PA | 3.00E-180 |
| CPR_RR-2 | MYZPE13164_G006_v1.0_000107160.1 | NO | G006_v1.0_000107160 |  | ACYPI006175-PA | ACYPI006175-PA | 1.00E-57 |
| CPR_RR-2 | MYZPE13164_G006_v1.0_000107430.1 | NO | G006_v1.0_000107430 |  |  | ACYPI42531-PA | 3.00E-75 |
| CPR_RR-2 | MYZPE13164_G006_v1.0_000122240.1 | NO | G006_v1.0_000122240 |  | ACYPI004114-PA | ACYPI002964-PA | 5.00E-96 |
| CPR_RR-2 | MYZPE13164_G006_v1.0_000122250.1 | NO | G006_v1.0_000122250 |  |  | ACYPI002964-PA | 5.00E-96 |
| CPR_RR-2 | MYZPE13164_G006_v1.0_000122260.1 | NO | G006_v1.0_000122260 |  |  | ACYPI002964-PA | 5.00E-95 |
| CPR_RR-2 | MYZPE13164_G006_v1.0_000122270.1 | NO | G006_v1.0_000122270 |  |  | ACYPI002964-PA | 5.00E-95 |
| CPR_RR-2 | MYZPE13164_G006_v1.0_000122300.1 | NO | G006_v1.0_000122300 |  |  | ACYPI082492-PA | 3.00E-53 |
| CPR_RR-2 | MYZPE13164_G006_v1.0_000122310.1 | NO | G006_v1.0_000122310 |  |  | ACYPI086655-PA | 1.00E-116 |
| CPR_RR-2 | MYZPE13164_G006_v1.0_000122320.1 | NO | G006_v1.0_000122320 |  |  | ACYPI56617-PA | 3.00E-112 |
| CPR_RR-2 | MYZPE13164_G006_v1.0_000122330.1 | NO | G006_v1.0_000122330 |  |  | ACYPI002964-PA | 3.00E-74 |
| CPR_RR-2 | MYZPE13164_G006_v1.0_000122350.1 | NO | G006_v1.0_000122350 |  | ACYPI000291-PA | ACYPI000291-PA | 2.00E-172 |
| CPR_RR-2 | MYZPE13164_G006_v1.0_000122360.1 | NO | G006_v1.0_000122360 |  |  | ACYPI006712-PA | 5.00E-46 |
| CPR_RR-2 | MYZPE13164_G006_v1.0_000122370.1 | NO | G006_v1.0_000122370 |  | ACYPI002889-PA | ACYPI004893-PA | 1.00E-82 |
| CPR_RR-2 | MYZPE13164_G006_v1.0_000122380.1 | NO | G006_v1.0_000122380 |  |  | ACYPI004893-PA | 4.00E-86 |
| CPR_RR-2 | MYZPE13164_G006_v1.0_000122390.1 | NO | G006_v1.0_000122390 |  | ACYPI004074-PA | ACYPI004893-PA | 4.00E-88 |
| CPR_RR-2 | MYZPE13164_G006_v1.0_000122400.1 | NO | G006_v1.0_000122400 |  |  | ACYPI006015-PA | 1.00E-80 |
| CPR_RR-2 | MYZPE13164_G006_v1.0_000124440.1 | NO | G006_v1.0_000124440 |  | ACYPI51165-PA | ACYPI51165-PA | 1.00E-74 |
| CPR_RR-2 | MYZPE13164_G006_v1.0_000129700.1 | NO | G006_v1.0_000129700 |  | ACYPI32811-PA | ACYPI32811-PA | 7.00E-57 |
| CPR_RR-2 | MYZPE13164_G006_v1.0_000129710.1 | NO | G006_v1.0_000129710 |  | ACYPI32812-PA | ACYPI003003-PA | 9.00E-19 |
| CPR_RR-2 | MYZPE13164_G006_v1.0_000144510.1 | NO | G006_v1.0_000144510 |  |  | ACYPI001681-PA | 5.00E-72 |
| CPR_RR-2 | MYZPE13164_G006_v1.0_000152880.1 | NO | G006_v1.0_000152880 |  |  | ACYPI42531-PA | 2.00E-71 |
| CPR_RR-2 | MYZPE13164_G006_v1.0_000152900.1 | NO | G006_v1.0_000152900 |  |  | ACYPI010212-PA | 2.00E-138 |
| CPR_RR-2 | MYZPE13164_G006_v1.0_000154130.1 | NO | G006_v1.0_000154130 |  | ACYPI008156-PA | ACYPI008156-PA | 6.00E-120 |
| CPR_RR-2 | MYZPE13164_G006_v1.0_000154140.1 | NO | G006_v1.0_000154140 |  | ACYPI003073-PA | ACYPI084042-PA | 3.00E-104 |
| CPR_RR-2 | MYZPE13164_G006_v1.0_000156510.1 | NO | G006_v1.0_000156510 |  | ACYPI001084-PA | ACYPI001084-PA | 5.00E-63 |
| CPR_RR-2 | MYZPE13164_G006_v1.0_000161670.1 | NO | G006_v1.0_000161670 |  |  | ACYPI009701-PA | 2.00E-135 |
| CPR_RR-2 | MYZPE13164_G006_v1.0_000162570.1 | NO | G006_v1.0_000162570 |  |  | ACYPI002005-PA | 4.00E-48 |
| CPR_RR-2 | MYZPE13164_G006_v1.0_000167260.1 | NO | G006_v1.0_000167260 |  |  | ACYPI004893-PA | 1.00E-79 |
| CPR_RR-2 | MYZPE13164_G006_v1.0_000167280.1 | NO | G006_v1.0_000167280 |  | ACYPI080968-PA | ACYPI004893-PA | 2.00E-81 |
| CPR_RR-2 | MYZPE13164_G006_v1.0_000167290.1 | NO | G006_v1.0_000167290 |  | ACYPI005387-PA | ACYPI005387-PA | 2.00E-70 |
| CPR_RR-2 | MYZPE13164_G006_v1.0_000167300.1 | NO | G006_v1.0_000167300 |  | ACYPI007329-PA | ACYPI007329-PA | 2.00E-69 |
| CPR_RR-2 | MYZPE13164_G006_v1.0_000167310.1 | NO | G006_v1.0_000167310 |  | ACYPI35873-PA | ACYPI35873-PA | 1.00E-76 |
| CPR_RR-2 | MYZPE13164_G006_v1.0_000167320.1 | NO | G006_v1.0_000167320 |  |  | ACYPI009803-PA | 9.00E-71 |
| CPR_RR-2 | MYZPE13164_G006_v1.0_000167330.1 | NO | G006_v1.0_000167330 |  | ACYPI001599-PA | ACYPI004893-PA | 1.00E-66 |
| CPR_RR-2 | MYZPE13164_G006_v1.0_000168960.1 | NO | G006_v1.0_000168960 |  | ACYPI003003-PA | ACYPI003003-PA | 5.00E-81 |
| CPR_RR-2 | MYZPE13164_G006_v1.0_000169000.1 | NO | G006_v1.0_000169000 |  | ACYPI008524-PA | ACYPI008524-PA | 2.00E-135 |
| CPR_RR-2 | MYZPE13164_G006_v1.0_000169020.1 | NO | G006_v1.0_000169020 |  | ACYPI000961-PA | ACYPI087743-PA | 2.00E-91 |
| CPR_RR-2 | MYZPE13164_G006_v1.0_000169030.1 | NO | G006_v1.0_000169030 |  |  | ACYPI000670-PA | 4.00E-67 |
| CPR_RR-2 | MYZPE13164_G006_v1.0_000169050.1 | NO | G006_v1.0_000169050 |  |  | ACYPI001095-PA | 2.00E-78 |
| CPR_RR-2 | MYZPE13164_G006_v1.0_000169060.1 | NO | G006_v1.0_000169060 |  | ACYPI23843-PA | ACYPI23843-PA | 2.00E-72 |
| CPR_RR-2 | MYZPE13164_G006_v1.0_000169070.1 | NO | G006_v1.0_000169070 |  |  | ACYPI000889-PA | 2.00E-68 |
| CPR_RR-2 | MYZPE13164_G006_v1.0_000177350.2 | NO | G006_v1.0_000177350 |  |  | ACYPI000849-PA | 1.00E-31 |
| CPR_RR-2 | MYZPE13164_G006_v1.0_000178160.1 | NO | G006_v1.0_000178160 |  |  | ACYPI003229-PA | 3.00E-79 |
| CPR_RR-3 | MYZPE13164_G006_v1.0_000047570.1 | NO | G006_v1.0_000047570 |  |  | ACYPI56625-PA | 5.00E-150 |
| CPR_RR-3 | MYZPE13164_G006_v1.0_000047580.1 | NO | G006_v1.0_000047580 |  |  | ACYPI009491-PA | 1.00E-74 |
