# Supplementary material for: Rapid transcriptional plasticity of duplicated gene clusters enables a clonally reproducing aphid to colonise diverse plant species
Source: Genome Biol. 2017 Feb 13;18:27. doi: 10.1186/s13059-016-1145-3 (PMC5304397; doi:10.1186/s13059-016-1145-3)
Supplement: Additional file 32: Table S17. — Models tested in the CAFE analysis of gene family evolution. Increasingly complex models of gene family evolution were tested using CAFE [78] with a focus on determining if aphid rates of gene gain and loss (gain = loss = λ) differ from that of other arthropod lineages. Regions of the arthropod phylogeny with different λ parameters were specified with the λ tree (newick format), which follows the species tree. For each model, five runs were conducted to check convergence. F.P. free parameters, Lh. likelihood, S.D. standard deviation. (DOCX 101 kb) [file 13059_2016_1145_MOESM32_ESM.docx]

**Table S17:** Models tested in the CAFE analysis of gene family evolution. Increasingly complex models of gene family evolution were tested using CAFE with a focus on determining if aphid rates of gene gain and loss (gain=loss=λ) differ from that of other arthropod lineages. Regions of the arthropod phylogeny with different λ parameters were specified with the λ tree (newick format), which follows the species tree. For each model, 5 runs were conducted to check convergence. Lh. = likelihood, S.D. = standard deviation**.**

| **Model** | **Description** | **λ tree** | **Best Lh.** | **Lh. S.D.*** | **R1** | **R2** | **R3** | **R4** | **R5** |
| --- | --- | --- | --- | --- | --- | --- | --- | --- | --- |
| 1 rate | Single rate across whole tree | (((((((((1,1)1,(1,1)1)1,(1,1)1)1,(1,(1,1)1)1)1,(1,1)1)1,(1,(1,(1,1)1)1)1)1,(1,(1,(1,1)1)1)1)1,1)1,1) | -114570.3 | 0.004 | -114570.3 | -114570.3 | -114570.3 | -114570.3 | -114570.3 |
| 2 rate | *A. pisum*, all other taxa | (((((((((2,2)2,(2,2)2)2,(2,2)2)2,(2,(2,2)2)2)2,(2,2)2)2,(2,(2,(2,2)2)2)2)2,(2,(2,(1,2)2)2)2)2,2)2,2) | -112113.8 | 0.002 | -112112.8 | -112112.9 | -112112.8 | -112112.8 | -112112.8 |
| 3 rate | *A. pisum*, *M. persicae*, all other taxa | (((((((((2,2)2,(2,2)2)2,(2,2)2)2,(2,(2,2)2)2)2,(2,2)2)2,(2,(2,(2,2)2)2)2)2,(2,(2,(3,1)2)2)2)2,2)2,2) | -111955.3 | 0.002 | -111955.3 | -111955.3 | -111955.3 | -111955.3 | -111955.3 |
| 4 rate | *A. pisum*, *M. persicae*, Daphnia, all other taxa | (((((((((2,2)2,(2,2)2)2,(2,2)2)2,(2,(2,2)2)2)2,(2,2)2)2,(2,(2,(2,2)2)2)2)2,(2,(2,(3,1)2)2)2)2,4)2,2) | -111954.1 | 0.002 | -111954.1 | -111954.1 | -111954.1 | -111954.1 | -111954.1 |
| Clade specific rates | Diptera, Coleoptera, Lepidoptera, Hymenoptera, Hemiptera, Daphnia, Tetranychus, *A. pisum* and *M. persicae*, basal branches | (((((((((1,1)1,(1,1)1)1,(1,1)1)1,(2,(2,2)2)2)3,(4,4)4)3,(5,(5,(5,5)5)5)5)3,(6,(6,(7,8)6)6)6)3,9)3,10) | -107824.1 | 0.005 | -107824.2 | -108081.4** | -107824.1 | -107824.1 | -107824.1 |

* Standard deviation of model likelihood between runs calculated based on likelihood scores given to three decimal places.

** Run 2 failed to reach global maximum likelihood and was omitted from standard deviation calculation. The other 4 runs had highly consistent likelihood scores.
