# Supplementary material for: Rapid transcriptional plasticity of duplicated gene clusters enables a clonally reproducing aphid to colonise diverse plant species
Source: Genome Biol. 2017 Feb 13;18:27. doi: 10.1186/s13059-016-1145-3 (PMC5304397; doi:10.1186/s13059-016-1145-3)
Supplement: Additional file 33: Table S18. — Likelihood ratio test results comparing models of gene family evolution estimated in CAFE [78]. Models tested are detailed in Additional file 32: Table S17. Likelihood ratio tests were conducted in a nested fashion comparing more complex models to less complex models. The best fitting model tested was the clade-specific rates model, which gave a significant increase in likelihood over all other simpler models. The difference in the number of free parameters between each model is shown below the shaded squares. The likelihood ratio and p value (in brackets) for each model comparison are shown to the right of the shaded squares. For each model the best likelihood score out of five runs was used to calculate the likelihood ratio. The likelihood ratio was calculated as follows: likelihood ratio = 2 × ((likelihood more complex model) – (likelihood less complex model)). p values for the likelihood ratio test were generated by comparing the likelihood ratio between the more complex model and the less complex model to a Chi-square distribution with the degrees of freedom equal to the difference in the number of free parameters between the two models. (DOCX 82 kb) [file 13059_2016_1145_MOESM33_ESM.docx]

**Table S18:** Likelihood ratio test results comparing models of gene family evolution estimated in CAFE. Models tested are detailed in Additional File 32: Table S17. Likelihood ratio tests were conducted in a nested fashion comparing more complex models to less complex models. The best fitting model tested was the clade specific rates model, which gave a significant increase in likelihood over all other more simple models. The difference in the number of free parameters between each model is shown below the shaded squares. The likelihood ratio and p value (in brackets) for each model comparison are shown to the right of the shaded squares. For each model the best likelihood score out of five runs was used to calculate the likelihood ratio. The likelihood ratio was calculated as follows: likelihood ratio = 2 x ((likelihood more complex model) – (likelihood less complex model)). p values for the likelihood ratio test were generated by comparing the likelihood ratio between the more complex model and the less complex model to a chi square distribution with the degrees of freedom equal to the difference in the number of free parameters between the two models.

| **Model** | **1 rate** | **2 rate** | **3 rate** | **4 rate** | **Clade specific rates** |
| --- | --- | --- | --- | --- | --- |
| **1 rate** |  | 4914.938  (< 0.0005) | 5230.047  (< 0.0005) | 5232.444  (< 0.0005) | 13492.343  (< 0.0005) |
| **2 rate** | 1 |  | 315.109  (1.684 x 10^-70^) | 317.505  (1.134 x 10^-69^) | 8577.405  (< 0.0005) |
| **3 rate** | 2 | 1 |  | 2.396  (0.122) | 8262.296  (< 0.0005) |
| **4 rate** | 3 | 2 | 1 |  | 8259.900  (< 0.0005) |
| **Clade specific rates** | 9 | 8 | 7 | 6 |  |
