# Supplementary material for: Rapid transcriptional plasticity of duplicated gene clusters enables a clonally reproducing aphid to colonise diverse plant species
Source: Genome Biol. 2017 Feb 13;18:27. doi: 10.1186/s13059-016-1145-3 (PMC5304397; doi:10.1186/s13059-016-1145-3)
Supplement: Additional file 35: Table S20. — Sequences of primers used in Gateway cloning and qRT-PCR experiments. (DOCX 130 kb) [file 13059_2016_1145_MOESM35_ESM.docx]

**Table S20:** Sequences of primers used in Gateway cloning and qRT-PCR experiments.

| Genes | Primers (5'-3') |
| --- | --- |
| MpCathB4 attB1 | GGGGACAAGTTTGTACAAAAAAGCAGGCTGTTATTGTCTGTGATATTCGTCAGC |
| MpCathB4 attB2 | GGGGACCACTTTGTACAAGAAAGCTGGGTGCCAAATAAGTTGTCGTACGCTTC |
| MpCathB1 Forward | ATGGAAGGCAGGTGTGAACTTT |
| MpCathB1 Reverse | TGAAATCTGCATTGGTCGCTACA |
| MpCathB2 Forward | ATGGAAGGCAGGTGTGAACTTC |
| MpCathB2 Reverse | TGAAATCTGCATTGGTCGCTACG |
| MpCathB3 Forward | GACAATATCAATGAACAAGCA |
| MpCathB3 Reverse | TCGTAAGCTGCATCGTCGGT |
| MpCathB4 Forward | GGTCTAGTAACTGGAGGAGAGT |
| MpCathB4 Reverse | CCGATGGTTGCTTTGAAGATG |
| MpCathB5 Forward | CGGTCTAGTAACTGGAGGAGAC |
| MpCathB5 Reverse | CCAACGGTTGATCTGAGGAT |
| MpCathB6 Forward | GTGATCTTCGTCAGCGTTTATC |
| MpCathB6 Reverse | TTCATATTATTTTTGTTTGGA |
| MpCathB7 Forward | CTTGGAAAAAGATTTCATTGAT |
| MpCathB7 Reverse | TTCATATTATTTTTGTTTGGA |
| MpCathB10 Forward | AATGTAAGACGATTGGAGCAGT |
| MpCathB10 Reverse | TTCATTGAAATCAGCGTCTGTTG |
| MpCathB11 Forward | GGCATTAGCTACAAGTTCA |
| MpCathB11 Reverse | CCACATGTGTGACAACAGAAAG |
| MpCathB12 Forward | CCCACACAAACTCGACCTTA |
| MpCathB12 Reverse | ACTCTCTCCTCGCATCAAAC |
| MpCathB16 Forward | ACAGCCAACCGAACGTAATC |
| MpCathB16 Reverse | GCTCAGGTAGTATGCGTCTTTC |
| MpCathB17 Forward | CGATCAGAGCTTGGCGATATTA |
| MpCathB17 Reverse | CTGGTTGACCGCTACAAGAA |
| MpCathB19 Forward | GTAACGGTGGTCATCCAGAAA |
| MpCathB19 Reverse | CATCCCTCGTTAGAAGCGTATT |
| MpCutP56 Forward | TTACCCGTCACCCTCTTACT |
| MpCutP56 Reverse | GTGGGTCGTTTACGCTATACTC |
| MpCutP46 Forward | CCTACGACATCAAGAGCCAATC |
| MpCutP46 Reverse | TGACATCATCAGCGGTGTATTC |
| MpCutP53 Forward | CAACCCGTGTCGTCGAATA |
| MpCutP53 Reverse | TACGGTGCCTTGTAACCTTG |
| MpCutP54 Forward | ACAACTTCGAGTACAGCGTAAA |
| MpCutP54 Reverse | TGTAAGTCCCTTTGACGTAACC |
| MpCutP51 Forward | CCCGTACAACTTCGAGTACAG |
| MpCutP51 Reverse | TGTACGATCCCTTGACGTTTC |
| MpCutP17 Forward | GCCAACATCATTGCTGGATTAG |
| MpCutP17 Reverse | GACGGAGCTGAGTATGAAGAAG |
| MpCutP43 Forward | CTCTGTCAACGACTACCAAACT |
| MpCutP43 Reverse | GCCTCGATTAGGCTGTAGTAAC |
| MpCutP55 Forward | CTTACTCTGCACCAGCTTACTC |
| MpCutP55 Reverse | CTTGTATGGTGCTGGCTTGTA |
| MpCutP52 Forward | CCCACAGTCGATGAGCATAAT |
| MpCutP52 Reverse | TGATCATCTTAGCGGCCATTT |
| MpCutP60 Forward | CTTACTCAGCACCAGCTTACTC |
| MpCutP60 Reverse | GTAGGTGTGTGGGTCGTTTAC |
| MpCutP63 Forward | ACTCAGCACCAAAGGCATAC |
| MpCutP63 Reverse | CGTAGGTGTGTGGATCGTTTAC |
| MpCutP49 Forward | TACGACGTCAAGAGCCAATC |
| MpCutP49 Reverse | TAGTTGTCGGCGGTGTATTC |
| MpCutP48 Forward | TCGGCACCAAAGGCATAC |
| MpCutP48 Reverse | GTATTCGGACTGGCTCTTGAC |
| MpCutP57 Forward | CGGTAACGTGAAAGGCTTCTA |
| MpCutP57 Reverse | GTATCCGCCTTCCTTCTTGAC |
| MpCutP62 Forward | CCTACGATGTCAAGAGCCAATC |
| MpCutP62 Reverse | TAGTCGTCAGCGGTGTATTCTA |
| MpCutP59 Forward | TACTCAGCACCAAAGGCTTAC |
| MpCutP59 Reverse | TCGGATTGGCTCTTGACATC |
| MpCutP45 Forward | CTCCGTACAACTTCGACTACAG |
| MpCutP45 Reverse | GTAGGAGCCCTTGACGTAAC |
